# New marine-derived indolymethyl pyrazinoquinazoline alkaloids with promising antimicrobial profiles†

**DOI:** 10.1039/d0ra05319h

**Published:** 2020-08-21

**Authors:** Solida Long, Diana I. S. P. Resende, Andreia Palmeira, Anake Kijjoa, Artur M. S. Silva, Maria Elizabeth Tiritan, Patrícia Pereira-Terra, Joana Freitas-Silva, Sandra Barreiro, Renata Silva, Fernando Remião, Eugénia Pinto, Paulo Martins da Costa, Emília Sousa, Madalena M. M. Pinto

**Affiliations:** LQOF – Laboratório de Química Orgânica e Farmacêutica, Departamento de Ciências Químicas, Faculdade de Farmácia, Universidade do Porto Rua de Jorge Viterbo Ferreira, 228 4050-313 Porto Portugal esousa@ff.up.pt; CIIMAR – Centro Interdisciplinar de Investigação Marinha e Ambiental, Terminal de Cruzeiros do Porto de Leixões Av. General Norton de Matos S/N 4450-208 Matosinhos Portugal; ICBAS – Instituto de Ciências Biomédicas Abel Salazar, Universidade do Porto Rua de Jorge Viterbo Ferreira, 228 4050-313 Porto Portugal pmcosta@icbas.up.pt; QOPNA – Química Orgânica, Produtos Naturais e Agroalimentares, Departamento de Química, Universidade de Aveiro 3810-193 Aveiro Portugal; CESPU, Instituto de Investigação e Formação Avançada em Ciências e Tecnologias da Saúde (IINFACTS) Rua Central de Gandra, 1317 4585-116 Gandra PRD Portugal; UCIBIO-REQUIMTE, Laboratório de Toxicologia, Departamento de Ciências Biológicas, Faculdade de Farmácia, Universidade do Porto Rua de Jorge Viterbo Ferreira, 228 4050-313 Porto Portugal; Laboratório de Microbiologia, Departamento de Ciências Biológicas, Faculdade de Farmácia, Universidade do Porto Rua de Jorge Viterbo Ferreira, 228 4050-313 Porto Portugal

## Abstract

Due to the emergence of multidrug-resistant pathogenic microorganisms, the search for novel antimicrobials is urgent. Inspired by marine alkaloids, a series of indolomethyl pyrazino [1,2-*b*]quinazoline-3,6-diones was prepared using a one-pot microwave-assisted multicomponent polycondensation of amino acids. The compounds were evaluated for their antimicrobial activity against a panel of nine bacterial strains and five fungal strains. Compounds 26 and 27 were the most effective against *Staphylococcus aureus* ATCC 29213 reference strain with MIC values of 4 μg mL^−1^, and a methicillin-resistant *Staphylococcus aureus* (MRSA) isolate with MIC values of 8 μg mL^−1^. It was possible to infer that enantiomer (−)-26 was responsible for the antibacterial activity (MIC 4 μg mL^−1^) while (+)-26 had no activity. Furthermore, compound (−)-26 was able to impair *S. aureus* biofilm production and no significant cytotoxicity towards differentiated and non-differentiated SH-SY5Y cells was observed. Compounds 26, 28, and 29 showed a weak antifungal activity against *Trichophyton rubrum* clinical isolate with MIC 128 μg mL^−1^ and presented a synergistic effect with fluconazole.

## Abstract 2

Indolomethyl pyrazino [1,2-*b*]quinazoline-3,6-diones were prepared using a one-pot multicomponent polycondensation of amino acids and were evaluated for their antimicrobial activity against a panel of nine bacterial strains and five fungal strains.

## Graphical abstract

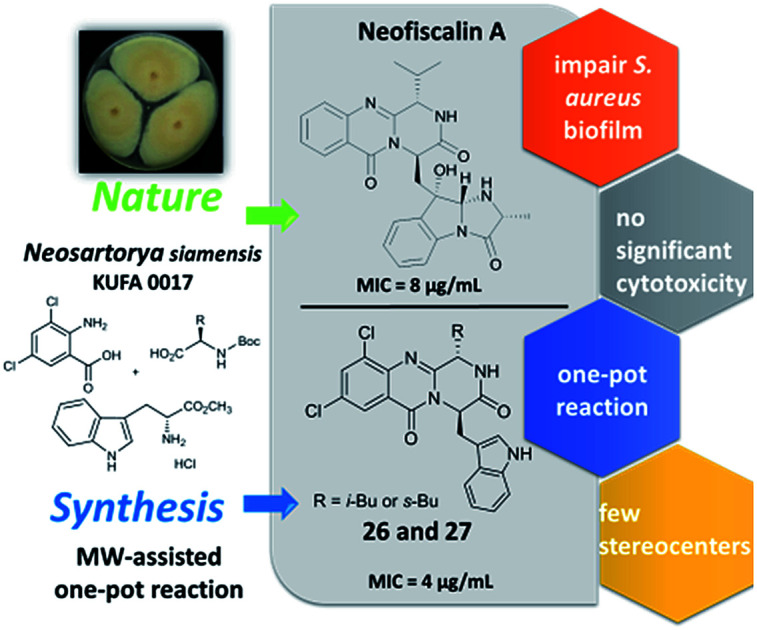

## Introduction

Infectious diseases caused by microorganisms stand as a major threat to public health.^1^ Since antibiotics were first introduced as medicines, these drugs have been used to prevent or treat infections in several applications.^2,3^ Nonetheless, antibacterial resistance has increased dramatically, becoming an emergency in healthcare during the last 40 years.^4–6^ Among 50 emerging infectious agents that have been identified, 10% have developed resistance to multiple drugs including antibiotics such as vancomycin,^7,8^ methicillin,^9^ carbapenems,^10^ and cephalosporins.^11–13^ Despite enormous efforts, the number of therapeutically useful compounds aiming to circumvent resistance is continuously decreasing and no truly novel class of compounds has been introduced into therapy, causing the World to face the “post-antibiotic era”.^14,15^ In order to restrain the clinical consequences of the development and spread of antimicrobial resistance both the preservation of current antimicrobials through their appropriate use and the discovery and development of new agents are mandatory.^16^

**Fig. 1:**
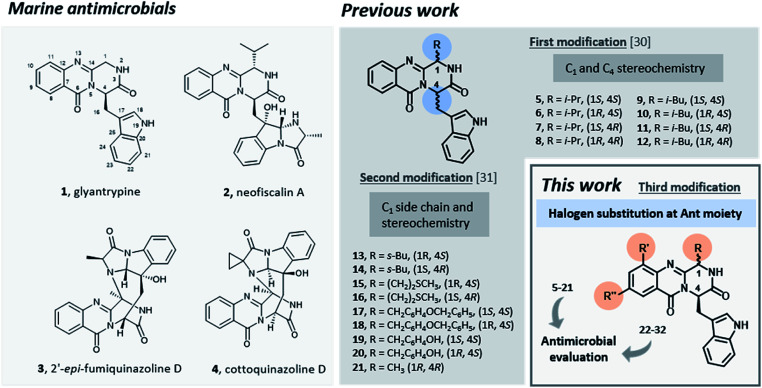
Marine antimicrobials 1–4 and rational of this work.

Promising antimicrobial agents for overcoming multidrug resistance are emerging from a variety of sources and methodologies,^17–23^ with the very recent example of artificial intelligence aiding this discovery.^24^ Several reports emphasized the discovery of new sophisticated antimicrobials from marine sources as a promising strategy to overcome the ever-increasing drug-resistant infectious diseases.^25–29^ Particularly, fungal alkaloids containing an indolomethyl pyrazino[1,2-*b*]quinazoline-3,6-dione scaffold were isolated from marine organisms and presented very interesting antimicrobial activities.^30^ For instance, glyantrypine (1, Fig. 1), isolated from *Cladosporium* sp. PJX-41, exhibited moderate inhibitory activity against a bacterium *Vibrio harveyi* (MIC = 32 μg mL^−1^)^31^ and neofiscalin A (2), obtained from*Neosartorya siamensis* KUFC 6349, exhibited a potent antibacterial activity against *Staphylococcus aureus* and *Enterococcus faecalis* (MIC = 8 μg mL^−1^).^32^ Regarding antifungal activity, fumiquinazoline F (3) for instance, obtained from a culture of *Aspergillus fumigatus* LN-4 showed good activity (MIC = 12.5–50 μg mL^−1^) against several plant pathogenic fungi,^33^ and cottoquinazoline D (4) obtained from *Aspergillus versicolor* LCJ-5-4, showed moderate antifungal activity against *Candida albicans* (MIC = 22.6 μM).^34^

Inspired by the relevant antibacterial activity of these quinazolinedione natural products^30^ and the fact that these pure alkaloids are obtained from the fugal culture in low yields,^35^ we have embarked on a synthesis of pyrazino[1,2-*b*]quinazoline-3,6-dione derivatives with a simpler indolomethyl moiety decreasing the number of stereogenic centers. Our first approach (5–12, Fig. 1) involved the synthesis of enantiomeric pairs of two members of this quinazolinone family (structural modifications at C-1 and C-4 stereochemistry), inspired by the marine-derived alkaloid fiscalin B (7).^36^ The second approach (13–21, Fig. 1) consisted in the synthesis of other derivatives of these natural alkaloids, but this time with modification of the C-1 side chain and the stereochemistry, by using different amino acids.^37^ Influenced by a large number of halogenated marine natural products with interesting antimicrobial activities isolated over the last few years,^29,38^ in the present work (22–32, Fig. 1) we present a third approach through the introduction of halogen atoms in the aromatic ring of anthranilic acid (Ant) which led to the discovery of promising antimicrobial agents within this series. The potential mechanism of action was studied using *in silico* docking on three potential antibacterial targets of antimicrobial alkaloids (*S. aureus* DNA gyrase B (GyrB), and *S. aureus* FtsZ), followed by molecular dynamics.

## Results and discussion

### Chemistry

The eleven new indolomethyl pyrazino[1,2-*b*]quinazoline-3,6-dione derivatives 22–32 were synthesized by a previously described approach using a microwave-assisted multicomponent polycondensation of amino acids with modifications (Table 1).^36,37,39^ The coupling of halogenated commercial anthranilic acids (33) to *N*-protected l-α-amino acids (34), and further dehydrative cyclization using triphenyl phosphite [(PhO)_3_P], generated a kind of benzoxazin-4-ones intermediates 35 which, followed by the addition of d-tryptophan methyl ester (36) under microwave irradiation, furnished the desirable final products 22–32 (2–14% yield) with partial epimerization (Table 1). Starting from halogenated anthranilic acid 33, the reaction conditions had to be optimized to obtain the intermediates 35: the amount of (PhO)_3_P was increased from 1.2 eq. to 2.4 eq. and the reaction time was increased from 16 to 24 h. With these conditions we were able to increase the scope of this one-pot reaction to halogenated compounds. Interestingly, only *anti* isomers (1*S*,4*R*) were obtained by this methodology whereas the different side chains at C-1 were achieved by selecting diverse l-α amino acids–valine, leucine, and isoleucine. The purity of the compounds was determined, by a reversed-phase liquid chromatography (RP-LC, C18, MeOH : H_2_O; 50 : 50), to be on average higher than 95%, whereas that for 25 and 32 was 90%.

**Table 1:** Synthesis of halogenated quinazolinone derivatives 22–32^a^

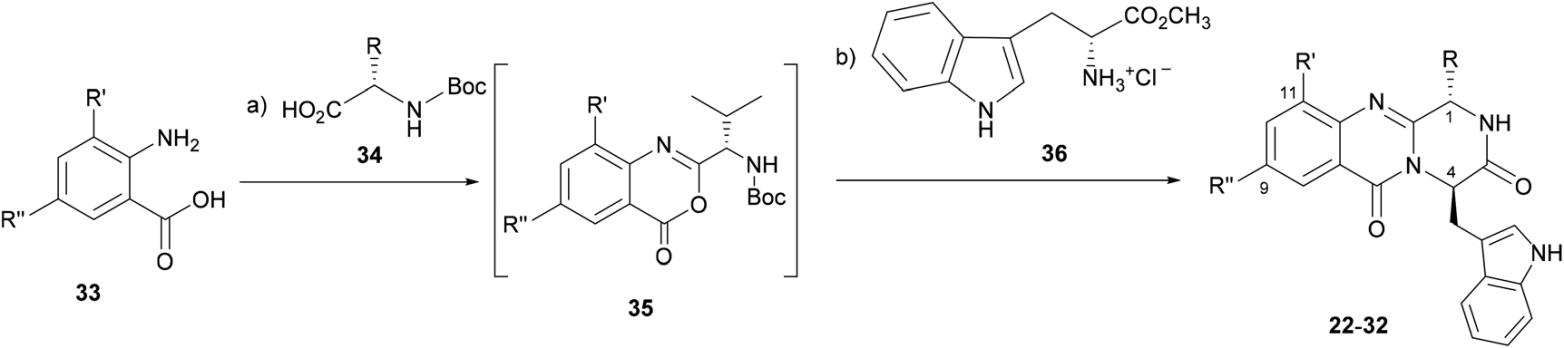							
Compound	R	R′	R′′	Yield (%)	[*α*]_D_^b^	er^c^	% purity^d^
22	*i*-Pr	H	Cl	5	−273	56 : 44	92
23	*i*-Bu	H	Cl	3	+154	44 : 56	>99
24	*s*-Bu	H	Cl	2	+130	46 : 54	93
25	*i*-Pr	Cl	Cl	5	+140	43 : 57	90
26	*i*-Bu	Cl	Cl	4.5	−169	60 : 40	>99
27	*s*-Bu	Cl	Cl	2.6	−264	71 : 29	>99
28	*i*-Pr	H	I	4.1	−175	51 : 49	95
29	*i*-Pr	H	Br	1.2	−170	50 : 50	95
30	*i*-Bu	H	I	11.8	−165	51 : 49	98
31	*i*-Bu	H	Br	13.8	−243	51 : 49	98
32	*i*-Bu	I	I	3.5	−229	54 : 46	90

a Reaction conditions: (a) dried-pyridine, (PhO)_3_P, 55 °C, 24 h; (b) dried-pyridine, (PhO)_3_P, 220 °C, 1.5 min.

b Optical rotation.

c er = enantiomeric ratio determined by enantioselective LC (column: amylose, Lux® 5 μm amylose-1, 250 × 4.6 mm, flow rate: 0.5 mL min^−1^, mobile phase: hexane : EtOH, 9 : 1).

d % purity determined by RP-LC.

Structure elucidation of compounds 22–32 was accomplished by 1D and 2D NMR (using CDCl_3_ or DMSO-d_6_ as solvents) spectral analysis and confirmed by HRMS. Particularly, the ^1^H NMR spectra of the monohalogenated derivatives, 22–24, and 28–31 whose halogen atoms (Cl, Br, or I) are at position 9, exhibited a signal corresponding to the resonance of H-8 in the form of a doublet at *δ*_H_*ca.* 8.5 with a small coupling constant (^4^*J* = 2.4 Hz), evidencing a long range coupling with H-10; while the signal of H-10 appeared as double doublets at *δ ca.* 7.9 (^3^*J* = 8.7 Hz and ^4^*J* = 2.4 Hz). In addition, the signal corresponding to the resonance of H-11 appeared as doublet at *δ ca.* 7.5 (^3^*J* = 8.7 Hz), indicating a short-range coupling to H-10. Similarly, the ^1^H NMR spectra of the dihalogenated derivatives, *i.e.*25–27 and 32, with two halogen atoms (Cl or I) at positions 9 and 11, also appeared as a doublet at *δ ca.* 7.76 (^4^*J* = 2.4 Hz) corroborating a long-range coupling between H-8 and H-10 (See in Material and methods).

It was observed also that the signals of the NH of the indole moiety (H-7′) and the amide group (H-2) appeared at higher frequencies when DMSO-d_6_ was used as a solvent. For example, for 25, 26, and 32, the signals H-2 and H-7′ appeared at *δ*_H_ 10.20 and 7.11 ppm, respectively, and this was due to the establishment of H-bonding (see the Experimental data).

### Microbiology

#### Antibacterial susceptibility testing

An initial screening of the antibacterial activity of 5-32 against different reference strains of Gram-positive, Gram-negative bacteria, and clinically relevant multidrug-resistant (MDR) strains was performed by the disk diffusion method.^40,41^ This primary assessment was followed by the determination of minimal inhibitory concentrations (MIC) of reference strains. For active compounds, this determination was also made for MDR strains. In the range of concentrations tested, none of the compounds was active against Gram-negative bacteria, and none of 5–21 (Fig. 1), 28, 29, and 32 was active against any of the tested strains (see Tables S3 and S4 of ESI† for details on remaining strains). The results of the antibacterial activity on Gram-positive strains regarding all other compounds are presented in Table 2. None of the halogenated derivatives exhibited antibacterial activity, similar to that described for the natural product neofiscalin A (2). Regarding the antimicrobial activity against Gram-positive bacteria, 22, 23, and 24 displayed an inhibitory effect on *Enterococcus faecalis* ATCC 29212 and *Staphylococcus aureus* ATCC 29213, whereas 25, 26, 27, 30, and 31 only showed an inhibitory effect on *S. aureus*. The most effective compounds against *S. aureus* reference strain were 26 and 27, with MIC values of 4 μg mL^−1^.

**Table 2:** Antibacterial activity of quinazolinones 22–27, 30, 31 on Gram-positive reference and clinically relevant strains (μg mL^−1^)

Compound	*S. aureus* ATCC 29213		*S. aureus* 66/1 (MRSA)		*E. faecalis* ATCC 29212		*E. faecalis* B3/101 (VRE)	
	MIC	MBC	MIC	MBC	MIC	MBC	MIC	MBC
Neofiscalin^32^	**8**	16	8	32	**8**	32	8	32
5–21	>64	>64	>64	>64	>64	>64	>64	>64
22	**32**	>64	>64	>64	**64**	>64	>64	>64
(−)-22	>64	>64	>64	>64	>64	>64	>64	>64
(+)-22	>64	>64	>64	>64	>64	>64	>64	>64
23	**32**	>64	>64	>64	**32**	>64	>64	>64
23b	>64	>64	>64	>64	>64	>64	>64	>64
24	**16**	>64	>64	>64	**32**	>64	>64	>64
25	**16**	>64	>64	>64	>64	>64	>64	>64
26	**4**	>64	**8**	>64	>64	>64	>64	>64
(−)-26	**4**	>64	**4**	>64	>64	>64	>64	>64
(+)-26	>64	>64	>64	>64	>64	>64	>64	>64
27	**4**	>64	**8**	>64	>64	>64	>64	>64
28	>64	>64	>64	>64	>64	>64	>64	>64
29	>64	>64	>64	>64	>64	>64	>64	>64
30	**16**	>64	>64	>64	>64	>64	>64	>64
31	**16**	>64	>64	>64	>64	>64	>64	>64
32	>64	>64	>64	>64	>64	>64	>64	>64

All the tested compounds exhibited a bacteriostatic activity, with minimal bactericidal concentrations (MBC) greater than 64 μg mL^−1^ (Table 2). Compound 24 was the most effective, with MIC values of 32 μg mL^−1^ and 16 μg mL^−1^ against *E. faecalis* ATCC 29212 and *S. aureus* ATCC 29213, respectively. In the range of concentrations tested, all these compounds were ineffective against *E. faecalis* B3/101, a VRE strain that was also resistant to ampicillin (Table 2). Regarding *S. aureus*, 26 and 27 inhibited the growth of methicillin-resistant *S. aureus* (MRSA) strain 66/1, with MIC values of 8 μg mL^−1^.

Synergistic effects with vancomycin and oxacillin were evaluated for MDR strains, but no effect was observed. These antibiotics are relevant in the treatment of infections caused by *Enterococcus* spp. and *Staphylococcus aureus*, respectively. The compounds showed activity only for Gram-positive strains and, overall, this activity was greater for reference strains than for clinically relevant strains, whether MDR or not. Regarding Gram-positive strains, the range was not equal for all compounds, with a greater number of compounds being active against *S. aureus* than *E. faecalis*. As for *E. faecalis* there appeared to exist an inverse relationship between the compound activity and a resistance against clinically important antibiotics; however, there was not a clear tendency for *S. aureus*. Noteworthy, the first series of compounds showed no relevant effect in the growth of non-malignant cells.

The ability to prevent biofilm formation was evaluated for compounds with antibacterial activity. Concentrations ranging from 2 × MIC to ¼ MIC were tested against *S. aureus* ATCC 29213, *S. aureus* 66/1 and *E. faecalis* ATCC 29212. The highest concentration tested was 64 μg mL^−1^, in order to keep a final in-test concentration of DMSO below 1%. The results were interpreted using a comparative classification that classifies adherence capabilities of tested strains into four categories: (i) non-adherent, (ii) weakly adherent, (iii) moderately adherent and, (iv) strongly adherent.^42^ Optical density cut-off values (ODc) for each microtiter plate were defined as three standard deviations above the mean OD of the negative control. The classification criteria are summarized in Table 3. This classification which uses the negative control as a starting point, instead of using the positive control as reference, reduces the risk of inconsistencies due to external factors that influence biofilm production.^43^ The compounds tested did not inhibit biofilm formation of *E. faecalis* ATCC 29212 (data not shown). *S. aureus* ATCC 29213 was classified as strong biofilm producer, and (−)-26 (2 × MIC, 8 μg mL^−1^) was able to impair this ability (Table 4), while 22–27, 30 and 31 were not able to prevent the formation of a strong biofilm (data not shown). The biofilm forming ability of *S. aureus* 66/1, which was also classified as a strong biofilm producer, was impaired by 26 (MIC, 8 μg mL^−1^ and 2 × MIC, 16 μg mL^−1^), (−)-26 (2 × MIC, 8 μg mL^−1^) and 27 (2 × MIC, 16 μg mL^−1^) (Table 5).

**Table 3:** Classification criteria of adherence capabilities of tested strains using the crystal-violet assay^a^^42^

Classification	Criteria
Non-adherent	OD less than or equal to ODc
Weakly adherent	OD more than ODc and less than two-fold ODc
Moderately adherent	OD more than two-fold ODc and less than four-fold ODc
Strongly adherent	OD more than four-fold ODc

a OD, optical density; ODc, optical density cut-off value.

**Table 4:** Classification of the ability of *S. aureus* ATCC 29213 to adhere and form biofilm after exposure to (−)-26, in comparison with untreated control^a^

Comp.	Concentration (μg mL^−1^)	OD ± SD	Classification
None	0	3.020 ± 0.046	Strong
(−)-26	8 (2 × MIC)	0.347 ± 0.177	Moderate
(−)-26	4 (MIC)	2.231 ± 0.952	Strong
(−)-26	2 (½ MIC)	3.132 ± 0.059	Strong
(−)-26	1 (¼ MIC)	3.119 ± 0.046	Strong

a OD, optical density; SD, standard deviation; ODc, optical density cut-off value. The classification is based on the criteria from Table 3. Average OD value for negative control was 0.082 ± 0.006, therefore ODc equals 0.082 + (3 × 0.006) = 0.100; 2 × ODc = 0.201; 4 × ODc = 0.402.

**Table 5:** Classification of the ability of *S. aureus* 66/1 to adhere and form biofilm after exposure to 26, (−)-26, 27, in comparison with untreated control^a^

Compound	Concentration (μg mL^−1^)	OD ± SD	Classification
None	0	2.943 ± 0.098	Strong
26	16 (2 × MIC)	0.112 ± 0.020	Weak
26	8 (MIC)	0.131 ± 0.045	Weak
26	4 (½ MIC)	0.453 ± 0.248	Strong
26	2 (¼ MIC)	2.980 ± 0.549	Strong
(−)-26	8 (2 × MIC)	0.093 ± 0.013	None
(−)-26	4 (MIC)	0.884 ± 0.439	Strong
(−)-26	2 (½ MIC)	2.952 ± 0.437	Strong
(−)-26	1 (¼ MIC)	3.110 ± 0.210	Strong
27	16 (2 × MIC)	0.285 ± 0.079	Moderate
27	8 (MIC)	2.753 ± 0.676	Strong
27	4 (½ MIC)	3.165 ± 0.192	Strong
27	2 (¼ MIC)	2.755 ± 0.548	Strong

a OD, optical density; SD, standard deviation; ODc, optical density cut-off value. The classification is based on the criteria from Table 3. Average OD value for negative control was 0.078 ± 0.007, therefore ODc equals 0.078 + (3 × 0.007) = 0.098; 2 × ODc = 0.196; 4 × ODc = 0.391.

#### Time–kill kinetics

**Fig. 2:**
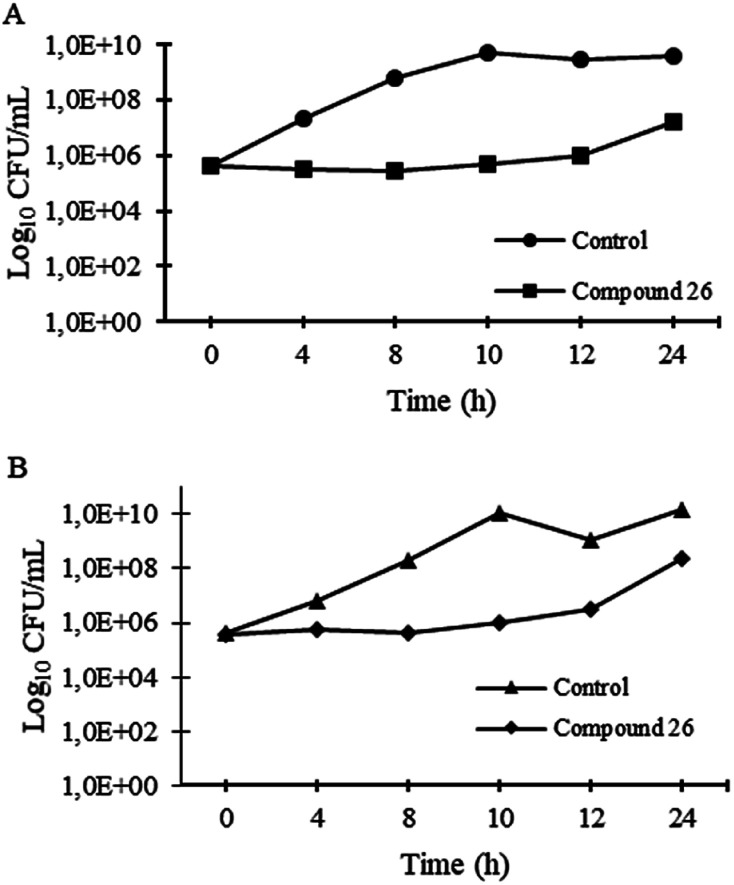
Time-kill kinetics of 26 at 64 μg mL^−1^ and untreated control for *S. aureus* ATCC 29213 (A) and *S. aureus* 66/1 (B) after 20 h incubation.

Even though the quinazolinones with antimicrobial activity described herein have bacteriostatic activity, given a potent activity of 26 against *S. aureus* strains, the time–kill kinetics of this compound was evaluated in order to ascertain its effect over a 24 h incubation. In the presence of 64 μg mL^−1^ of 26 (the highest concentration within the 1% DMSO threshold), it was possible to observe a rise in bacterial growth after 12 h of incubation whereas, for the control, the growth was evident from the start of the assay. In addition, at the final time point there was a 10^2^ CFU mL^−1^ difference between the two conditions, as observed in Fig. 2.

#### Scanning electron microscopy (SEM)

**Fig. 3:**
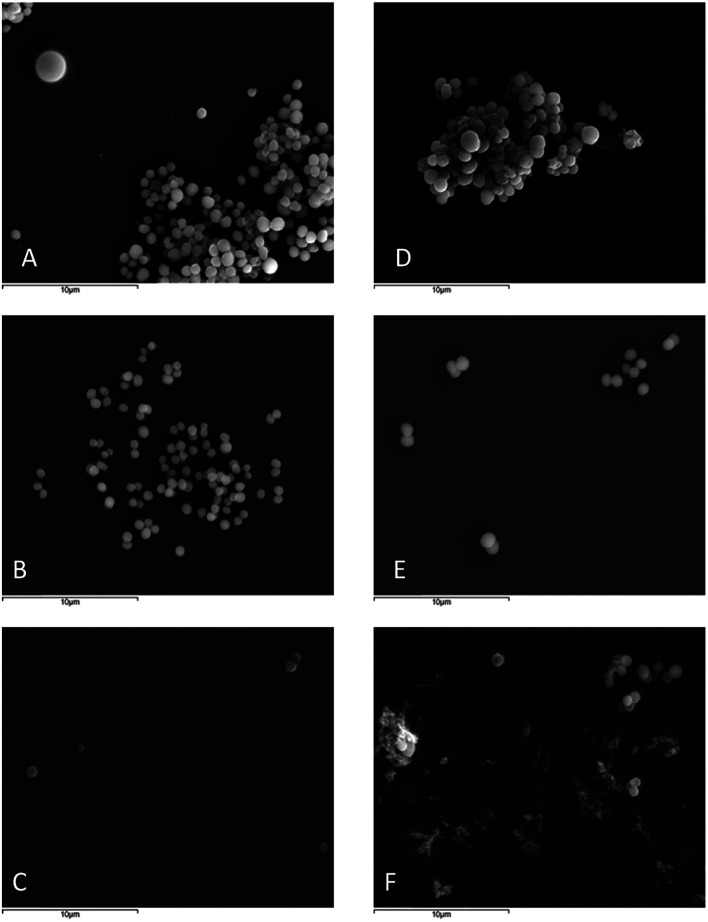
SEM of *S. aureus* ATCC 29213 untreated (A), treated with ½ MIC (2 μg mL^−1^) (B) and MIC (4 μg mL^−1^) (C) of 26; and *S. aureus* 66/1 untreated (D), treated with ½ MIC (4 μg mL^−1^) (E) and MIC (8 μg mL^−1^) (F) of 26 at 5000× amplification.

In order to evaluate a possible effect of 26 on *S. aureus* cell surface and morphology as well as cellular arrangement, SEM analysis was performed for *S. aureus* ATCC 29213 and *S. aureus* 66/1 (Fig. 3). The surfaces of cells treated with 26 at MIC and ½ MIC (Fig. 3B, C, E and F) were relatively smooth and regular, similar to those of the untreated control (Fig. 3A and D). No shrinkage compatible with permeabilization of the membrane and leakage of cytoplasmic material was observed. However, the irregular clusters predominantly observed in the untreated control, an arrangement known to occur in *S. aureus*,^44^ were not present in treated samples, being replaced by short chains, pairs and single cells, with a more uniform size. This was also observed by Gram staining (data not shown).

#### Antifungal activity

The antifungal activity of the test compounds was evaluated against *Candida albicans*, *Aspergillus fumigatus* and *Trichophyton rubrum* by determining MICs.^45,46^ None of the compounds tested showed activity against *C. albicans* and *A. fumigatus* strains. Nevertheless, 26, 28, and 29 exhibited a weak inhibitory effect on a dermatophyte strain (*T. rubrum* FF5) with a MIC value of 128 μg mL^−1^ and a minimal fungicidal concentration (MFC) higher than 128 μg mL^−1^, suggesting that this compound has a fungistatic activity (for details, see Table S5, ESI†). Given these results, 26, 28, and 29 were tested against two additional dermatophyte strains, *i.e. Microsporum canis* FF1 and *Epidermophyton floccosum* FF9; however, no activity was observed. Compounds 26, 28, and 29 were also evaluated for synergistic effects for *T. rubrum*. A synergistic effect was observed for 28 and 29 with fluconazole. Fractional inhibitory concentrations index (FICI), determined by checkerboard method, was < to 0.5 (0.06 for 28 and 0.13 for 29).

In order to evaluate *in vitro* enantioselectivity activities, including antibacterial and antifungal activities, the most promising derivatives 22, 23, and 26 were obtained in milligram scale by a semi-preparative enantioselective liquid chromatography, employing a tris-3,5-dimethylphenylcarbamate amylose column with multiple injection in a 200 μL loop (see ESI† for details).

The pure enantiomers of 22, 23, and 26 were evaluated for antibacterial and antifungal activities. The enantiomer (−)-26 showed a MIC value of 4 μg mL^−1^ for reference strain *S. aureus* ATCC 29213, sensitive clinical isolate *S. aureus* 40/61/24, and methicillin-resistant strain *S. aureus* 66/1, while the enantiomer (+)-26 showed no effect (Table 2). Noteworthy, these derivatives showed higher potency than the natural product neofiscalin A (2), (tested by the same group with the same conditions).^32,47,48^ None of the pure enantiomers was active against the fungi tested.

#### Structure–activity relationship (SAR) study

**Fig. 4:**
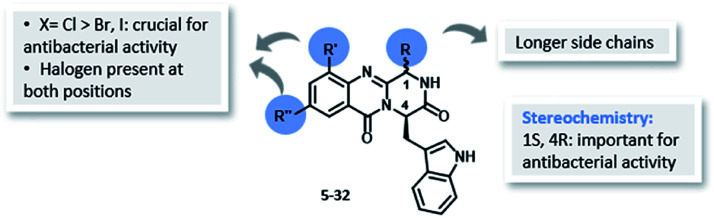
Structure–activity relationship for antibacterial activity of the library of quinazolinones 5–32.

Regarding antibacterial activity, SAR (Fig. 4) suggested that the presence of a halogen atom at positions C-9 and/or C-11 plays a crucial role for this activity since all the non-halogenated derivatives were inactive against all the tested strains (for details see Tables S3 and S4, ESI†). In fact, compounds containing chlorine at one or both positions exhibited better antibacterial activity compared to those having bromine and iodine. Higher antibacterial activity was obtained when the halogen atom is present at C-9 and C-11 positions (*i.e.*, 25, 26, 27 and 32) and/or the presence of longer side chains at C-1. Compounds 26, 28, and 29 were also evaluated for their synergistic effects on *T. rubrum*. A synergistic effect was observed for 28 and 29 with fluconazole (data not shown). The enantiopure (−)-26 showed significant antibacterial effect against a resistant strain of *S. aureus* whereas its antipode [(+)-26] did not. This result emphasizes that the configuration (1*S*,4*R*) is crucial for the antibacterial activity of quinazolinone scaffold.

**Fig. 5:**
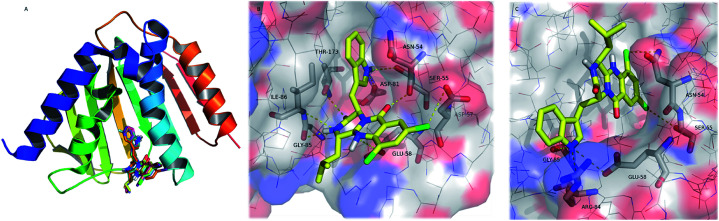
(A) Ribbon representation of GyrB (pdb code 3u2d), and docked 22–32 (sticks). (B and C) Detailed view of two representative top docked poses of 26 (yellow sticks) with polar interactions depicted as yellow broken lines (residues evolved are labelled); GyrB is represented as transparent surface, with carbons, oxygens, and nitrogens coloured grey, red, and blue, respectively. Hydrogens are omitted for simplification.

To understand the structural basis of the potential ligand binding to GyrB, and Fts, each prepared ligand was individually docked with the enzymes at their active site using AutodockVina. Test molecules present free binding energies on GyrB ranked from −9.3 to −9.6 kcal.mol^−1^ (Table 6), and all of the test compounds are predicted to establish more stable complexes with GyrB (lower docking score than test compound 08B; −7.8 kcal.mol^−1^). All the test compounds are predicted to have lower affinity to FtsZ than test compound 9PC (docking scores higher than −10.1 kcal.mol^−1^, respectively). Therefore, as GyrB has already been reported as a target for other quinazoline derivatives^49,50^ it is hypothesized as a possible target for the tested antimicrobial pyrazinoquinazolines. However, further target-specific testing will be performed in the near future to counterproof the *in silico* findings. s Spatial conformation of test compounds and key interactions of 26 in the active site of GyrB are presented in Fig. 5.

**Table 6:** Docking scores of test molecules (22–32) and controls (08B, and 9PC) using GyrB, and FtsZ as targets^a^

Test molecules	Docking scores (kcal mol^−1^)	
	3u2d (GyrB)	4dxd (FtsZ)
22	−9.5	−9.5
23	−9.6	−9.5
24	−9.3	−8.5
25	−9.6	−7.8
26	−9.6	−9.7
27	−9.5	−6.9
28	−9.3	−8.4
29	−9.5	−9.1
30	−9.5	−7.7
31	−9.4	−9.1
32	−9.4	−8.1
08B	−7.8	—
9PC	—	−10.1

a 08B (3u2d co-crystallized 4-bromo-5-methyl-*N*-[1-(3-nitropyridin-2-yl)piperidin-4-yl]-1*H*-pyrrole-2-carboxamide), and 9PC (4dxd co-crystallized 3-[(6-chloro[1,3]thiazolo[5,4-*b*]pyridin-2-yl)methoxy]-2,6-difluorobenzamide).

The pyrazinoquinazoline moiety binds to the ATP binding site of GyrB, participating in hydrogen interactions with Ser-55, Glu-58, Gly-85, Ile-86 and Thr-173, and halogen interaction with Asp-57whereas the indole group is involved in hydrogen interactions with Asn-54 and Asp-81 (Fig. 5B). Alternatively, another docking pose suggests hydrogen interactions with Glu-58, Arg-84, and Gly-85, as well as halogen interactions with Asn-54 and Ser-55 (Fig. 5C). Some of these residues have already been described as being involved in the binding of known inhibitors to GyrB^51–54^ In both docking poses (Fig. 5B and C), the halogen atoms are predicted as being involved in the interaction with GyrB. In fact, currently, the halogenation of compounds has become an important strategy in drug design.^55^

Halogens are found to increase membrane permeability and the *t*_1/2_ by lowering metabolic degradation, and enhance binding affinity to targets.^56,57^ Halogens, especially chlorine and bromine, are present in a significant number of drugs, contributing favourably to ligand–protein interaction.^58^ Halogen bonding is a non-covalent interaction similar to the hydrogen bond (halogens act as hydrogen bond acceptors), and characterized by its directionality.^59^ There are several physico–chemical properties of chloride that may justify its more favourable contribution to activity when compared to bromide and iodide. In fact, chloride is more electronegative and it is a smaller atom, forming halogen bonds with specific lengths and angles, which contribute to a more appropriate fit to the target stereoelectronic profile. Furthermore, the presence of halogens has already been described as being responsible for the increase in the affinity of known inhibitors to the GyrB ATP binding site.^60,61^

However, molecular docking using a rigid target may be insufficient for the determination of the structure and the stability of the ligand:target complex. To gain a detailed insight in the energetic and geometric behaviour of the 26:GyrB complex in aqueous environment, a 2 ns MD simulation was performed based on two most stable complex structures obtained from the docking study considering the effects of the target flexibility and the explicit water solvation.

**Fig. 6:**
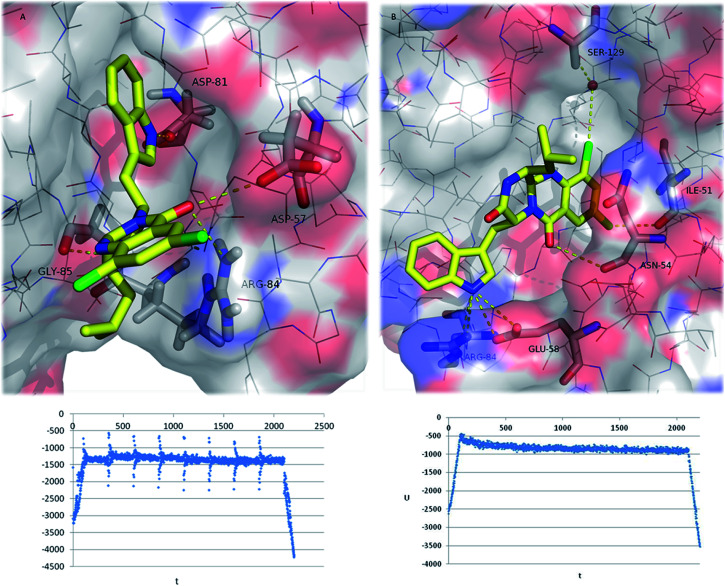
Final (2200 ps) MD conformations of 26:GyrB complexes (top ranked docking poses (A) and (B)). Polar interactions are depicted as yellow broken lines (residues evolved are labelled); GyrB is represented as transparent surface, with carbons, oxygens, and nitrogens coloured grey, red, and blue, respectively. Hydrogen atoms are omitted for simplification. Potential energy plot of the complexes during MD simulation is represented below each MD image (*U* = potential energy; *t* = time).

The final MD conformation of 26:GyrB complexes and potential energy plots of ligand conformations along the simulation time (Fig. 6) were obtained. When MD was performed on the 26's top docked conformation (Fig. 6A), the obtained result after 2200 ns simulation shows some differences from the original ligand conformation, namely the loss of the halogen interaction with Asp-57 (instead, the halogen binds to outside water molecules-not shown for simplification). Another high-ranked docking pose of 26 (with the chlorides inside the pocket) (Fig. 6B) was subjected to MD, showing that halogen interactions are established to Ile-51 and to Ser-129 *via* a water molecule; and hydrogen bonds are formed between 26 and Asn-54, Glu-58, and Arg-84.^62^ In conclusion, the halogenation of pyrazinoquinazolines was a successful approach in drug design of new antimicrobial compounds, potentially increasing the binding affinity and binding selectivity to GyrB.

#### Cytotoxicity of compound 26

**Fig. 7:**
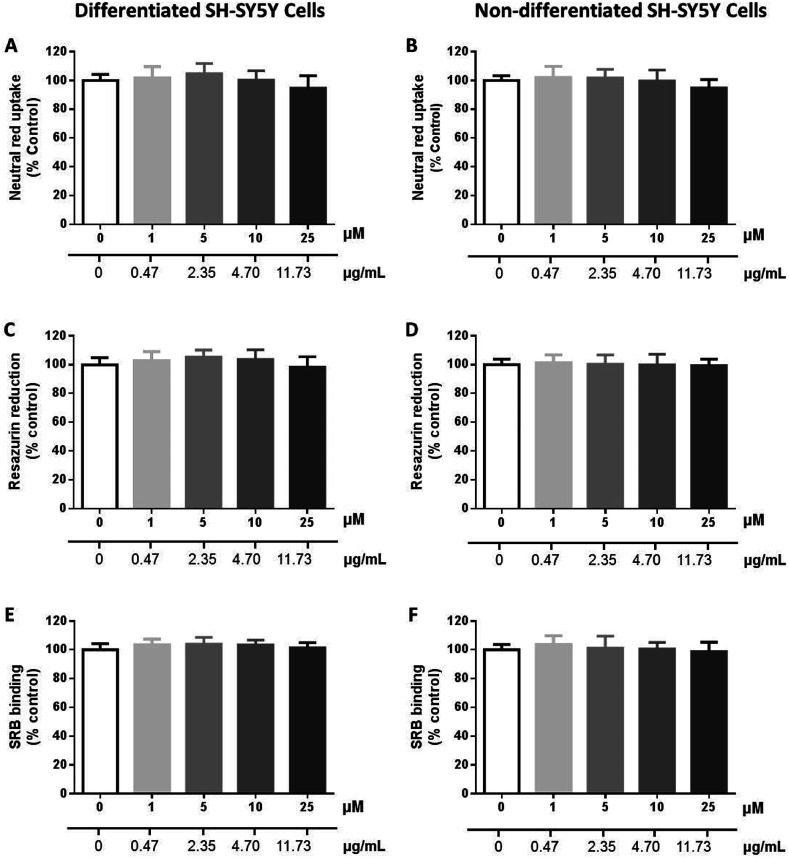
Cytotoxicity of 26 (0–25 μM) in differentiated (A, C and E) and non-differentiated (B, D and F) SH-SY5Y cells evaluated by the neutral red (NR) uptake (A and B), resazurin (REZ) reduction (C and D) and sulforhodamine B (SRB) binding (E and F) assays, 24 h after exposure. Results are expressed as mean ± SD from at least 3 independent experiments, performed in triplicate. Statistical comparisons were made using the parametric method of one-way ANOVA, followed by the Dunnett's multiple comparisons test.

The cytotoxicity of 26 (0–25 μM) was evaluated in differentiated and non-differentiated SH-SY5Y cells, by the neutral red (NR) uptake, resazurin (REZ) reduction and sulforhodamine B (SRB) binding assays, 24 h after exposure. As observed in Fig. 7, no significant cytotoxicity towards both differentiated and non-differentiated SH-SY5Y cells was observed, as evaluated by the results obtained for the three adopted *in vitro* cytotoxicity assays.

## Conclusion

Among a new series of indolomethyl pyrazino[1,2-*b*]quinazoline-3,6-diones, 26 and 27 exhibited a potent antibacterial activity against *S. aureus* strains, with MIC values of 4 μg mL^−1^ for a reference strain and 8 μg mL^−1^ for a methicillin-resistant strain (*S. aureus* 66/1). Comparing with the marine natural product neofiscalin A (2), a two-fold reduction in the MIC values was observed. Regarding the structural complexity and synthetic pathways, the excellent and inspiring results obtained in the present study show that simpler molecules than neofiscalin A (2) are quite promising to find new agents to overcome MDR strains. *In silico* docking and molecular dynamics revealed that halogenation was a successful design strategy to discover new indolymethyl pyrazinoquinazoline with antimicrobial activity. Although the investigated biological activities of fiscalins are still in preliminary stages, there is no doubt that these are privileged structures with potential as drug candidates. Nevertheless, mainly due to the limited amounts isolated, most of the studies correspond to phenotypic screening assays and little is known about their molecular targets and pharmacokinetic properties. One limitation of the one-pot synthesis is related to the low yields obtained, which can be a drawback on the scale up of these compounds. The main challenge in synthesis is the intricate stereochemistry found in the more complex structures. Although the three-component one-pot assembly of this scaffold is a very efficient procedure to obtain some of these secondary metabolites, the high temperatures involved resulted in partial epimerization. Differentiation between stereoisomers was noted in their antimicrobial action but also occurs in drug disposition and is of particular significance for those processes that depend upon a direct interaction between the drug and a chiral biological macromolecule, *e.g.*, active transport processes, binding to plasma and tissue proteins, and drug metabolism, deserving this issue special attention in future work.

## Experimental

### Materials and methods

#### General procedure

All reagents were analytical grade. Dried pyridine and triphenylphosphite were purchased from Sigma (Sigma-Aldrich Co. Ltd, Gillingham, UK). Anthranilic acid (33) and protected amino acids 34 and 36 were purchased from TCI (Tokyo Chemical Industry Co. Ltd, Chuo-ku, Tokyo, Japan). Column chromatography purifications were performed using flash silica Merck 60, 230–400 mesh (EMD Millipore corporation, Billerica, MA, USA) and preparative TLC was carried out on pre-coated plates Merck Kieselgel 60 F_254_ (EMD Millipore corporation, Billerica, MA, USA), spots were visualized under UV light (Vilber Lourmat, Marne-la-Vallée, France). Melting points were measured in a Köfler microscope and are uncorrected. Infrared spectra were recorded in a KBr microplate in a FTIR spectrometer Nicolet iS10 from Thermo Scientific (Waltham, MA, USA) with Smart OMNI-Transmission accessory (Software 188 OMNIC 8.3). ^1^H and ^13^C NMR spectra were recorded in CDCl_3_ (Deutero GmbH, Kastellaun, Germany) at room temperature unless otherwise mentioned on Bruker AMC instrument (Bruker Biosciences Corporation, Billerica, MA, USA), operating at 300 MHz for ^1^H and 75 MHz for ^13^C. Carbons were assigned according to DEPT, HSQC and/or HMBC experiments. Optical rotation was measured at 25 °C using the ADP 410 polarimeter (Bellingham + Stanley Ltd, Tunbridge Wells, Kent, UK), using the emission wavelength of sodium lamp, concentrations are given in g per 100 mL. High resolution mass spectra (HRMS) were measured on a Bruker FTMS APEX III mass spectrometer (Bruker Corporation, Billerica, MA, USA) recorded as ESI (Electrospray) made in Centro de Apoio Científico e Tecnolόxico á Investigation (CACTI, University of Vigo, Pontevedra, Spain). The purity of synthesized compounds was determined by reversed-phase LC with diode array detector (DAD) using C18 column (Kimetex®, 2.6 EV0 C18 100 Å, 250 × 4.6 mm), the mobile phase was MeOH : H_2_O (50 : 50), and the flow rate was 1.0 mL min^−1^. Enantiomeric ratio was determined by enantioselective LC (LCMS-2010EV, Shimadzu, Lisbon, Portugal), employing a system equipped with a chiral column (Lux® 5 μm Amylose-1, 250 × 4.6 mm) and UV-detection at 254 nm, mobile phase was hexane : EtOH (90 : 10) and the flow rate was 0.5 mL min^−1^. For semi-preparative chromatography, a HLPC system consisted of a Shimadzu LC-6AD pump with a 200 μL loop was used with an amylose tris-3,5-dimethylphenylcarbamate coated with Nucleosil (500 A, 7 m, 20%, w/w) packed into a stainless steel (200 mm × 7 mm I.D. size) column, prepared in the UFSCar laboratory.^63^ Reagents used in cell culture, including Dulbecco's modified Eagle's medium (DMEM) high glucose, sodium bicarbonate, trypsin–ethylenediamine tetraacetic acid (EDTA) solution (0.25% trypsin/1 mM EDTA), retinoic acid (RA), 12-*O*-tetradecanoylphorbol-13-acetate (TPA), Trizma® base, neutral red (NR) solution, sulforhodamine B (SRB) and resazurin (REZ) were obtained from Sigma-Aldrich (Germany). Antibiotic mixture (10 000 U mL^−1^ penicillin, 10 000 μg mL^−1^ streptomycin) was obtained from Biochrom (Germany). Triton™ X-100 detergent solution was acquired from Thermo Fisher Scientific (Waltham, MA, USA). Heat inactivated fetal bovine serum (FBS), Hanks' balanced salt solution (HBSS) with or without calcium and magnesium [HBSS (+/+) or HBSS (−/−), respectively], and phosphate buffer solution with or without calcium and magnesium [PBS (+/+) or PBS (−/−), respectively] were obtained from Gibco (United Kingdom).

#### General conditions for the synthesis of 22–32

In a closed vial, 5-chloro anthranilic acid, 33a, 34 mg, 200 μmol for 22, 23, and 24, or 3,5-dichloroanthranilic acid, (**33b**, 41 mg, 200 μmol) for 25, 26, and 27, or 5-iodoanthranilic acid (33c, 53 mg, 200 μmol), for 28 and 30, or 5-bromoanthranilic acid (33d, 43 mg, 200 μmol), for 29 and 31, or 3,5-diodoanthranilic acid (33e, 78 mg, 200 μmol) for 32; was added *N*-Boc-l-valine (34a, 44 mg, 200 μmol) for 22, 25, 28, and 29, or *N*-Boc-l-leucine (34c, 46 mg, 200 μmol) for 23, 26, 30, and 32, or *N*-Boc-l-isoleucine (34d, 46 mg, 200 μmol) for 24 and 27 (as presented in Table 1), and triphenylphosphite (63 μL, 220 μmol) were added along with 1 mL of dried pyridine. The vial was heated in a heating block with stirring at 55 °C for 16–24 h. After cooling the mixture to room temperature, d-tryptophan methyl ester hydrochloride (36, 51 mg, 200 μmol) was added, and the mixture was irradiated in the microwave at a constant temperature at 220 °C for 1.5 min. Four reaction mixtures were prepared in the same conditions and treated in parallel. After removing the solvent with toluene, the crude product was purified by flash column chromatography using hexane : EtOAc (60 : 40) as a mobile phase. The preparative TLC was performed using DCM : Me_2_CO (95 : 5) as mobile phase. The major compound appeared as a black spot with no fluorescence under the UV light. The desired compounds were collected as yellow solids. Before analysis, compounds were recrystallized from MeOH.

#### (1*S*,4*R*)-4-((1*H*-indol-3-yl)methyl)-8-chloro-1-isopropyl-1,2-dihydro-6*H*-pyrazino[2,1-*b*]quinazoline-3,6(4*H*)-dione (22)

Yield: 39.8 mg, 7%; e.r = 56 : 44; mp: 200.3–202.4 °C; [*α*]_D_^30^ = −273 (*c* 0.05; CHCl_3_); *ν*_max_ (KBr) 3277, 2924, 1682, 1592, 1470, 1323, 741 cm^−1^; ^1^H NMR (300 MHz, CDCl_3_): *δ*_H_ 8. 33 (d, 1H, *J* = 2.5 Hz, H-8), 8.33 (br, 1H, H-21), 7.70 (dd, 1H, *J* = 8.7, 2.5 Hz, H-10), 7.50 (d, *J* = 8.7 Hz, H-11), 7.39 (d, 1H, *J* = 8.0 Hz, H-26), 7.30 (d, *J* = 8.1 Hz, H-23), 7.12 (t, 1H, *J* = 8.0 Hz, H-24), 6.92 (t, 1H, *J* = 8.0 Hz, H-25), 6.63 (d, 1H, *J* = 2.3 Hz, H-20), 5.64 (dd, 1H, *J* = 5.4,2.7, H-4), 5.72 (s, 1H, H-2), 3.73 (dd, 1H, *J* = 15.0, 2.7 Hz, H-18a), 3.63 (dd, 1H, *J* = 15.0, 5.4 Hz, H-18b), 2.76 (d, *J* = 2.3 Hz, H-1), 2.60 (ddd, 1H, *J* = 13.9, 6.9, 2.3 Hz, H-15), 0.64 (d, 6H, *J* = 6.1 Hz, H-16 and H-17); ^13^C NMR (75 MHz, CDCl_3_): *δ*_C_169.2 (C-3), 159.9 (C-6), 150.6 (C-14), 145.6 (C-12), 136.1 (C-22), 135.7 (C-10), 132.8 (C-9), 128.9 (C-8), 127.2 (C-27), 126.2 (C-11), 123.6 (C-20), 122.5 (C-24), 121.2 (C-7), 119.9 (C-25), 118.6 (C-26), 111.1 (C-23), 109.1 (C-19), 57.0 (C-4), 58.0 (C-1), 29.3 (C-16), 27.3 (C-18), 18.8 (C-16), 14.8 (C-17); (+)-HRMS-ESI *m*/*z*: 421.1442 (M + H)^+^, 443.1264 (M + Na)^+^ (calculated for C_23_H_22_N_4_O_2_Cl, 421.1432; C_23_H_21_N_4_O_2_ClNa, 443.1252).

#### (1*S*,4*R*)-4-((1*H*-indol-3-yl)methyl)-8-chloro-1-isobutyl-1,2-dihydro-6*H*-pyrazino[2,1-*b*]quinazoline-3,6(4*H*)-dione (23)

Yield: 12.3 mg, 3%; e.r = 44 : 56; mp: 208.8–210.1 °C; [*α*]_D_^30^ = +154 (*c* 0.15; CHCl_3_); *ν*_max_ (KBr) 3277, 2924, 1682, 1592, 1470, 1323, 741 cm^−1^; ^1^H NMR (300 MHz, CDCl_3_): *δ* 8. 33 (d, 1H, *J* 2.4 Hz, H-8), 8.07 (br, 1H, H-21), 7.70 (dd, 1H, *J* = 8.7, 2.4 Hz, H-10), 7.54 (d, *J* = 8.7 Hz, H-11), 7.46 (d, 1H, *J* = 7.8 Hz, H-26), 7.29 (d, *J* = 7.8 Hz, H-23), 7.13 (t, 1H, *J* = 7.8 Hz, H-24), 6.98 (t, 1H, *J* = 7.8 Hz, H-25), 6.65 (d, 1H, *J* = 2.4 Hz, H-20), 5.65 (dd, 1H, *J* = 5.3, 2.7, H-4), 5.71 (s, 1H, H-2), 3.76 (dd, 1H, *J* = 15.1,2.7 Hz, H-18a), 3.63 (dd, 1H, *J* = 15.1, 5.3 Hz, H-18b), 2.70 (dd, *J* = 9.7, 2.3 Hz, H-1), 1.97 (ddd, 1H, *J* = 11.8, 7.7, 2.1 Hz, H-16), 1.39–1.30 (m, 2H, H-15), 0.77 (d, 3H, *J* = 6.4 Hz, H-17a), 0.28 (d, 3H, *J* = 6.5 Hz, H-17b); ^13^C NMR (75 MHz, CDCl_3_): *δ* 169.1 (C-3), 159.8 (C-6), 151.9 (C-14), 145.5 (C-12), 136.0 (C-22), 135.1 (C-10), 132.9 (C-9), 129.1 (C-8), 127.2 (C-27), 126.2 (C-11), 123.6 (C-20), 122.7 (C-24), 121.2 (C-7), 120.2 (C-25), 118.7 (C-26), 111.1 (C-23), 109.5 (C-19), 57.5 (C-4), 50.8 (C-1), 40.2 (C-15), 27.2 (C-18), 24.1 (C-16), 23.3 (C-17a), 19.7 (C-17b); (+)-HRMS-ESI *m*/*z*: 435.1579 (M + H)^+^, 457.1206 (M + Na)^+^ (calculated for C_24_H_24_N_4_O_2_Cl, 435.1588; C_24_H_23_N_4_O_2_ClNa, 457.1408).

#### (1*S*,4*R*)-4-((1*H*-indol-3-yl)methyl)-1-((*S*)-*sec*-butyl)-8-chloro-1,2-dihydro-6*H*-pyrazino[2,1-*b*]quinazoline-3,6(4*H*)-dione (24)

Yield: 16.7 mg, 3%; e.r = 46 : 54; mp: 209.1–211.2 °C; [*α*]_D_^30^ = +130 (*c* 0.03; CHCl_3_); *ν*_max_ (KBr) 3277, 2924, 1682, 1592, 1470, 1323, 741 cm^−1^; ^1^H NMR (300 MHz, CDCl_3_): *δ* 8. 33 (d, 1H, *J* 2.4 Hz, H-8), 8.05 (br, 1H, H-21), 7.70 (dd, 1H, *J* = 8.7, 2.4 Hz, H-10), 7.49 (d, *J* = 8.7 Hz, H-26), 7.29 (d, *J* = 8.0 Hz, H-23), 7.13 (t, 1H, *J* = 8.0 Hz, H-24), 6.92 (t, 1H, *J* = 8.0 Hz, H-25), 6.63 (d, 1H, *J* = 2.4 Hz, H-20), 5.64 (dd, 1H, *J* = 5.3, 2.8, H-4), 5.80 (s, 1H, H-2), 3.72 (dd, 1H, *J* = 15.1, 2.8 Hz, H-18a), 3.62 (dd, 1H, *J* = 15.1, 5.3 Hz, H-18b), 2.69 (d, *J* = 2.2 Hz, H-1), 2.29 (ddd, 1H, *J* = 11.6, 7.7, 4.8 Hz, H-15), 0.99–0.79 (m, 2H, H-16), 0.70 (d, 3H, *J* = 7.7 Hz, H-17a), 0.63 (d, 3H, *J* = 7.2 Hz, H-17b); ^13^C NMR (75 MHz, CDCl_3_): *δ* 169.1 (C-3), 159.9 (C-6), 150.7 (C-14), 145.5 (C-12), 136.0 (C-22), 135.1 (C-10), 132.8 (C-9), 128.9 (C-8), 127.2 (C-27), 126.2 (C-11), 123.5 (C-20), 122.7 (C-24), 121.1 (C-7), 120.1 (C-25), 118.6 (C-26), 111.1 (C-23), 109.2 (C-19), 58.3 (C-1), 57.0 (C-4), 36.2 (C-15), 27.3 (C-18), 23.1 (C-16), 15.6 (C-17a), 12.0 (C-17b); (+)-HRMS-ESI *m*/*z*: 435.1580 (M + H)^+^, 457.1394 (M + Na)^+^ (calculated for C_24_H_24_N_4_O_2_Cl, 434.1588; C_24_H_23_N_4_O_2_ClNa, 457.1408).

#### (1*S*,4*R*)-4-((1*H*-indol-3-yl)methyl)-8,10-dichloro-1-isopropyl-1,2-dihydro-6*H*-pyrazino[2,1-*b*]quinazoline-3,6(4*H*)-dione (25)

Yield: 22.1 mg, 5%; e.r = 43 : 57; mp: 232.9–235.1 °C; [*α*]_D_^30^ = +140 (*c* 0.038; CHCl_3_); *ν*_max_ (KBr) 3293, 2954, 1671, 1611, 1511, 1465, 1240, 772, and 697 cm^−1^; ^1^H NMR (300 MHz, DMSO-d_6_): *δ* 10.2 (br, 1H, H-21), 8. 20 (d, 1H, *J* 2.4 Hz, H-8), 7.83 (d, 1H, *J* = 2.4 Hz, H-10), 7.37 (d, 1H, *J* = 8.1 Hz, H-26), 7.33 (d, *J* = 8.1 Hz, H-23), 7.11 (s, 1H, H-2), 7.07 (t, 1H, *J* = 7.6 Hz, H-24), 6.87 (t, 1H, *J* = 7.6 Hz, H-25), 6.66 (d, 1H, *J* = 2.3 Hz, H-20), 5.50 (dd, 1H, *J* = 5.3, 2.9, H-4), 3.69 (dd, 1H, *J* = 14.9, 2.9 Hz, H-18a), 3.58 (dd, 1H, *J* = 14.9, 5.3 Hz, H-18b), 2.76 (d, *J* = 2.2 Hz, H-1), 2.60–254 (m, 1H, H-15), 0.71 (dd, 6H, *J* = 8.4, 7.2 Hz, H-16, H-17); ^13^C NMR (75 MHz, CDCl_3_): *δ* 169.2 (C-3), 159.9 (C-6), 150.6 (C-14), 145.7 (C-12), 136.0 (C-22), 135.1 (C-10), 132.8 (C-9), 128.9 (C-8), 127.2 (C-27), 126.2 (C-11), 123.6 (C-20), 122.7 (C-24), 121.2 (C-7), 120.1 (C-25), 118.6 (C-26), 111.1 (C-23), 109.2 (C-19), 58.1 (C-1), 57.0 (C-4), 29.3 (C-15), 27.3 (C-18), 18.8 (C-16), 14.8 (C-17); (+)-HRMS-ESI *m*/*z*: 455.1436 (M + H)^+^ (calculated for C_23_H_21_N_4_O_2_Cl_2_, 455.1041).

#### (1*S*,4*R*)-4-((1*H*-indol-3-yl)methyl)-8,10-dichloro-1-isobutyl-1,2-dihydro-6*H*-pyrazino[2,1-*b*]quinazoline-3,6(4*H*)-dione (26)

Yield: 41.8 mg, 4.5%; e.r = 60 : 40; mp: 253.4–254.3 °C; [*α*]_D_^30^ = −169 (*c* 0.04; CHCl_3_); *v*_max_ (KBr) 3289, 2960, 1680, 1600, 1556, 1315, 757, 720 cm^−1^; ^1^H NMR (300 MHz, DMSO-d_6_): 10.22 (br, 1H, H-21), *δ* 8.13 (d, 1H, *J* 2.4 Hz, H-8), 7.75 (d, 1H, *J* = 2.4 Hz, H-10), 7.33 (d, 1H, *J* = 8.0 Hz, H-26), 7.25 (d, *J* = 8.0 Hz, H-23), 7.19 (br, H-2), 7.00 (t, 1H, *J* = 8.0 Hz, H-24), 6.82 (t, 1H, *J* = 8.0 Hz, H-25), 6.60 (d, 1H, *J* = 2.4 Hz, H-20), 5.42 (dd, 1H, *J* = 5.4, 2.9, H-4), 3.63 (dd, 1H, *J* = 15.0, 2.9 Hz, H-18a), 3.50 (dd, 1H, *J* = 15.0, 5.4 Hz, H-18b), 2.68 (dd, *J* = 7.3, 4.9 Hz, H-1), 1.94–1.86 (m, 1H H-16a), 1.50 (tt, 1H, *J* = 13.2, 6.5 Hz, H-15), 1.29–1.22 (m, 1H, H-16b), 0.56 (d, 3H, *J* = 6.6 Hz, H-17a), 0.35 (d, 3H, *J* = 6.6 Hz, H-17b); ^13^C NMR (75 MHz,DMSO-d_6_): *δ* 168.4 (C-3), 158.8 (C-6), 152.8 (C-14), 142.0 (C-12), 135.9 (C-22), 134.1 (C-10), 132.6 (C-9), 131.4 (C-8), 126.5 (C-27), 124.3 (C-11), 123.5 (C-20), 121.6 (C-24), 121.5 (C-7), 118.9 (C-25), 117.7 (C-26), 111.1 (C-23), 107.7 (C-19), 57.3 (C-4), 50.6 (C-1), 39.6 (C-15), 26.2 (C-18), 23.8 (C-16), 22.1 (C-17a), 20.5 (C-17b); (+)-HRMS-ESI *m*/*z*: 469.1186 (M + H)^+^, 491.1008 (M + Na)^+^ (calculated for C_24_H_23_N_4_O_2_Cl_2_, 469.1198; C_24_H_22_N_4_O_2_Cl_2_Na, 491.1018).

#### (1*S*,4*R*)-4-((1*H*-indol-3-yl)methyl)-1-((*S*)-*sec*-butyl)-8,10-dichloro-1,2-dihydro-6*H*-pyrazino[2,1-*b*]quinazoline-3,6(4*H*)-dione (27)

Yield: 22.4 mg, 2.6%; e.r = 71 : 29; mp: 252.9–254.7 °C; [*α*]_D_^30^ = −264 (*c* 0.034; CHCl_3_); *v*_max_ (KBr) 3373, 3074, 2922, 1698, 1609, 1550, 1450, 1262, 794 cm^−1^; ^1^H NMR (300 MHz, CDCl_3_): *δ* 8. 33 (d, 1H, *J* 2.4 Hz, H-8), 8.05 (br, 1H, H-21), 7.70 (d, 1H, *J* = 2.4 Hz, H-10), 7.38 (d, 1H, *J* = 7.9 Hz, H-26), 7.29 (d, *J* = 7.9 Hz, H-23), 7.13 (t, 1H, *J* = 7.9 Hz, H-24), 6.92 (t, 1H, *J* = 7.9 Hz, H-25), 6.63 (d, 1H, *J* = 2.4 Hz, H-20), 5.64 (dd, 1H, *J* = 5.3, 2.8, H-4), 5.80 (s, 1H, H-2), 3.72 (dd, 1H, *J* = 15.0, 2.8 Hz, H-18a), 3.62 (dd, 1H, *J* = 15.0,5.3 Hz, H-18b), 2.69 (d, *J* = 2.2 Hz, H-1), 2.29 (ddd, 1H, *J* = 11.6, 7.9, 4.8 Hz, H-15), 0.99–0.79 (m, 2H, H-16), 0.70 (d, 3H, *J* = 7.3 Hz, H-17a), 0.63 (d, 3H, *J* = 7.3 Hz, H-17b); ^13^C NMR (75 MHz, CDCl_3_): *δ* 168.9 (C-3), 159.5 (C-6), 151.3 (C-14), 142.5 (C-12), 136.1 (C-22) 135.0 (C-10), 133.2 (C-9), 132.4 (C-8), 127.1 (C-27), 125.1 (C-11), 123.5 (C-20), 122.9 (C-24), 122.1 (C-7), 120.2 (C-25), 118.6 (C-26), 111.1 (C-23), 109.2 (C-19), 58.2 (C-1), 57.3 (C-4), 36.2 (C-15), 27.1 (C-18), 23.6 (C-16), 15.5 (C-17a), 12.1 (C-17b); (+)-HRMS-ESI *m*/*z*: 469.1186 (M + H)^+^, 491.1024 (M + Na)^+^ (calculated for C_24_H_23_N_4_O_2_Cl_2_, 469.1198; C_24_H_22_N_4_O_2_Cl_2_Na, 491.1018).

#### (1*S*,4*R*)-4-((1*H*-indol-3-yl)methyl)-8-iodo-1-isopropyl-1,2-dihydro-6*H*-pyrazino[2,1-*b*]quinazoline-3,6(4*H*)-dione (28)

Yield: 21.2 mg, 4.1%; e.r = 51 : 49; mp: 246.5–248.2 °C; [*α*]_D_^30^ = −175 (*c* 0.041; CHCl_3_); *v*_max_ (KBr) 3311, 3192, 2963, 1681, 1655, 1588, 1464, 1246, 828, and 741 cm^−1^; ^1^H NMR (300 MHz, CDCl_3_): *δ* 8. 71 (d, 1H, *J* 2.4 Hz, H-8), 8.04 (br, 1H, H-21), 8.02 (dd, 1H, *J* = 8.6, 2.1 Hz, C-10), 7.41 (d, 1H, *J* = 8.0 Hz, C-11), 7.30 (d, *J* = 8.4 Hz, H-26), 7.29 (d, *J* = 8.4 Hz, H-23), 7.13 (ddd, 1H, *J* = 8.0, 7.1, 0.9 Hz, H-24), 6.94 (ddd, 1H, *J* = 8.0, 7.1, 0.9 Hz, H-25), 6.61 (d, 1H, *J* = 2.4 Hz, H-20), 5.64 (dd, 1H, *J* = 5.4, 2.8, H-4), 5.67 (s, 1H, H-2), 3.73 (dd, 1H, *J* = 14.9, 2.7 Hz, H-18a), 3.61 (dd, 1H, *J* = 15.1, 5.4 Hz, H-18b), 2.64 (d, *J* = 2.4 Hz, H-1), 2.63–2.56 (m, 1H, H-15), 0.63 (d, 6H, *J* = 6.8 Hz, H-16 and H-17); ^13^C NMR (75 MHz, CDCl_3_): *δ* 169.1 (C-3), 159.5 (C-6), 151.0 (C-14), 146.3 (C-12), 143.5 (C-11), 136.0 (C-22), 135.7 (C-10), 129.0 (C-8), 127.2 (C-27), 123.5 (C-20), 122.7 (C-24), 121.7 (C-7), 120.2 (C-25), 118.7 (C-26), 111.1 (C-23), 109.3 (C-19), 91.4 (C-9), 58.1 (C-1), 57.0 (C-4) 29.7 (C-15), 27.3 (C-18), 18.8 (C-16), 14.8 (C-17); (+)-HRMS-ESI *m*/*z*: 513.0778 (M + H)^+^ (calculated for C_23_H_22_N_4_O_2_I, 513.0787).

#### (1*S*,4*R*)-4-((1*H*-indol-3-yl)methyl)-8-bromo-1-isopropyl-1,2-dihydro-6*H*-pyrazino[2,1-*b*]quinazoline-3,6(4*H*)-dione (29)

Yield: 10.9 mg, 1.2%; e.r = 50 : 50; mp: 236.5–238.0 °C; [*α*]_D_^30^ = −170 (*c* 0.03; CHCl_3_); *v*_max_ (KBr) 3292, 3193, 2958, 1681, 1666, 1592, 1466, 1237, 832, and 742 cm^−1^; ^1^H NMR (300 MHz, CDCl_3_): *δ* 8. 50 (d, 1H, *J* 2.2 Hz, H-8), 8.05 (br, 1H, H-21), 7.84 (dd, 1H, *J* = 8.7, 2.2 Hz, C-10), 7.41 (d, *J* = 8.7 Hz, H-11), 7.29 (dd, 2H, *J* = 8.0, 2.2 Hz, H-23 & H-26), 7.13 (ddd, 1H, *J* = 8.0, 7.1, 1.0 Hz, H-24), 6.93 (ddd, 1H, *J* = 8.0, 7.1, 1.1 Hz, H-25), 6.62 (d, 1H, *J* = 2.4 Hz, H-20), 5.64 (dd, 1H, *J* = 5.4, 2.8, H-4), 5.63 (s, 1H, H-2), 3.73 (dd, 1H, *J* = 14.9, 2.8 Hz, H-18a), 3.62 (dd, 1H, *J* = 15.0, 5.4 Hz, H-18b), 2.66 (d, *J* = 2.4 Hz, H-1), 2.60 (m, 1H, H-15), 0.65 (d, 3H, *J* = 6.5 Hz, H-16), 0.63 (d, 3H, *J* = 6.4 Hz, H-17); ^13^C NMR (75 MHz, CDCl_3_): *δ* 169.1 (C-3), 159.7 (C-6), 150.9 (C-14), 145.9 (C-12), 138.1 (C-10), 136.2 (C-22), 129.4 (C-8), 129.1 (C-11), 127.2 (C-27), 123.5 (C-20), 122.7 (C-7), 121.5 (C-24), 120.6 (C-25), 120.2 (C-9), 118.7 (C-26), 111.1 (C-23), 109.3 (C-19), 57.0 (C-4), 53.8 (C-1), 29.7 (C-15), 27.3 (C-18), 18.8 (C-16), 14.8 (C-17); (+)-HRMS-ESI *m*/*z*: 465.0987 (M + H)^+^, 487.0726 (M + Na)^+^ (calculated for C_23_H_22_N_4_O_2_Br: 465.0926; C_23_H_21_N_4_O_2_BrNa: 487.0746).

#### (1*S*,4*R*)-4-((1*H*-indol-3-yl)methyl)-8-iodo-1-isobutyl-1,2-dihydro-6*H*-pyrazino[2,1-*b*]quinazoline-3,6(4*H*)-dione (30)

Yield: 62.4 mg, 11.8%; e.r = 51 : 49; mp: 192.1–194.3 °C; [*α*]_D_^30^ = −165 (*c* 0.038; CHCl_3_); *v*_max_ (KBr) 3318, 2956, 1671, 1686, 1593, 1464, 1247, 790, and 740 cm^−1^; ^1^H NMR (300 MHz, CDCl_3_): *δ* 8. 70 (d, 1H, *J* 2.1 Hz, H-8), 8.03 (br, 1H, H-21), 8.03 (dd, 1H, *J* = 8.6, 2.1 Hz, C-10), 7.44 (d, *J* = 7.9 Hz, H-26), 7.33 (d, 1H, *J* = 8.6 Hz, H-11), 7.29 (d, *J* = 7.9 Hz, H-23), 7.13 (t, 1H, *J* = 7.9 Hz, H-24), 6.98 (t, 1H, *J* = 7.9 Hz, H-25), 6.68 (d, 1H, *J* = 2.4 Hz, H-20), 5.96 (s, 1H, H-2), 5.65 (dd, 1H, *J* = 5.2, 2.8, H–), 3.76 (dd, 1H, *J* = 15.0, 2.8 Hz, H-18a), 3.63 (dd, 1H, *J* = 15.0, 5.2 Hz, H-18b), 2.69 (dd, *J* = 9.6, 3.3 Hz, H-1), 2.02–1.92 (m, 1H, H-16), 1.40–1.30 (m, 2H, H-15), 0.79 (d, 3H, *J* = 6.5 Hz, H17a), 0.29 (d, 3H, *J* = 6.4 Hz, H-17b); ^13^C NMR (75 MHz, CDCl_3_): *δ* 169.5 (C-3), 159.4 (C-6), 152.1 (C-14), 146.3 (C-12), 143.4 (C-10), 136.1 (C-22), 135.7 (C-8), 129.2 (C-11), 127.1 (C-27), 123.5 (C-20), 122.9 (C-24), 121.8 (C-7), 120.4 (C-25), 118.7 (C-26), 111.2 (C-23), 109.5 (C-19), 91.5 (C-9), 57.4 (C-4), 51.0 (C-1), 40.1 (C-15), 27.1 (C-18), 24.1 (C-16), 23.3 (C17a), 19.7 (C-17b); (+)-HRMS-ESI *m*/*z*: 527.0936 (M + H)^+^, 549.0748 (M + Na)^+^ (calculated for C_24_H_24_N_4_O_2_I, 527.0944; C_24_H_23_N_4_O_2_INa, 549.0764).

#### (1*S*,4*R*)-4-((1*H*-indol-3-yl)methyl)-8-bromo-1-isobutyl-1,2-dihydro-6*H*-pyrazino[2,1-*b*]quinazoline-3,6(4*H*)-dione (31)

Yield: 64.6 mg, 13.8%; e.r = 51 : 49; mp: 227.0–228.2 °C; [*α*]_D_^30^ = −243 (*c* 0.037; CHCl_3_); *v*_max_ (KBr) 3284, 2959, 1686, 1658, 1599, 1433, 1245, 746, and 684 cm^−1^; ^1^H NMR (300 MHz, CDCl_3_): *δ* 8. 50 (d, 1H, *J* 2.3 Hz, H-8), 8.06 (br, 1H, H-21), 7.84 (dd, 1H, *J* = 8.5, 2.3 Hz, H-10), 7.47 (dd, 2H, *J* = 8.1, 1.9 Hz, H-26), 7.29 (d, *J* = 8.5 Hz, H-23), 7.14 (t, 1H, *J* = 8.0 Hz, H-24), 6.92 (t, 1H, *J* = 7.9 Hz, H-25), 6.65 (d, 1H, *J* = 2.4 Hz, H-20), 5.65 (dd, 1H, *J* = 5.2, 2.9, H-4), 5.71 (s, 1H, H-2), 3.76 (dd, 1H, *J* = 15.0, 2.9 Hz, H18a), 3.63 (dd, 1H, *J* = 15.0, 5.2 Hz, H-18b), 2.70 (dd, *J* = 9.7, 3.3 Hz, H-1), 2.07–1.89 (m, 1H, H-16), 1.38–1.21 (m, 2H, H-15), 0.77 (d, 3H, *J* = 6.3 Hz, H-17a), 0.28 (d, 3H, *J* = 6.5 Hz, H-17b); ^13^C NMR (75 MHz, CDCl_3_): *δ* 169.1 (C-3), 159.7 (C-6), 152.0 (C-14), 145.8 (C-12), 137.8 (C-10), 136.8 (C-22), 129.4 (C-8), 129.1 (C-11). 127.1 (C-27), 123.8 (C-20), 122.9 (C-7), 121.6 (C-24), 120.6 (C-9), 120.4 (C-25), 118.7 (C-26), 111.2 (C-23), 109.6 (C-19), 57.5 (C-4), 50.8 (C-1), 40.1 (C-15), 27.1 (C-18), 24.1 (C-16), 23.3 (C-17a), 19.7 (C-17b); (+)-HRMS-ESI *m*/*z*: 479.1086 (M + H)^+^, 501.0912 (M + Na)^+^ (calculated for C_24_H_24_N_4_O_2_Br, 479.1082; C_24_H_23_N_4_O_2_BrNa, 501.0900).

#### (1*S*,4*R*)-4-((1*H*-indol-3-yl)methyl)-8,10-diiodo-1-isobutyl-1,2-dihydro-6*H*-pyrazino[2,1-*b*]quinazoline-3,6(4*H*)-dione (32)

Yield: 22.5 mg, 3.5%; e.r = 54 : 46; mp: 242.8–243.8 °C; [*α*]_D_^30^ = −229 (*c* 0.032; CHCl_3_); *v*_max_ (KBr) 3313, 2955, 1681, 1599, 1462, 1261, 772, and 669 cm^−1^; ^1^H NMR (300 MHz, DMSO-d_6_): 10.17 (br, 1H, H-21), *δ* 8. 62 (d, 1H, *J* 1.9 Hz, H-8), 8.55 (d, 1H, *J* = 1.9 Hz, H-10), 7.41 (d, 1H, *J* = 8.0 Hz, H-26), 7.33 (d, *J* = 8.0 Hz, H-23), 7.11 (br, H-2), 7.09 (t, 1H, *J* = 7.9 Hz, H-24), 6.91 (t, 1H, *J* = 7.9 Hz, H-25), 6.68 (d, 1H, *J* = 2.3 Hz, H-20), 5.50 (dd, 1H, *J* = 5.2, 2.9, H-4), 3.72 (dd, 1H, *J* = 14.9, 2.9 Hz, H-18a), 3.58 (dd, 1H, *J* = 15.0, 5.2 Hz, H-18b), 2.75 (dd, *J* = 6.6, 5.3 Hz, H-1), 2.11–1.95 (m, 1H, H15a), 1.68–1.53 (m, 1H H-15b), 1.38–1.23 (m, 1H, H-16), 0.62 (t, 3H, *J* = 6.5 Hz, H-17a), 0.47 (d, 3H, *J* = 6.6 Hz, H-17b); ^13^C NMR (75 MHz, DMSO-d_6_): *δ* 168.4 (C-3), 162.0 (C-6), 153.0 (C-12), 151.1 (C-10), 145.2 (C-12), 136.2 (C-22), 135.9 (C-8), 127.1 (C-27), 123.4 (C-20), 121.8 (C-7), 121.5 (C-24), 119.0 (C-25), 117.8 (C-26), 111.0 (C-23), 107.8 (C-19), 91.5 (C-9), 89.2 (C-11), 57.4 (C-4), 50.5 (C-1), 39.4 (C-15), 26.3 (C-18), 23.9 (C-16), 21.8 (C-17a), 20.6 (C-17b); (+)-HRMS-ESI *m*/*z*: 652.9915 (M + H)^+^, 674.9746 (M + Na)^+^ (calculated for C_24_H_23_N_4_O_2_Cl_2_, 652.9910; C_24_H_22_N_4_O_2_Cl_2_Na, 674.9730).

### Microbiology

#### Microbial strains and growth conditions

In the present study, two Gram-positive – *Staphylococcus aureus* ATCC 29213 and *Enterococcus faecalis* ATCC 29212– and two Gram-negative – *Escherichia coli* ATCC 25922 and *Pseudomonas aeruginosa* ATCC 27853 – reference bacterial strains were used. When it was possible to determine a minimal inhibitory concentration (MIC) value for these strains, clinically relevant strains were also used. These included methicillin-resistant *S. aureus* (MRSA) 66/1, isolated from public buses,^64^ and a vancomycin-resistant *Enterococcus* (VRE) strain isolated from river water,^65^*E. faecalis* B3/101. Frozen stocks of all strains were grown on Mueller-Hinton agar (MH – BioKar Diagnostics, Allone, France) at 37 °C for 24 h. All bacterial strains were sub-cultured on MH agar and incubated overnight at 37 °C before each assay, in order to obtain fresh cultures. For the antifungal activity screening, a yeast reference strain *Candida albicans* ATCC 10231, a filamentous fungi reference strain *Aspergillus fumigatus* ATCC 46645, and a dermatophyte clinical strain *Trichophyton rubrum* FF5 were used. For compounds showing some activity in the dermatophyte *T. rubrum*, the activity was enlarged to other species of dermatophytes (*Microsporum canis* FF1 and *Epidermophyton floccosum* FF9). Frozen stocks of all fungal strains were sub-cultured in Sabouraud Dextrose Agar (SDA – BioMèrieux, Marcy L'Etoile, France) before each test, to ensure optimal growth conditions and purity. Stock solutions of each compound (10 mg mL^−1^) were prepared in dimethylsulfoxide (DMSO – Alfa Aesar, Kandel, Germany). In the experiments, the final in-test concentration of DMSO was kept below 1%, as recommended by the CLSI.^66^

### Antimicrobial susceptibility testing

#### Antibacterial activity

An initial screening of the antibacterial activity of the compounds was performed by the Kirby–Bauer disk diffusion method, as recommended by the Clinical and Laboratory Standards Institute (CLSI).^67^ Briefly, sterile 6 mm blank paper disks (Oxoid, Basingstoke, England) impregnated with 15 μg of each compound were placed on inoculated MH agar plates. A blank disk with DMSO was used as a negative control. MH inoculated plates were incubated for 18–20 h at 37 °C. At the end of incubation, the inhibition halos were measured. The minimal inhibitory concentration (MIC) was used to determine the antibacterial activity of each compound, in accordance with the recommendations of the CLSI.^68^ Two-fold serial dilutions of the compounds were prepared in Mueller–Hinton Broth 2 (MHB2 – Sigma-Aldrich, St. Louis, MO, USA) within the concentration range of 0.062–64 μg mL^−1^. Cefotaxime (CTX) ranging between 0.031–16 μg mL^−1^ was used as a control. Sterility and growth controls were included in each assay. Purity check and colony counts of the inoculum suspensions were also performed in order to ensure that the final inoculum density closely approximates the intended number (5 × 10^5^ CFU mL^−1^). The MIC was determined as the lowest concentration at which no visible growth was observed. The minimal bactericidal concentration (MBC) was assessed by spreading 10 μL of culture collected from wells showing no visible growth on MH agar plates. The MBC was determined as the lowest concentration at which no colonies grew after 16–18 h incubation at 37 °C. These assays were performed in duplicate.

#### Antifungal activity

The MIC of each compound was determined by the broth microdilution method, according to CLSI guidelines (reference documents M27-A3 (ref. 45) for yeasts and M38-A2 (ref. 46) for filamentous fungi). Briefly, cell or spore suspensions were prepared in RPMI-1640 broth medium supplemented with MOPS (Sigma-Aldrich, St. Louis, MO, USA) from fresh cultures of the different strains of fungi. In the case of filamentous fungi, the inoculum was adjusted to 0.4–5 × 10^4^ CFU mL^−1^ for *A. fumigatus* ATCC 46645 and to 1–3 × 10^3^ CFU mL^−1^ for the dermatophytes. The inoculum of *C. albicans* was adjusted to 0.5–2.5 × 10^3^ CFU mL^−1^. Two-fold serial dilutions of the compounds were prepared in RPMI-1640 broth medium supplemented with MOPS within the concentration range of 1–128 μg mL^−1^, with maximum DMSO concentration not exceeding 2.5% (v/v). Sterility and growth controls were also included in each assay. The plates were incubated for 48 h at 35 °C (*C. albicans* and *A. fumigatus*) or during 5 days at 25 °C (*T. rubrum*, *M. canis* and *E. floccosum*). MICs were recorded as the lowest concentrations resulting in 100% growth inhibition in comparison to the compound-free controls. Voriconazole MIC for *Candida krusei* ATCC 6258 was used as quality control.^45,46,69,70^ The assay was validated when results obtained were within the recommended limits. The minimal fungicidal concentration (MFC) was determined by spreading 20 μL of culture collected from wells showing no visible growth on SDA plates. The MFC was determined as the lowest concentration showing 100% growth inhibition after 48 h at 35 °C (for *C. albicans* and *A. fumigatus*) or 5 days incubation at 25 °C (*T. rubrum*, *M. canis* and *E. floccosum*). All the experiments were repeated independently at least two times.

### Biofilm formation inhibition assay

For compounds with antibacterial activity, their effect on biofilm formation was evaluated using the crystal violet method. Briefly, bacterial suspensions of 1 × 10^6^ CFU mL^−1^ were prepared in Tryptone Soy broth (TSB-Biokar Diagnostics, Allone, Beauvais, France) supplemented with 1% (p/v) glucose (d(+)-glucose anhydrous for molecular biology, PanReac AppliChem, Barcelona, Spain); and four concentrations of compound were tested: 2 × MIC, MIC, ½ MIC and ¼ MIC, keeping final in-test concentration of DMSO below 1%. A control with inoculum and culture media, a control with appropriate concentration of DMSO, as well as a negative control (TSB alone) were included. Sterile 96-well flat-bottomed untreated polystyrene microtiter plates were used. After a 24 h incubation at 37 °C, the biofilms were heat-fixed for 1 h at 60 °C and stained with 0.5% (v/v) crystal violet (Química Clínica Aplicada, Amposta, Spain) for 5 min. The stain was resolubilized with 33% (v/v) acetic acid (acetic acid 100%, AppliChem, Darmstadt, Germany) and the biofilm biomass was quantified by measuring the absorbance of each sample at 570 nm in a microplate reader (Thermo Scientific Multiskan® EX, Thermo Fisher Scientific, Waltham, MA, USA).^40,43^ Three independent experiments were performed, in triplicate.

### Antimicrobial synergy testing

#### Antibiotic synergy

In order to evaluate the combined effect of the compounds and clinically relevant antimicrobial drugs, a screening was conducted using the disk diffusion method, as previously described.^40^ A set of antibiotic disks (Oxoid, Basingstoke, England) to which the isolates were resistant was selected: cefotaxime (CTX, 30 μg) for extended spectrum beta-lactamase-producer *E. coli* SA/2, oxacillin (OX, 1 μg) for *S. aureus* 66/1, and vancomycin (VA, 30 μg) for *E. faecalis* B3/101. Antibiotic disks alone (controls) and antibiotic disks impregnated with 15 μg of each compound were placed on MH agar plates seeded with the respective bacteria. Sterile 6 mm blank papers impregnated with 15 μg of each compound alone were also tested. A blank disk with DMSO was used as a negative control. MH inoculated plates were incubated for 18–20 h at 37 °C. Potential synergism was recorded when the halo of an antibiotic disk impregnated with a compound was greater than the halo of the antibiotic or compound-impregnated blank disk alone.

#### Antifungal synergy

In order to evaluate the combined effect of the compounds and clinically relevant antifungal drugs, checkerboard assay was conducted, as previously described.^29^ Fluconazole was used in a range between (0.062–4 μg mL^−1^) and compounds were tested in a range between their MIC and progressive two-fold dilutions. Fractional inhibitory concentrations (FIC) were calculated as follows: FIC of compound = MIC of compound in combination with antifungal/MIC of compound alone and FIC of antifungal = MIC of antifungal in combination with compound/MIC of antifungal alone. The FIC index (FICI) was calculated as sum of each FIC and interpreted as follows: FICI ≤ 0.5, synergy; 0.5 < FICI ≤ 4, no interaction; 4 > FICI, antagonism.^71^

### Time–kill kinetics assay

Time–kill kinetics of 26 was performed for *S. aureus* ATCC 29213 and MRSA *S. aureus* 66/1, according to CLSI guidelines.^72^ Briefly, a solution of 64 μg mL^−1^ of 26 was prepared in MHB2, as well as a control with appropriate concentration of DMSO; bacterial suspensions of 5 × 10^5^ CFU mL^−1^ in MHB2 were added and incubated at 37 °C in an orbital shaker. Aliquots of 100 μL were taken at time intervals of 0, 2, 4, 8, 10, 12, and 24 h, and serially diluted (10-fold serial dilutions) in MHB2. Appropriate dilutions were inoculated into MH agar plates and incubated at 37 °C for 24 h. After incubation, colony counts were performed, and log_10_ CFU mL^−1^ was plotted against time.

### Scanning electron microscopy (SEM)

The effect of 26 on *S. aureus* ATCC 29213 and MRSA *S. aureus* 66/1 cellular surface was observed using scanning electron microscope.^73^ Briefly, solutions of 26 at MIC and ½ MIC were prepared in MHB2, as well as controls with appropriate concentration of DMSO; bacterial suspensions of 5 × 10^5^ CFU mL^−1^ in MHB2 were added and incubated at 37 °C for 20 h. 500 μL of each sample was centrifuged at 4 °C for 6 min at 10 000 rpm and washed 3 times with phosphate-buffered saline 0.01 M, pH 7.4 (Sigma, St. Louis, MO, USA), fixed in 2.5% (v/v) glutaraldehyde (glutaraldehyde solution 25%; Merck, Darmstadt, Germany) in 0.1 M sodium cacodylate buffer, pH 7.2 (Sodium cacodylate trihydrate ≥ 98%; Sigma, St. Louis, MO, USA) for 2h30 min at 4 °C, washed three times with sodium cacodylate buffer and post-fixed overnight in 1% (v/v) osmium tetroxide (OsO_4_ 4% solution; Agar Scientific, Stansted, UK) in the same buffer. Subsequently, samples were washed two times with sodium cacodylate buffer and one time with ultrapure sterile water; following that, samples were resuspended in ultrapure sterile water, and transferred to 12 mm cover slips previously coated with 5% (v/v) APES solution [3-(triethoxysilyl)propylamine; Merck, Darmstadt, Germany] in acetone (Benzina Internacional, Maia, Portugal). The samples were then dehydrated in 30, 50, 70, 90, 95, 100% ethanol (Merck, Darmstadt, Germany) (3 times for 5 min for each concentration), and dried with a critical point dryer. Samples were coated with an Au/Pd thin film, by sputtering, using the SPI Module Sputter Coater equipment. The SEM/EDS examination was performed using a High resolution Scanning Electron Microscope with X-ray Microanalysis: JEOL JSM 6301F/Oxford INCA Energy 350.

### Enantioselective liquid chromatography

#### Quantitative analysis of enantioselective liquid chromatography

Compounds 22–32 were prepared using HPLC grades *n*-hexane : EtOH (50 : 50) at a final concentration 50 μg mL^−1^, and the injection volume was 10 μL. The HPLC system comprised a JASCO model 880-PU intelligent HPLC pump (JASCO corporation, Tokyo, Japan), equipped with a 7125 injector (Rheodyne LCC, Rohnert Park, CA, USA) fitted with a 20 μL LC loop, a JASCO model 880–30 solvent mixer involving a 875-UV intelligent UV/VIS detector, a system equipped with a chiral column (Lux® 5 μm amylose-1, 250 × 4.6 mm). The data acquisitions were performed using ChromNAC chromatography Data system (version 1.19.1) from JASCO Corporation (Tokyo, Japan). The mobile phase consisted of the mixture of *n*-hexane : EtOH (90 : 10, v/v), at a flow rate of 0.5 mL min^−1^. The mobile phase was prepared in a volume/volume ratio and degassed in an ultrasonic bath for at least 15 min before use. Chromatographic analyses were carried out in isocratic mode at 22 ± 2 °C, in duplicate. The UV detection was performed at a wavelength of 254 nm. The volume void time was considered to be equal to the peak of solvent front and was taken from each particular run. The enantiomeric ratios (e.r) were determined by the mean percentage of peak area of eluted peaks.

#### Semi-preparative enantioselective resolution

Compounds 22, 23, and 26 were prepared in the mixture of HPLC grade solvent *n*-hexane : EtOH (50 : 50) at a concentration of 10 mg mL^−1^, and the injection volume was 100–200 μL. The HPLC system is similar to that described in quantitative analysis, equipped with an in-house column amylose tris-3,5-dimethylphenylcarbamate coated with Nucleosil (500 A, 7 mm, 20%, w/w) packed into a stainless steel (200 mm × 7 mm I.D. size) column, prepared in the UFSCar laboratory.^63^ Semi-preparative chromatographic separations were first achieved through multiple injection with 200 μL at a flow rate of 2 mL min^−1^. Chromatographic analyses were carried out in isocratic mode at 22 ± 2 °C. The UV detection was performed at a wavelength of 254 nm. The collected fractions were analyzed using the analytical column to determine their enantiomeric ratio/excess with the conditions described above.

### Cell-based studies

#### SH-SY5Y cell culture and differentiation

SH-SY5Y cells (ATCC, United States of America) were routinely cultured in 25 cm^2^ flasks using DMEM with 4.5 g L^−1^ glucose supplemented with 10% heat-inactivated FBS, 100 U mL^−1^ of penicillin and 100 μg mL^−1^ of streptomycin. Cells were maintained in a 5% CO_2_ to 95% air atmosphere, at 37 °C, and the medium was changed every 2–3 days. When 80–90% confluence was reached, the cultures were passaged by trypsinization (0.25% trypsin/1 mM EDTA). In all experiments, the cells were seeded in 96 well plates at a density of 25 000 cells per cm^2^ and used 6 days after seeding. To obtain cells with a dopaminergic neuronal phenotype, SH-SY5Y cells were differentiated as previously described.^74^ Briefly, SH-SY5Y cells were seeded in complete DMEM medium containing 10 μM RA, and cultured for 3 days at 37 °C. After 3 days, 80 nM of TPA was added to the cultures, and cells were cultured for another 3 days, at 37 °C. The non-differentiated SH-SY5Y cells were maintained in complete DMEM medium for 6 days, mimicking the differentiation protocol but in the absence of both RA and TPA. The cells used in all experiments were taken between the 21^st^ and 25^th^ passages.

#### Evaluation of cytotoxicity of 26

Cytotoxity of 26 (0–25 μM) was evaluated in both differentiated and non-differentiated SH-SY5Y cells, by the neutral red (NR) uptake, resazurin (REZ) reduction and sulforhodamine B (SRB) assays, 24 h after exposure. For that purpose, SH-SY5Y cells were seeded in 96-well plates at a density of 25 000 cells per cm^2^, submitted or not to a RA and TPA differentiation protocol, and exposed, 6 days after seeding to 26 (0–25 μM), in fresh cell culture medium. Triton™ X-100 (0.1%) was used as positive control.

#### Neutral red uptake assay

The neutral read (NR) uptake assay, which is based on the capacity of viable cells to incorporate and bind the weak cationic dye NR into the lysosomes, provides a quantitative estimation of the number of viable cells in a culture.^75,76^ The NR dye is first incorporated into the cells according to their lysosomal functionality, and then extracted from the viable cells using an acidified ethanol solution, being the absorbance of the solubilized dye quantified using a spectrophotometer.^77^ Twenty-four hours after exposure to 26, the cell culture medium was removed, followed by the addition of fresh cell culture medium containing 50 μg mL^−1^ NR, and incubation at 37 °C, in a humidified 5% CO_2_ to 95% air atmosphere, for 60 minutes. After the 60 min incubation with NR, the cell culture medium was removed, the dye absorbed only by viable cells extracted with lysis buffer [absolute ethyl alcohol/distilled water (1 : 1) with 5% acetic acid], and the absorbance measured at 540 nm in a multiwell plate reader (PowerWaveX BioTek Instruments, Vermont, USA). The percentage of NR uptake relatively to that of the control cells (0 μM) was used as the cytotoxicity measure. Four independent experiments were performed, in triplicate.

#### Resazurin reduction assay

The resazurin (REZ) reduction assay represents a simple, rapid, and sensitive method for the evaluation of cell's viability. It is based on the ability of metabolically active cells to reduce the nonfluorescent dye REZ to the strongly-fluorescent dye resorufin, being the fluorescence proportional to the number of viable cells in culture.^78^ After incubation with compound 26 for 24 h, the cell culture medium was removed, followed by the addition of fresh cell culture medium containing 10 μg mL^−1^ REZ, and incubation at 37 °C, in a humidified 5% CO_2_ to 95% air atmosphere, for 60 minutes. The fluorescence was then read in a multiwell plate reader (PowerWaveX BioTek Instruments, Vermont, USA), using an excitation and emission wavelengths of 560 nm and 590 nm, respectively. The percentage of REZ reduction relatively to that of the control cells (0 μM) was used as the cytotoxicity measure. Four independent experiments were performed, in triplicate.

#### Sulforhodamine B assay

The sulforhodamine B (SRB) assay has been widely used to measure drug-induced cytotoxicity and cell proliferation, and it is based on the ability of the protein dye SRB to bind stoichiometrically to protein basic amino acid residues under mild acidic conditions. The bound SRB can then be extracted using basic conditions, and the amount of bound SRB can be used as a proxy for cell mass.^79^ After incubation with 26 for 24 h, the cell culture medium was removed, the cells washed with HBSS (+/+) and fixed overnight at −20 °C with a methanolic solution of 1% acetic acid (v/v). The fixing medium was then removed, replaced by 0.05% SRB solution (prepared in 1% acetic acid), followed by incubation at 37 °C for 60 minutes. After incubation, the SRB solution was removed and the cells washed with 1% acetic acid (v/v) to remove unbound dye. The plates were let to air-dry at room temperature and the bound SRB extracted with a Tris base solution (10 mM, pH 10.5). The absorbance was measured at 540 nm in a multiwell plate reader (PowerWaveX BioTek Instruments, Vermont, USA). The percentage of SRB binding relatively to that of the control cells (0 μM) was used as the cytotoxicity measure. Three independent experiments were performed, in triplicate.

#### Statistical analysis

In the cytotoxicity assays, GraphPad Prism 6 for Windows (GraphPad Software, San Diego, CA, USA) was used to perform all statistical calculations. Three tests were performed to evaluate the normality of the data distribution: KS, D'Agostino & Pearson omnibus and Shapiro–Wilk normality tests. For the cytotoxicity results, one-way ANOVA was used to perform the statistical comparisons, followed by the Dunnett's multiple comparisons test. Details of the performed statistical analysis are described in the figure legend. Differences were considered to be significant for *p* values lower than 0.05.

#### Docking

The 3D structures of the 22 test molecules were drawn using HyperChem 7.5, being minimized by the semi-empirical Polak–Ribiere conjugate gradient method (RMS < 0.1 kcal Å^−1^ mol^−1^). Docking simulations between GyrB (pdb code 3u2d), and FtsZ (pdb code 4dxd), and the small molecules and test compounds 08B (3u2d co-crystallized 4-bromo-5-methyl-*N*-[1-(3-nitropyridin-2-yl)piperidin-4-yl]-1*H*-pyrrole-2-carboxamide^53^), and 9PC (4dxd co-crystallized 3-[(6-chloro[1,3]thiazolo[5,4-*b*]pyridin-2-yl)methoxy]-2,6-difluorobenzamide^80^) were undertaken in AutoDock Vina (Scripps Research Institute, USA).^81^ The ligands and proteins were prepared using AutoDock Tools 1.5.6.^82^ The proteins were added polar hydrogen and given Kollman charge, whereas the ligands were given Gasteiger charges. AutoDock Vina considered the target conformation as a rigid unit while the ligands were allowed to be flexible and adaptable to the target. Vina searched for the lowest binding affinity conformations and returned 9 different conformations for each ligand. AutoDock Vina was run using an exhaustiveness of 8 and a grid box with the dimensions of 15.0 × 15.0 × 15.0 Å, engulfing the crystallographic ligand were used. Conformations and interactions were visualized using PyMOL version 1.3.

#### Molecular dynamics simulation (MD)

A 5Å spherical droplet containing 100 water molecules was placed surrounding two conformations of 26 obtained from the docking study on GyrB; the complexes were energy minimized using the MMFF94x force field^83^ until RMS gradient < 0.1 kcal Å^−1^. mol^−1^. The 26:GyrB complexes were then subjected to MD simulation using MOE-dynamic implemented in MOE 2014.09 (Chemical Computing Groups, Montreal, Canada).^84^ MD simulation^85,86^ was done by choosing MMFF94x force field and NVT (*N*, total atom; *V*, volume; *T*, temperature) ensemble and Nosé–Poincaré–Andersen (NPA) algorithm,^87^ with 0.002 ps time step and sampling every 0.5 ps. The system was heated from 0 K to 300 K in 100 ps (heat stage), followed by a 2000 ps of a production stage at 300 K; the system was then cooled back to 0 K in 100 ps (cooling stage).^88^ Simulation observation was done by examining the 26:GyrB complex interaction between ligand atoms and target atoms at the end of the simulation. The system behaviour was monitored by the analysis of the potential energy over the course of the simulation.

## Conflicts of interest

There are no conflicts to declare.

## Supplementary Material

RA-010-D0RA05319H-s001
