# Supplementary material for: New marine-derived indolymethyl pyrazinoquinazoline alkaloids with promising antimicrobial profiles
Source: RSC Adv. 2020 Aug 21;10(52):31187–204. doi: 10.1039/d0ra05319h (PMC9056383; doi:10.1039/d0ra05319h)
Supplement: RA-010-D0RA05319H-s001 [file RA-010-D0RA05319H-s001.pdf]

Electronic Supplementary Material (ESI) for RSC Advances.
This journal is © The Royal Society of Chemistry 2020

Supplementary information

New Marine-Derived Indolymethyl Pyrazinoquinazoline Alkaloids with Promising Antimicrobial Profiles

Solida Long[a], Diana I. S. P. Resende[a,b], Andreia Palmeira[a,b], Anake Kijjoa[b,c], Artur M. S. Silva[d], Maria Elizabeth Tiritan[a,b,e], Patrícia Pereira-Terra[b,c], Joana Freitas-Silva[b,c], Sandra Barreiro[f], Renata Silva[f], Fernando Remião[f], Eugénia Pinto[b,g], Paulo Martins da Costa[b,c], Emília Sousa[a,b*], and Madalena M. M. Pinto[a,b]*

[a]LQOF - Laboratório de Química Orgânica e Farmacêutica, Departamento de Ciências Químicas, Faculdade de Farmácia, Universidade do Porto, Rua de Jorge Viterbo Ferreira, 228, 4050-313 Porto, Portugal;
[b]CIIMAR - Centro Interdisciplinar de Investigação Marinha e Ambiental, Terminal de Cruzeiros do Porto de Leixões, Av. General Norton de Matos S/N, 4450-208 Matosinhos, Portugal;
[c]ICBAS - Instituto de Ciências Biomédicas Abel Salazar, Universidade do Porto, Rua de Jorge Viterbo Ferreira, 228, 4050-313 Porto, Portugal;
[d]QOPNA - Química Orgânica, Produtos Naturais e Agroalimentares, Departamento de Química, Universidade de Aveiro, 3810-193 Aveiro, Portugal;
[e]CESPU, Instituto de Investigação e Formação Avançada em Ciências e Tecnologias da Saúde (IINFACTS), Rua Central de Gandra, 1317, 4585-116 Gandra PRD, Portugal;
[f]UCIBIO-REQUIMTE, Laboratório de Toxicologia, Departamento de Ciências Biológicas, Faculdade de Farmácia, Universidade do Porto, Rua de Jorge Viterbo Ferreira, 228, 4050-313 Porto, Portugal;
[g]Laboratório de Microbiologia, Departamento de Ciências Biológicas, Faculdade de Farmácia, Universidade do Porto, Rua de Jorge Viterbo Ferreira, 228, 4050-313 Porto, Portugal
*Corresponding authors: esousa@ff.up.pt, pmcosta@icbas.up.pt

Contents

1. NMR Spectra 2
2. Enantioselective liquid chromatography 19
   2.1 Chiral analysis of compounds 22-32 19
   2.2 Enantioselective liquid chromatography separation of compounds 22, 23, and 26 24
3. High resolution mass spectra 26
4. Antimicrobial activity 32

1. NMR Spectra

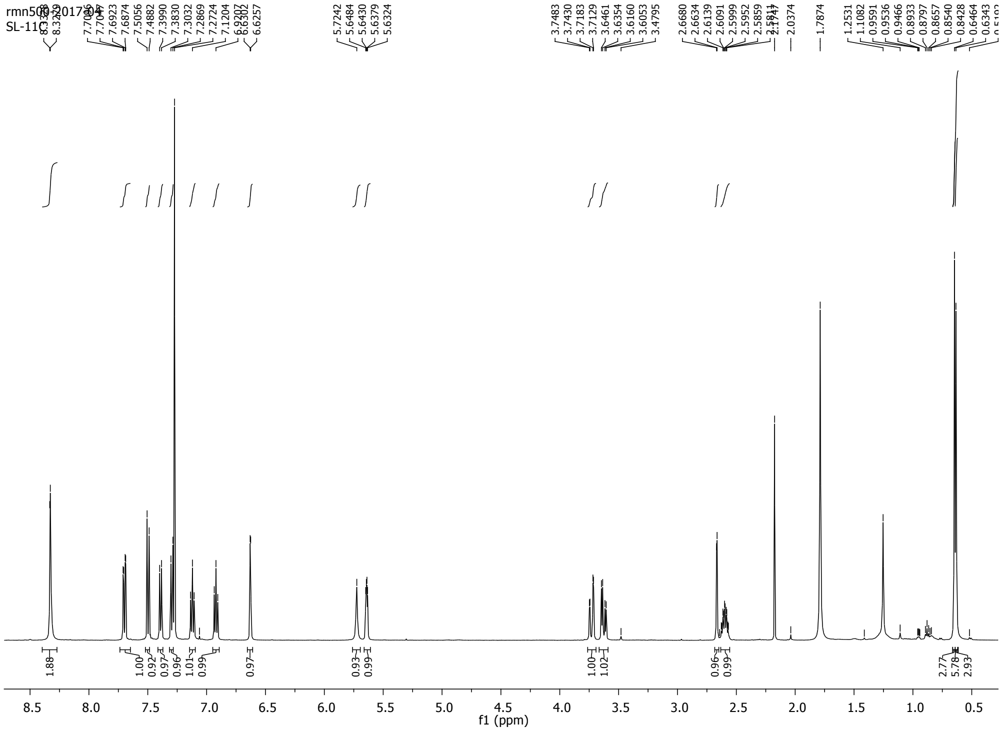

Fig. S1. ^{1}H NMR spectrum of (1*S*,4*R*)-4-((1*H*-indol-3-yl)methyl)-8-chloro-1-isopropyl-1,2-dihydro-6*H*-pyrazino[2,1-b]quinazoline-3,6(4*H*)-dione (**22**) ($CDCl_3$, 300, MHz).

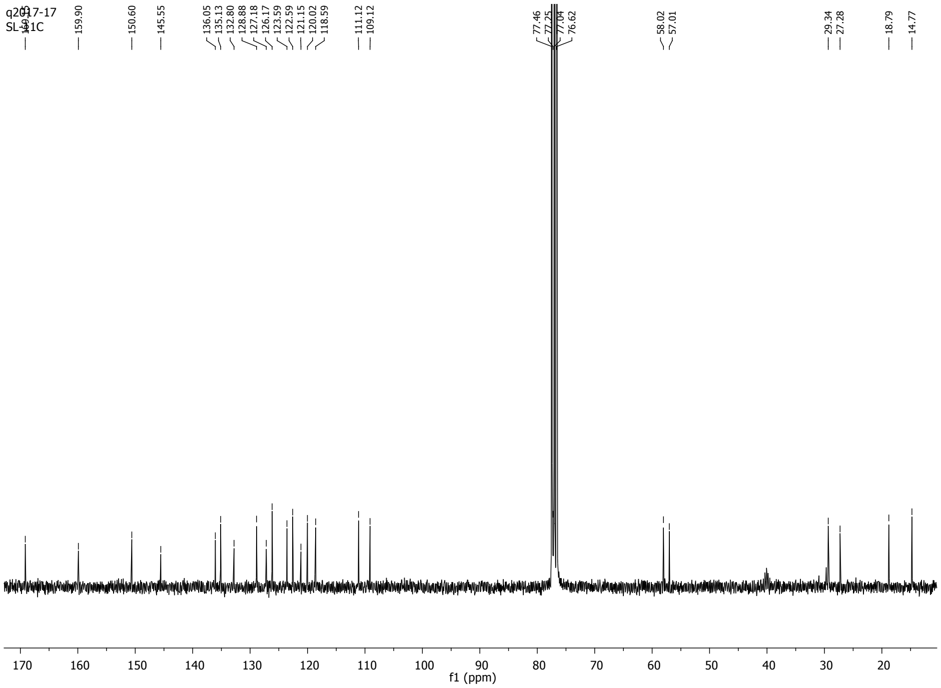

Fig. S2. ^{13}C NMR spectrum of (1*S*,4*R*)-4-((1*H*-indol-3-yl)methyl)-8-chloro-1-isopropyl-1,2-dihydro-6*H*-pyrazino[2,1-b]quinazoline-3,6(4*H*)-dione (**22**) ($CDCl_3$, 75, MHz).

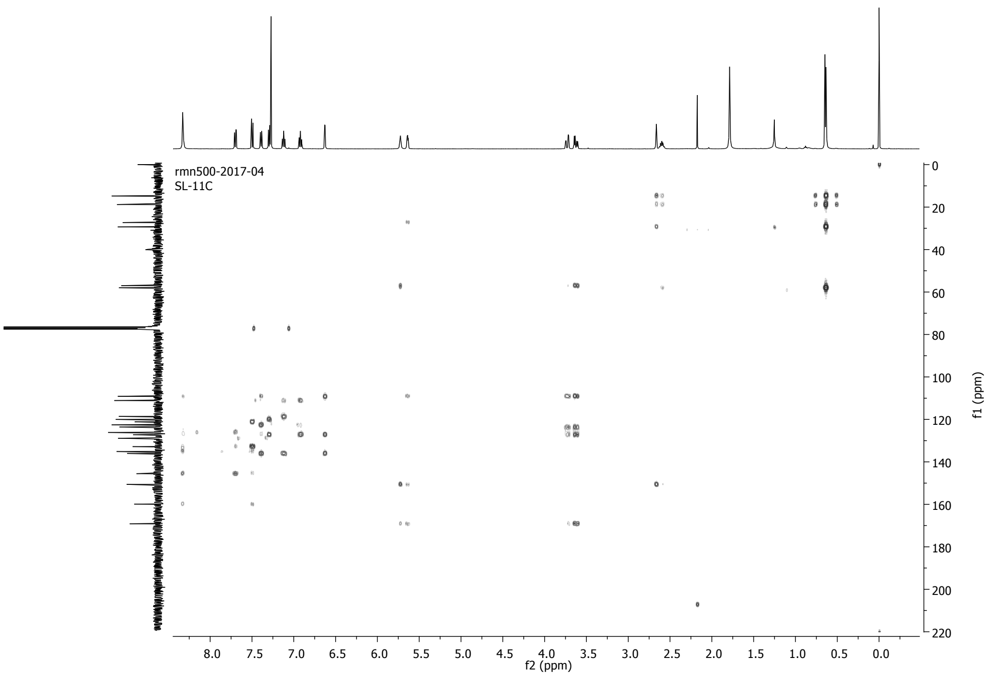

Fig. S3. ^{1}H NMR spectrum of (1*S*,4*R*)-4-((1*H*-indol-3-yl)methyl)-8-chloro-1-isopropyl-1,2-dihydro-6*H*-pyrazino[2,1-b]quinazoline-3,6(4*H*)-dione (**22**) ($CDCl_3$, 300, MHz).

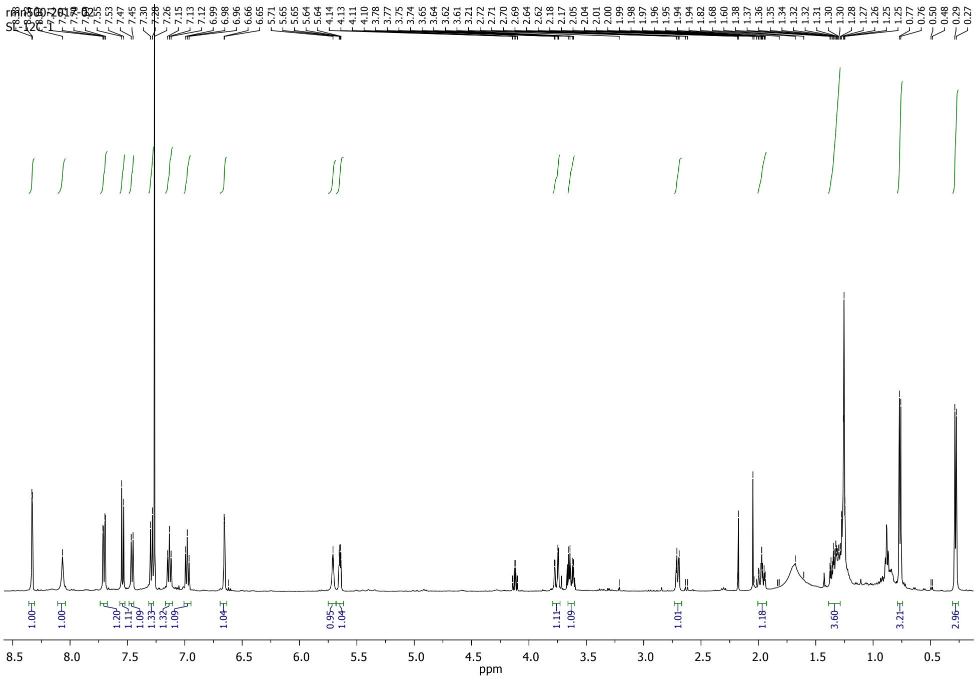

Fig. S4. ^{1}H NMR spectrum of (1*S*,4*R*)-4-((1*H*-indol-3-yl)methyl)-8-chloro-1-isobutyl-1,2-dihydro-6*H*-pyrazino[2,1-b]quinazoline-3,6(4*H*)-dione (**23**) ($CDCl_3$, 300, MHz).

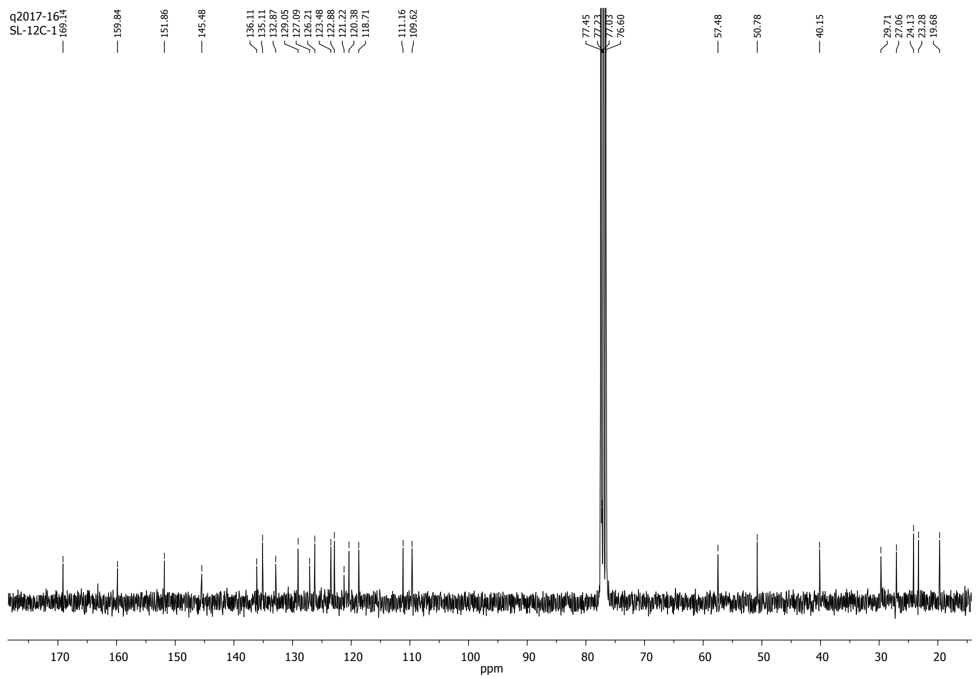

Fig. S5. ^{13}C NMR spectrum of (1*S*,4*R*)-4-((1*H*-indol-3-yl)methyl)-8-chloro-1-isobutyl-1,2-dihydro-6*H*-pyrazino[2,1-b]quinazoline-3,6(4*H*)-dione (**23**) ($CDCl_3$, 75, MHz).

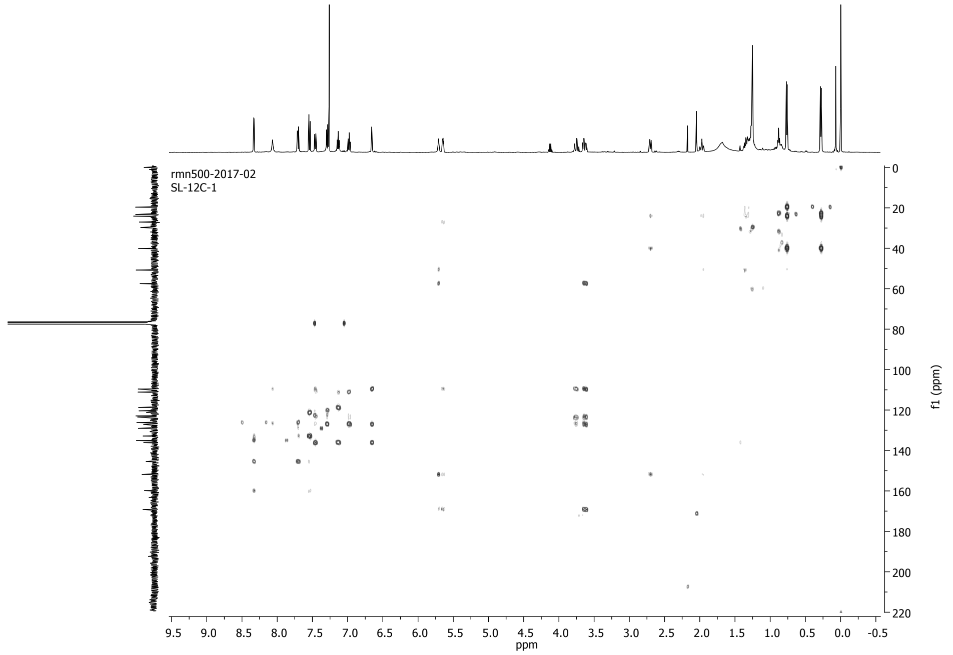

Fig. S6: HMBC NMR spectrum of (1*S*,4*R*)-4-((1*H*-indol-3-yl)methyl)-8-chloro-1-isobutyl-1,2-dihydro-6*H*-pyrazino[2,1-b]quinazoline-3,6(4*H*)-dione (**23**) ($CDCl_3$, 300, MHz).

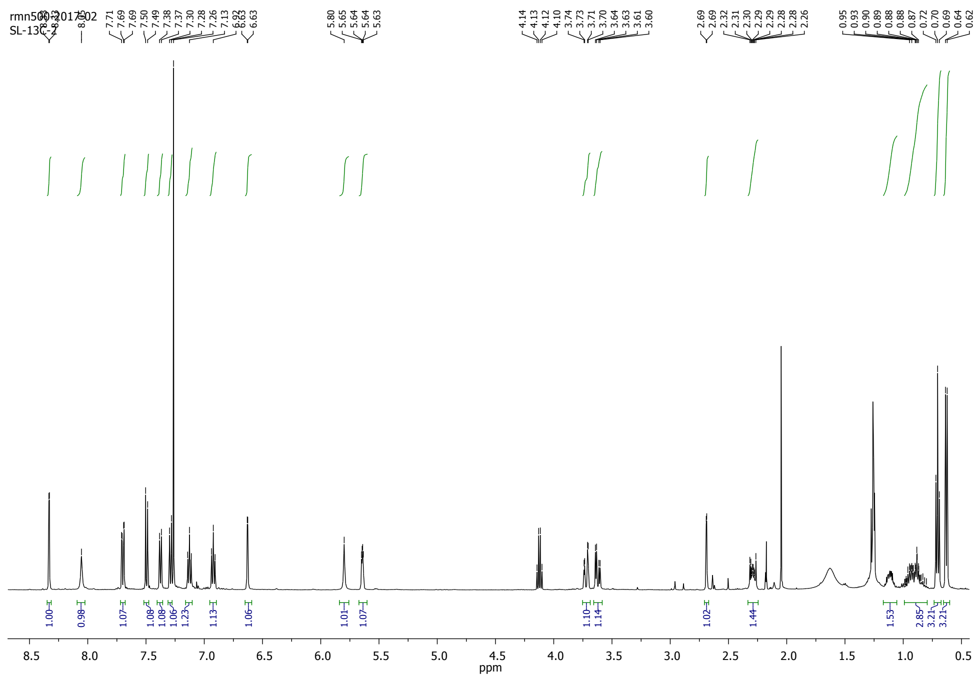

Fig. S7. ^{1}H NMR spectrum of (1*S*,4*R*)-4-((1*H*-indol-3-yl)methyl)-1-((*S*)-sec-butyl)-8-chloro-1,2-dihydro-6*H*-pyrazino[2,1-*b*]quinazoline-3,6(4*H*)-dione (**24**) ($CDCl_3$, 300, MHz).

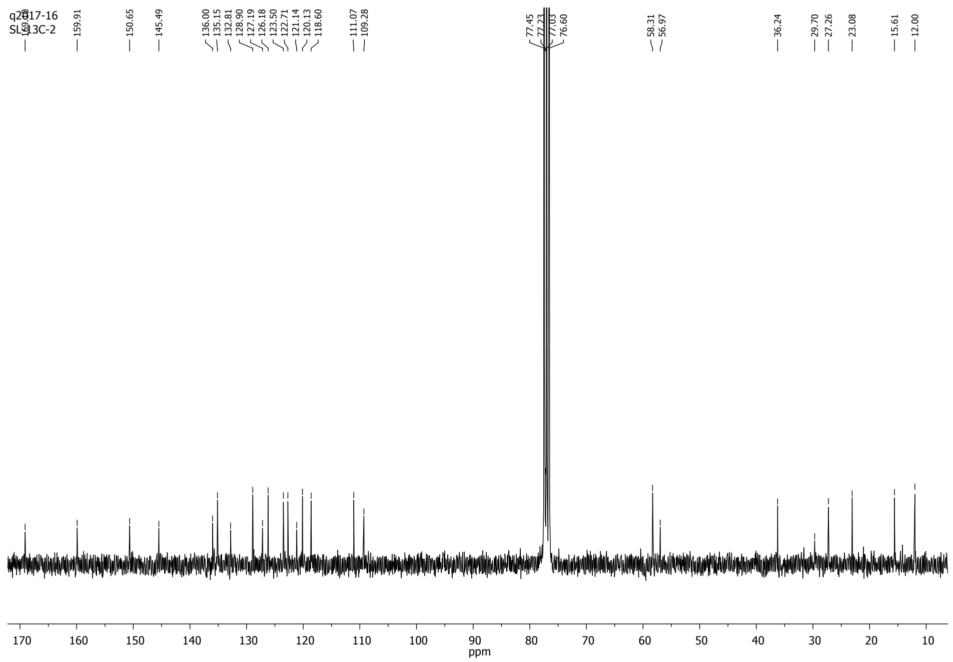

Fig. S8. ^{13}C NMR spectrum of (1*S*,4*R*)-4-((1*H*-indol-3-yl)methyl)-1-((*S*)-sec-butyl)-8-chloro-1,2-dihydro-6*H*-pyrazino[2,1-*b*]quinazoline-3,6(4*H*)-dione (**24**) ($CDCl_3$, 75, MHz).

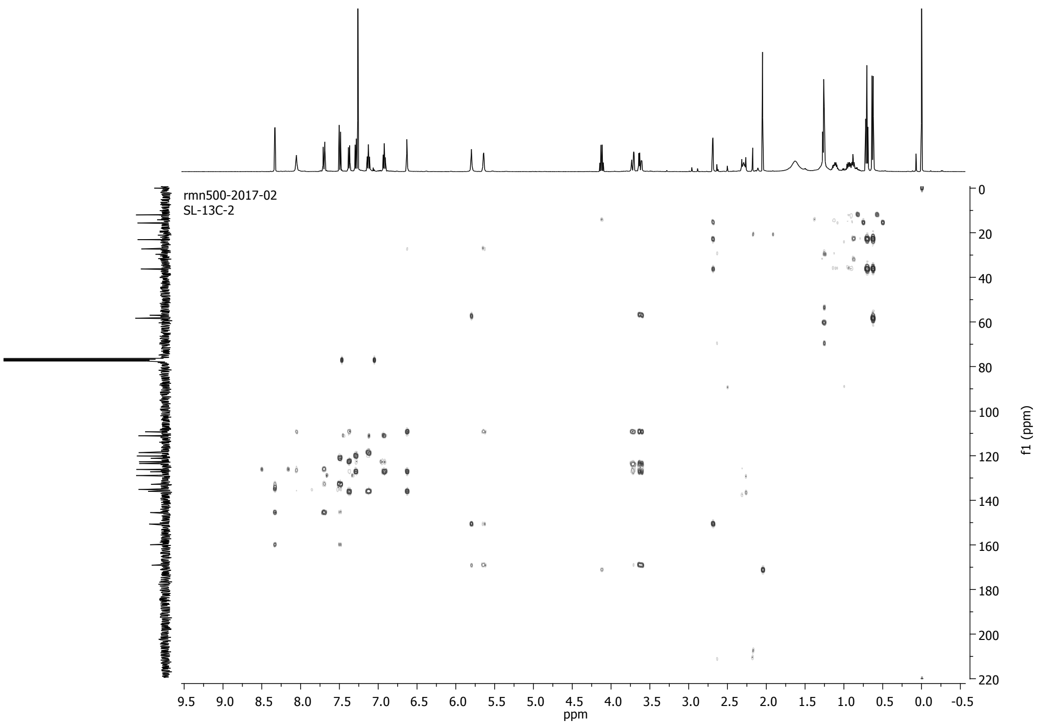

Fig. S9. HMBC NMR spectrum of (1*S*,4*R*)-4-((1*H*-indol-3-yl)methyl)-1-((*S*)-sec-butyl)-8-chloro-1,2-dihydro-6*H*-pyrazino[2,1-*b*]quinazoline-3,6(4*H*)-dione (**24**) ($CDCl_3$, 300, MHz).

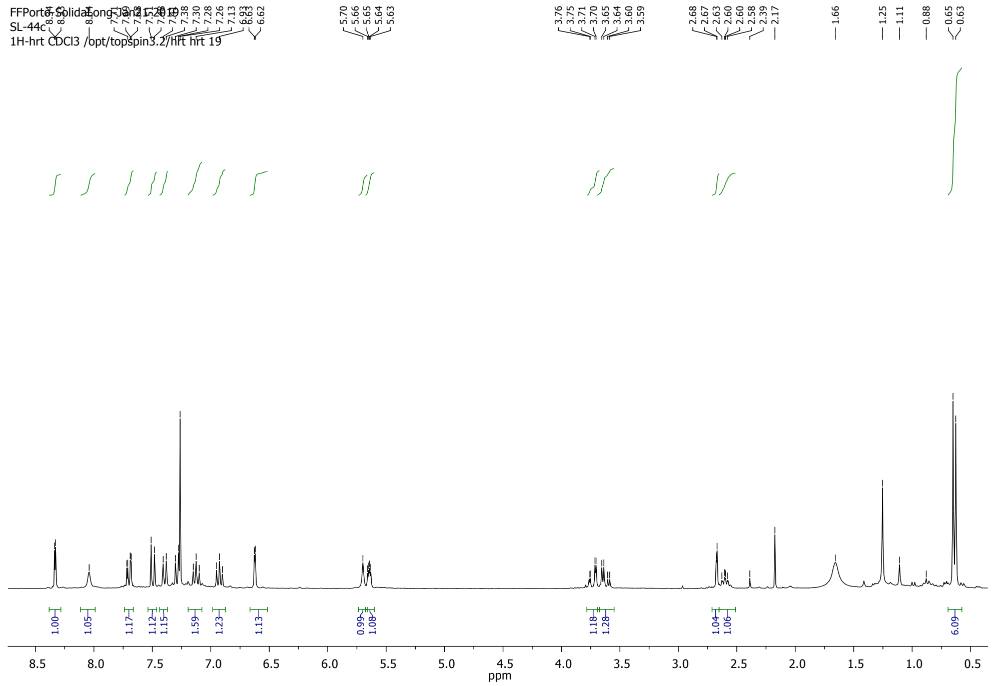

Fig. S10. ^{1}H NMR NMR spectrum of (1*S*,4*R*)-4-((1*H*-indol-3-yl)methyl)-8,10-dichloro-1-isopropyl-1,2-dihydro-6*H*-pyrazino[2,1-*b*]quinazoline-3,6(4*H*)-dione (**25**) ($CDCl_3$, 300, MHz).

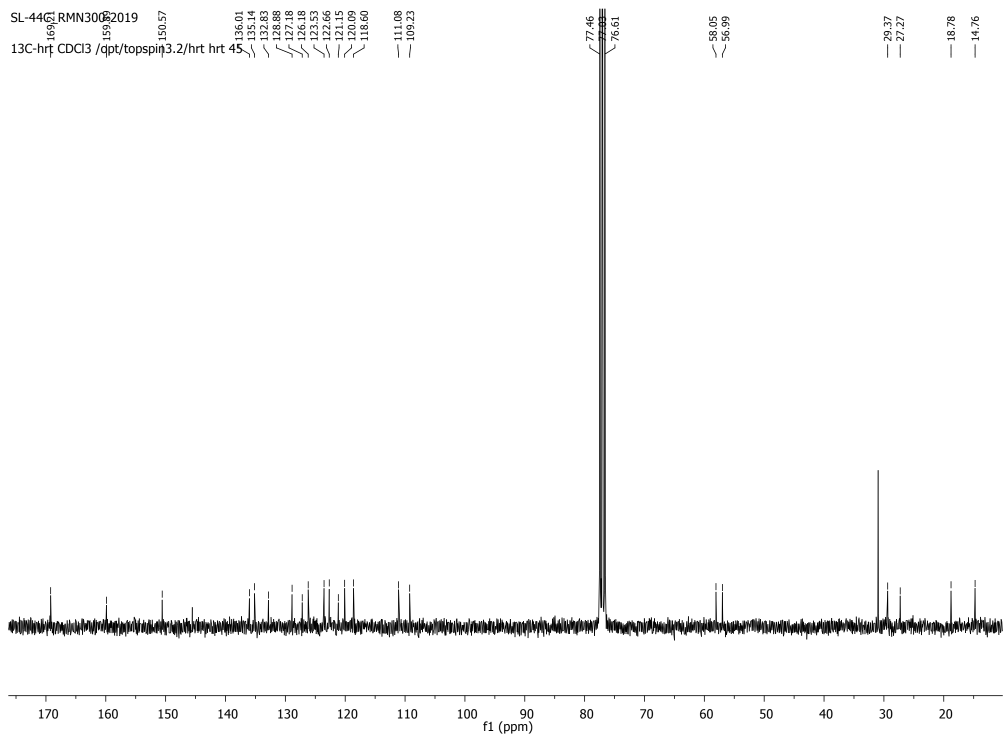

Fig. S11. ^{13}C NMR NMR spectrum of (1*S*,4*R*)-4-((1*H*-indol-3-yl)methyl)-8,10-dichloro-1-isopropyl-1,2-dihydro-6*H*-pyrazino[2,1-*b*]quinazoline-3,6(4*H*)-dione (**25**) ($CDCl_3$, 75, MHz).

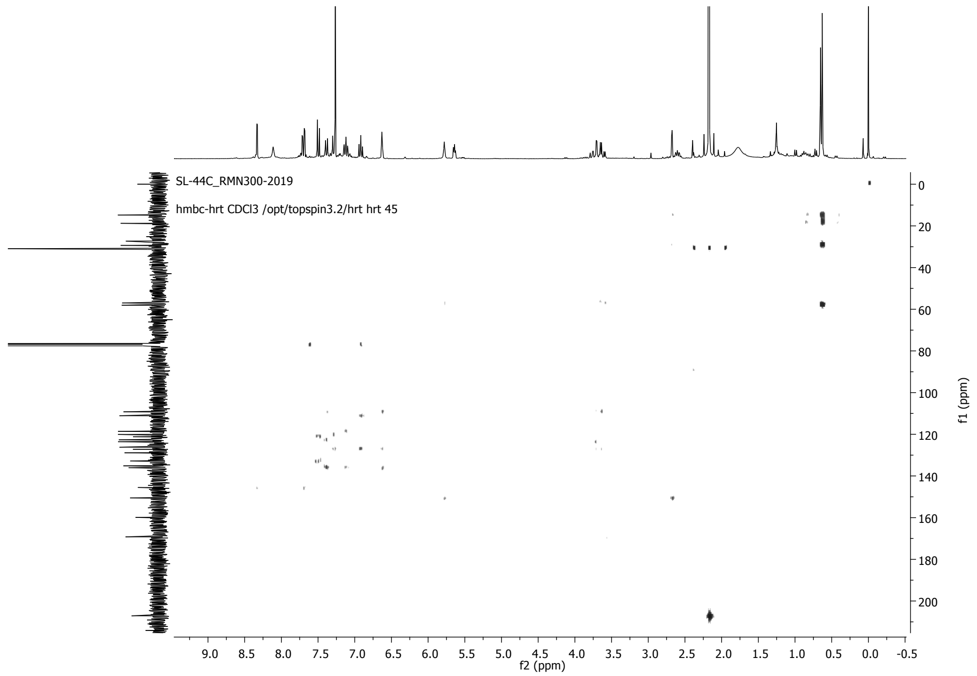

Fig. S12. HRMS NMR spectrum of (1*S*,4*R*)-4-((1*H*-indol-3-yl)methyl)-8,10-dichloro-1-isopropyl-1,2-dihydro-6*H*-pyrazino[2,1-*b*]quinazoline-3,6(4*H*)-dione (**25**) ($CDCl_3$, 300, MHz).

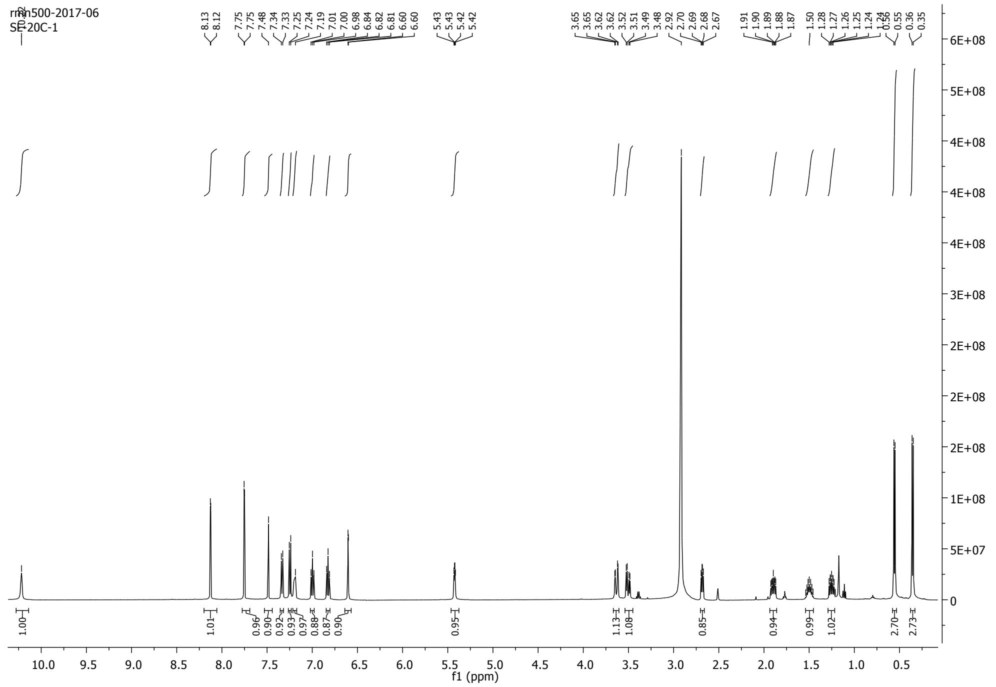

Fig. S13. ^{1}H NMR NMR spectrum of (1*S*,4*R*)-4-((1*H*-indol-3-yl)methyl)-8,10-dichloro-1-isobutyl-1,2-dihydro-6*H*-pyrazino[2,1-*b*]quinazoline-3,6(4*H*)-dione (**26**) (DMSO-d_6, 300, MHz).

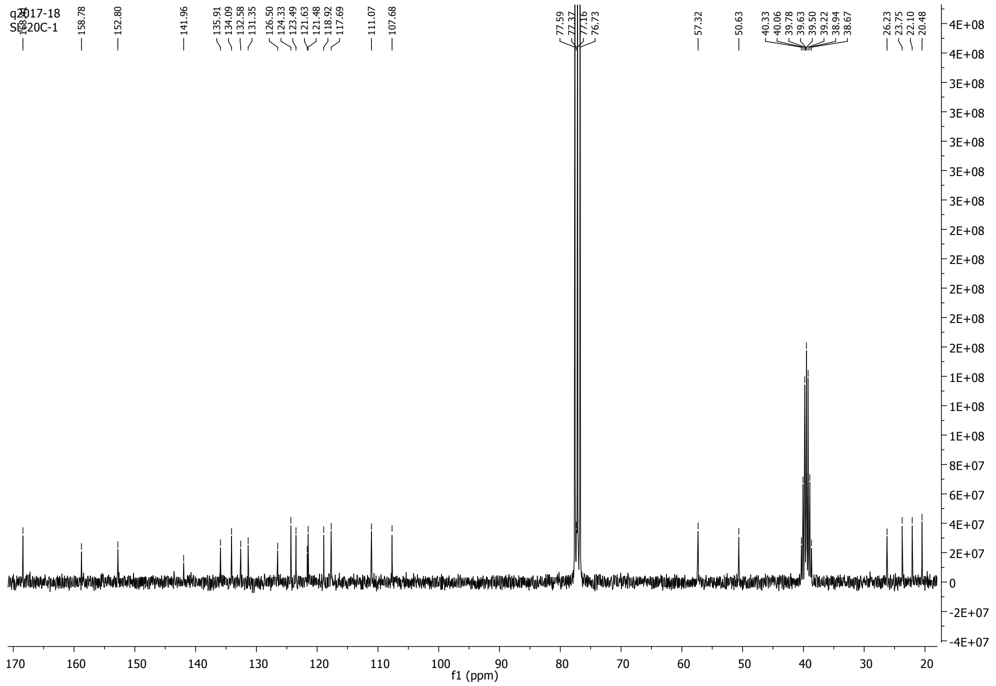

Fig. S14. ^{13}C NMR NMR spectrum of (1*S*,4*R*)-4-((1*H*-indol-3-yl)methyl)-8,10-dichloro-1-isobutyl-1,2-dihydro-6*H*-pyrazino[2,1-*b*]quinazoline-3,6(4*H*)-dione (**26**) (DMSO-d_6, 75, MHz).

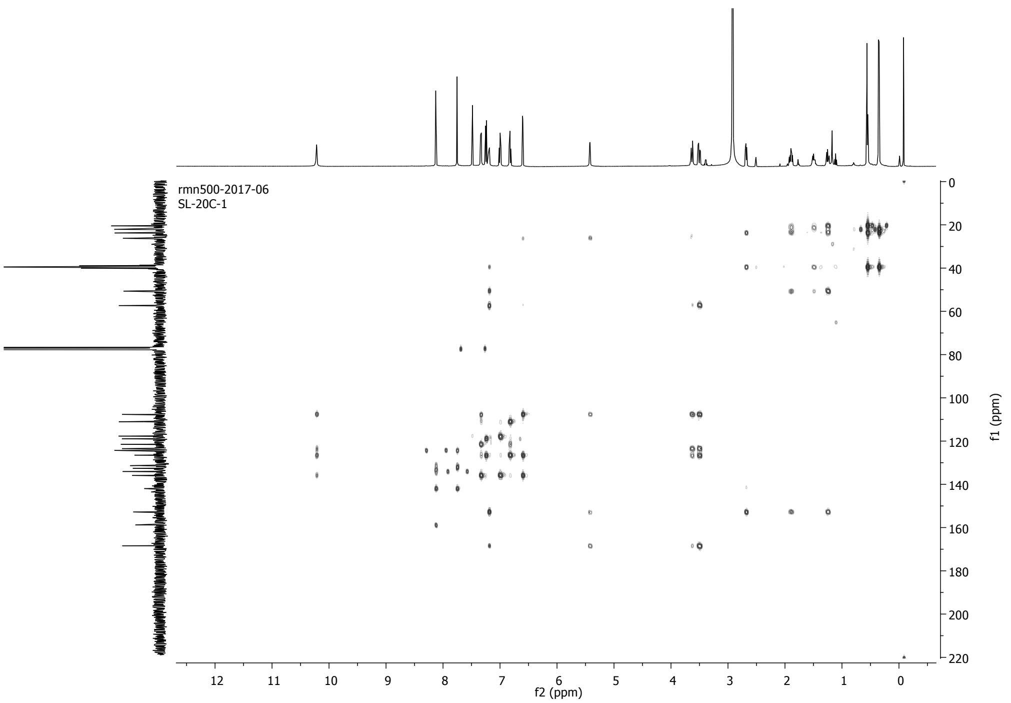

Fig. S15. HMBC NMR spectrum of (1*S*,4*R*)-4-((1*H*-indol-3-yl)methyl)-8,10-dichloro-1-isobutyl-1,2-dihydro-6*H*-pyrazino[2,1-*b*]quinazoline-3,6(4*H*)-dione (**26**) (DMSO-d_6, 300, MHz)

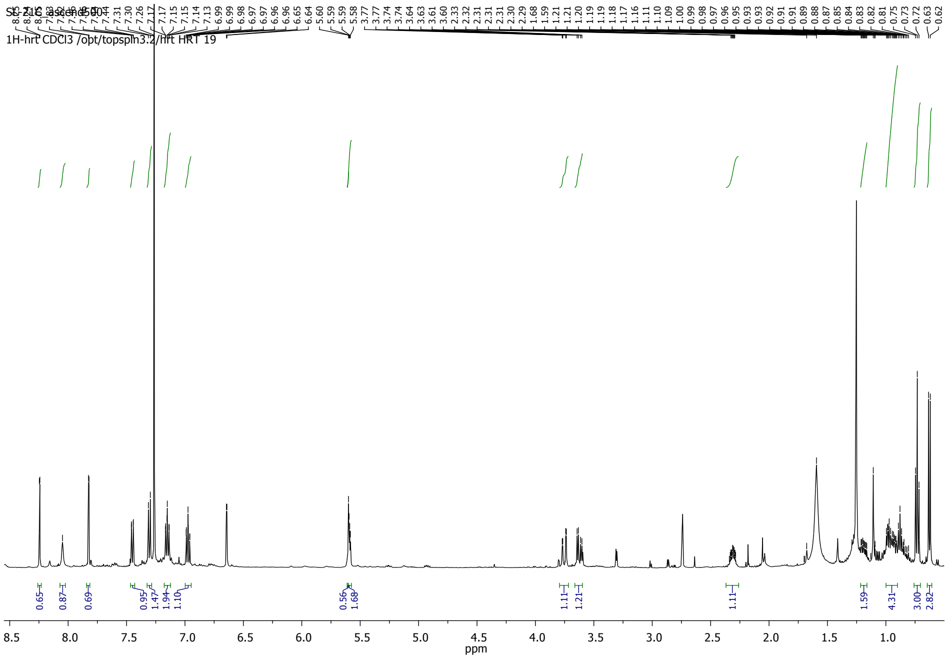

Fig. S16. ^{1}H NMR NMR spectrum of (1*S*,4*R*)-4-((1*H*-indol-3-yl)methyl)-1-((*S*)-sec-butyl)-8,10-dichloro-1,2-dihydro-6*H*-pyrazino[2,1-*b*]quinazoline-3,6(4*H*)-dione (**27**) ($CDCl_3$, 300, MHz).

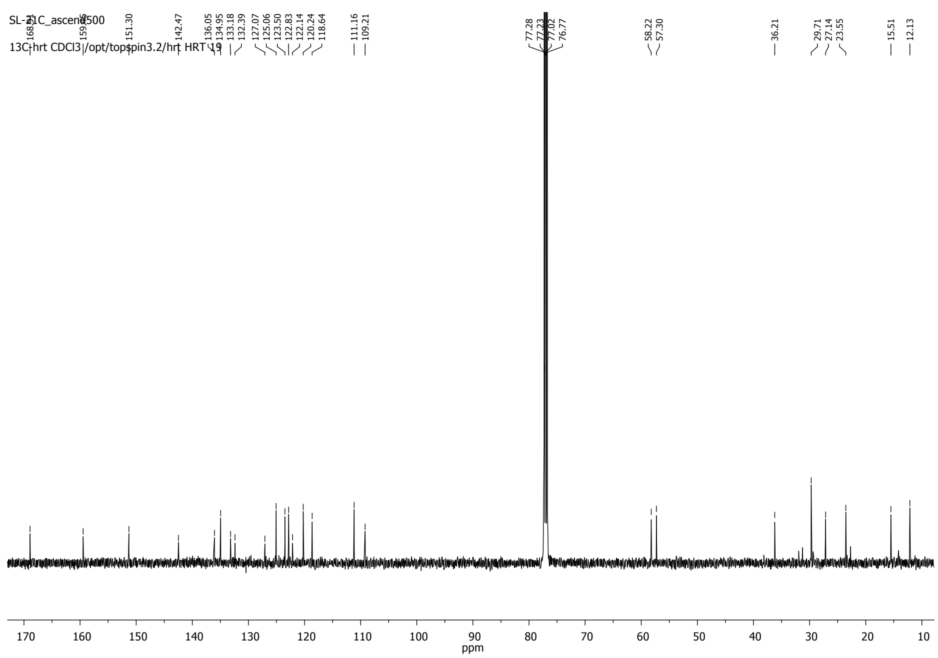

Fig. S17. ^{13}C NMR NMR spectrum of (1*S*,4*R*)-4-((1*H*-indol-3-yl)methyl)-1-((*S*)-sec-butyl)-8,10-dichloro-1,2-dihydro-6*H*-pyrazino[2,1-*b*]quinazoline-3,6(4*H*)-dione (**27**) ($CDCl_3$, 75, MHz).

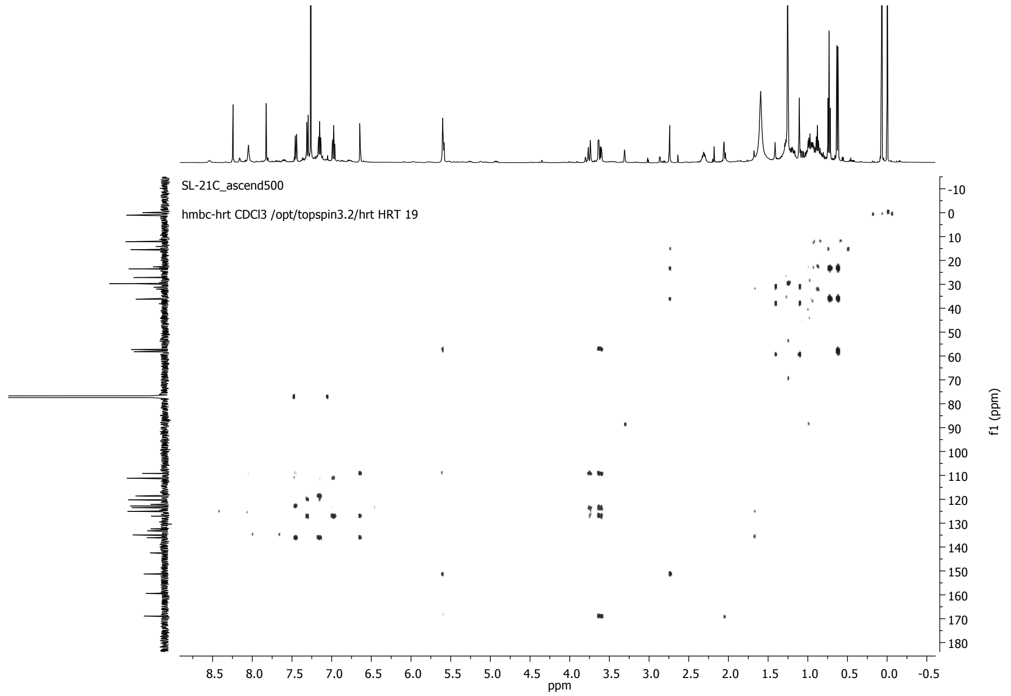

Fig. S18. HMBC spectrum of (1*S*,4*R*)-4-((1*H*-indol-3-yl)methyl)-1-((*S*)-sec-butyl)-8,10-dichloro-1,2-dihydro-6*H*-pyrazino[2,1-*b*]quinazoline-3,6(4*H*)-dione (**27**) ($CDCl_3$, 300, MHz).

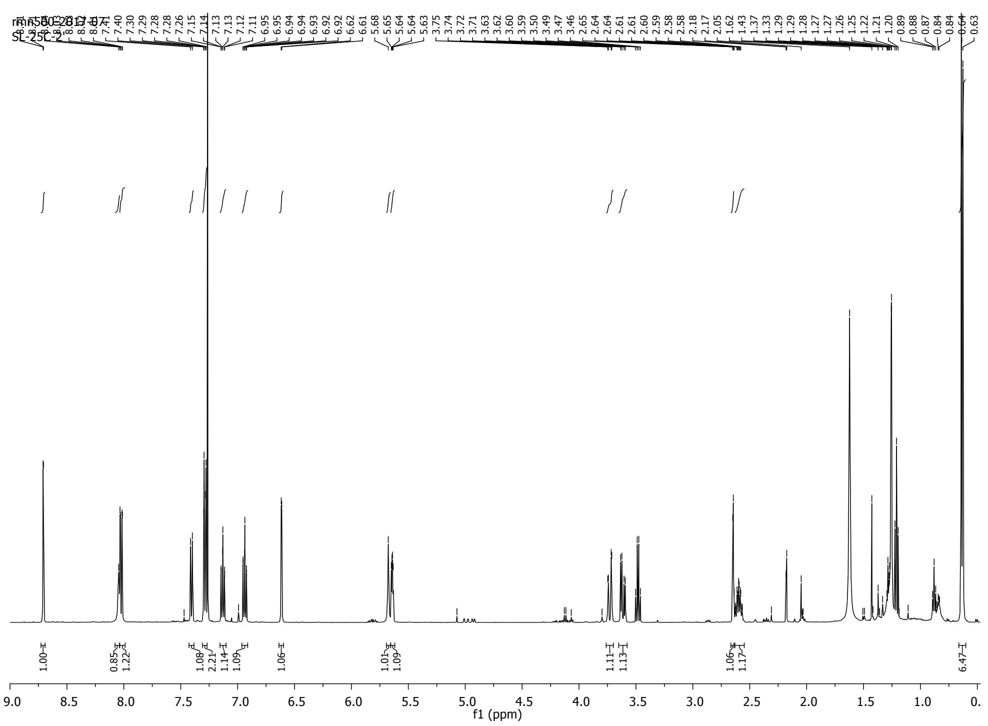

Fig. S19. ^{1}H NMR spectrum of (1*S*,4*R*)-4-((1*H*-indol-3-yl)methyl)-8-iodo-1-isopropyl-1,2-dihydro-6*H*-pyrazino[2,1-*b*]quinazoline-3,6(4*H*)-dione (**28**) ($CDCl_3$, 300, MHz).

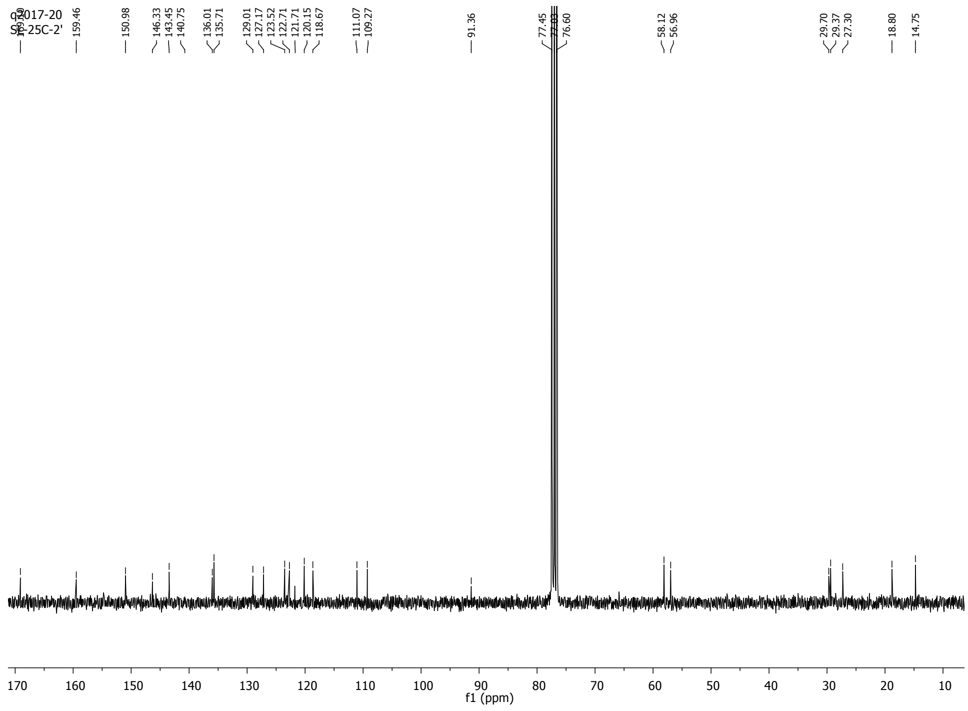

Fig. S20. ^{13}C NMR spectrum of (1*S*,4*R*)-4-((1*H*-indol-3-yl)methyl)-8-iodo-1-isopropyl-1,2-dihydro-6*H*-pyrazino[2,1-*b*]quinazoline-3,6(4*H*)-dione (**28**) ($CDCl_3$, 75, MHz).

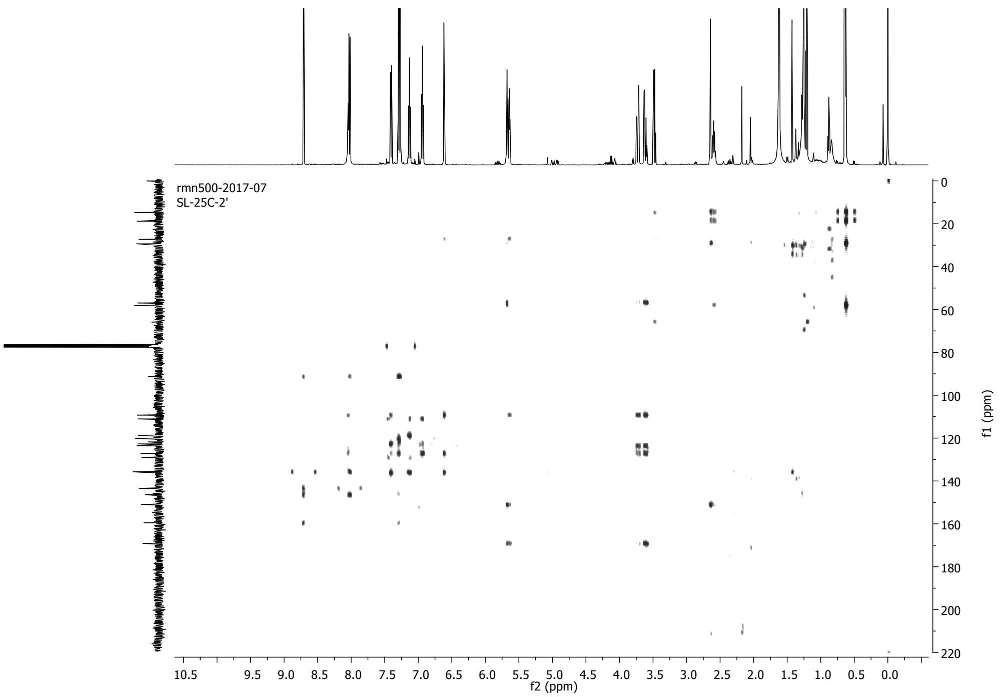

Fig. S21. HMBC spectrum of (1*S*,4*R*)-4-((1*H*-indol-3-yl)methyl)-8-iodo-1-isopropyl-1,2-dihydro-6*H*-pyrazino[2,1-*b*]quinazoline-3,6(4*H*)-dione (**28**) ($CDCl_3$, 300, MHz).

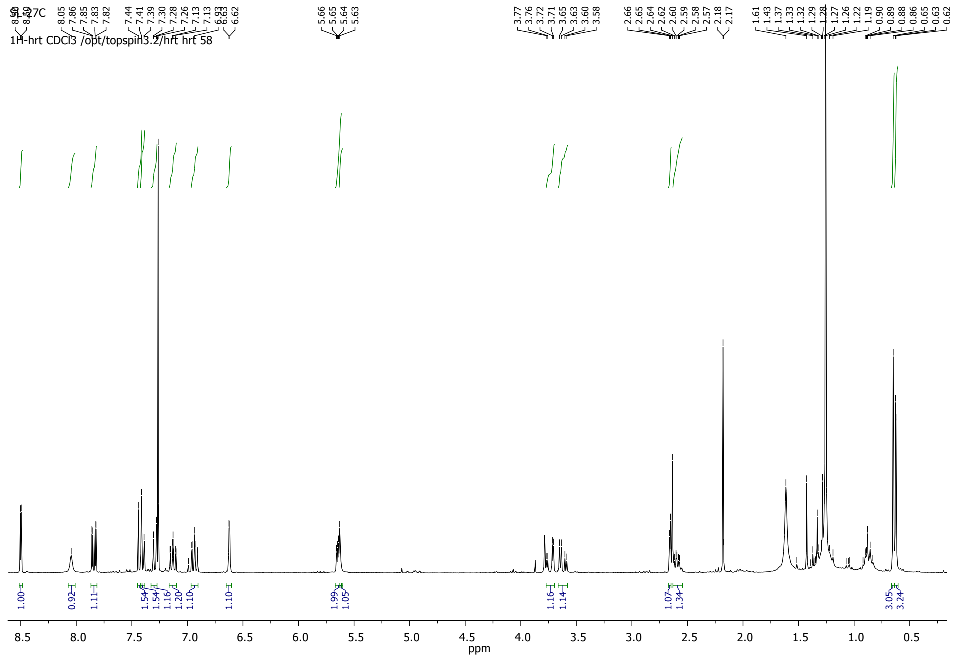

Fig. S22. ^{1}H NMR spectrum of (1*S*,4*R*)-4-((1*H*-indol-3-yl)methyl)-8-bromo-1-isopropyl-1,2-dihydro-6*H*-pyrazino[2,1-*b*]quinazoline-3,6(4*H*)-dione (**29**) ($CDCl_3$, 300, MHz).

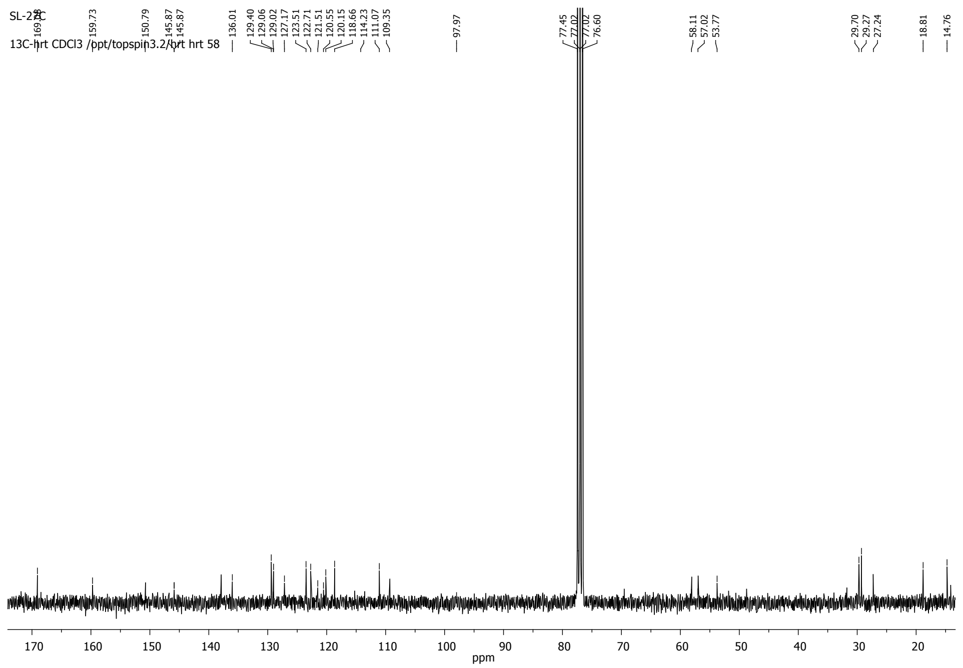

Fig. S23. ^{13}C NMR spectrum of (1*S*,4*R*)-4-((1*H*-indol-3-yl)methyl)-8-bromo-1-isopropyl-1,2-dihydro-6*H*-pyrazino[2,1-*b*]quinazoline-3,6(4*H*)-dione (**29**) ($CDCl_3$, 75, MHz).

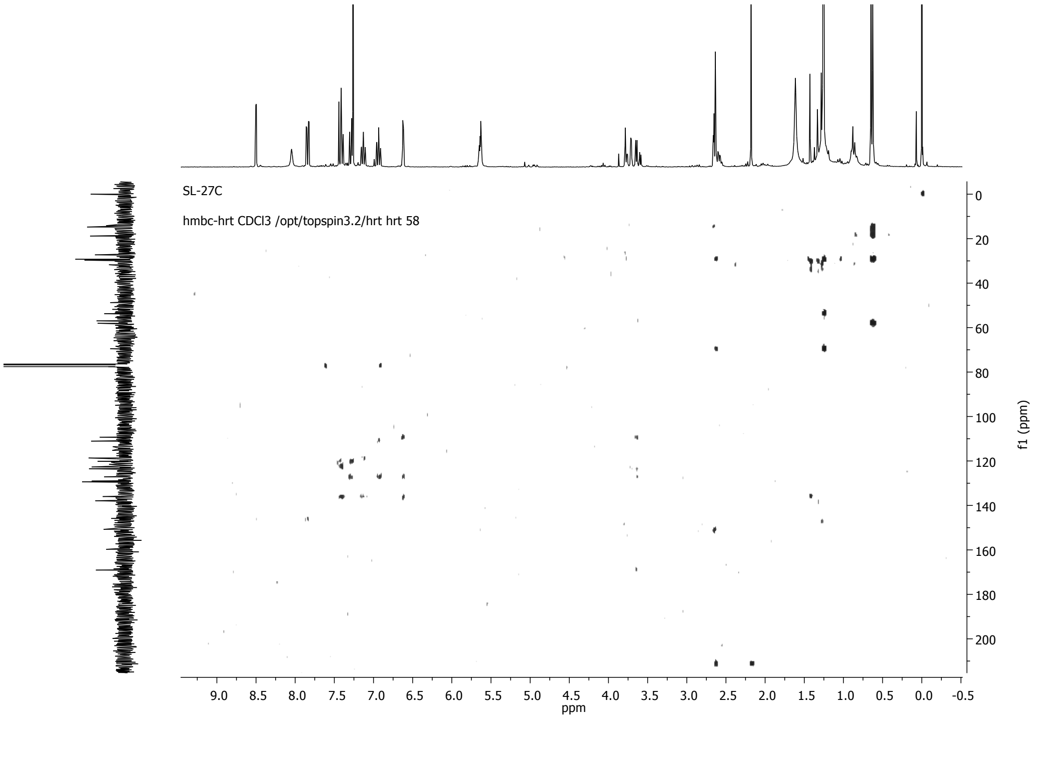

Fig. S24. HMBC spectrum of (1*S*,4*R*)-4-((1*H*-indol-3-yl)methyl)-8-bromo-1-isopropyl-1,2-dihydro-6*H*-pyrazino[2,1-*b*]quinazoline-3,6(4*H*)-dione (**29**) ($CDCl_3$, 300, MHz).

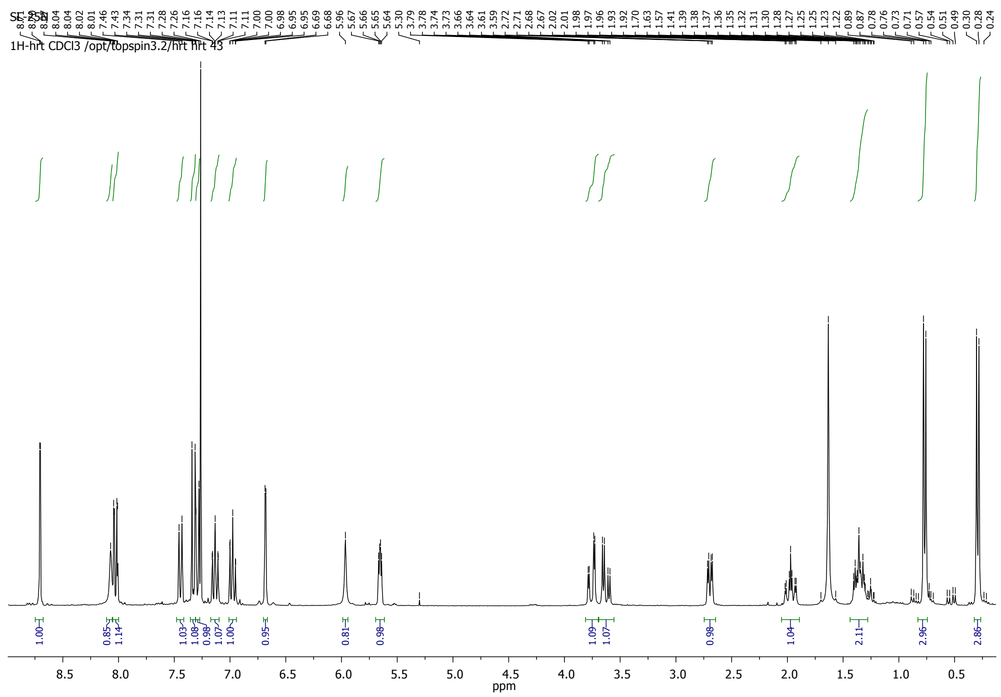

Fig. S25. ^{1}H NMR spectrum of (1*S*,4*R*)-4-((1*H*-indol-3-yl)methyl)-8-iodo-1-isobutyl-1,2-dihydro-6*H*-pyrazino[2,1-*b*]quinazoline-3,6(4*H*)-dione (**30**) ($CDCl_3$, 300, MHz).

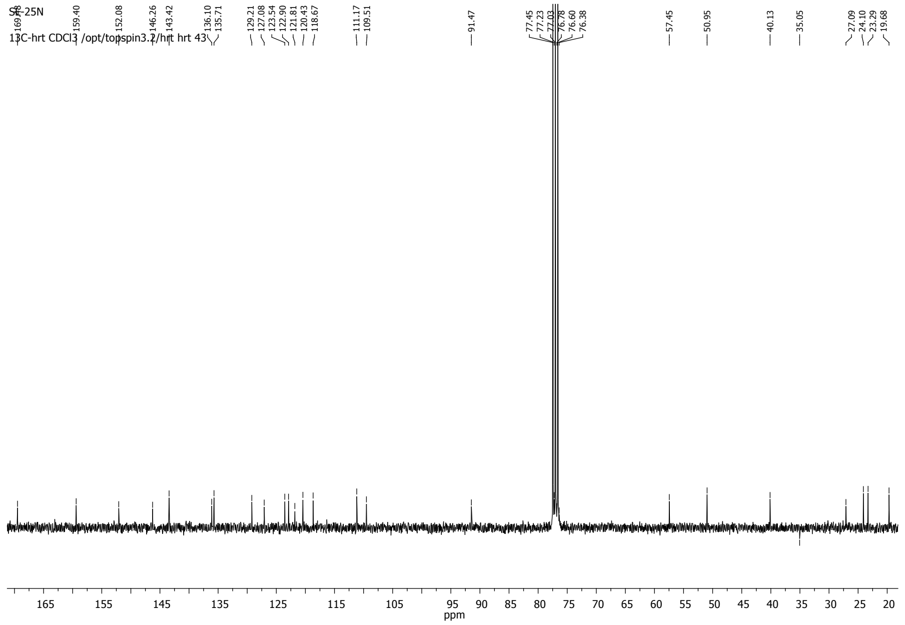

Fig. S26. ^{13}C NMR spectrum of (1*S*,4*R*)-4-((1*H*-indol-3-yl)methyl)-8-iodo-1-isobutyl-1,2-dihydro-6*H*-pyrazino[2,1-*b*]quinazoline-3,6(4*H*)-dione (**30**) ($CDCl_3$, 75, MHz).

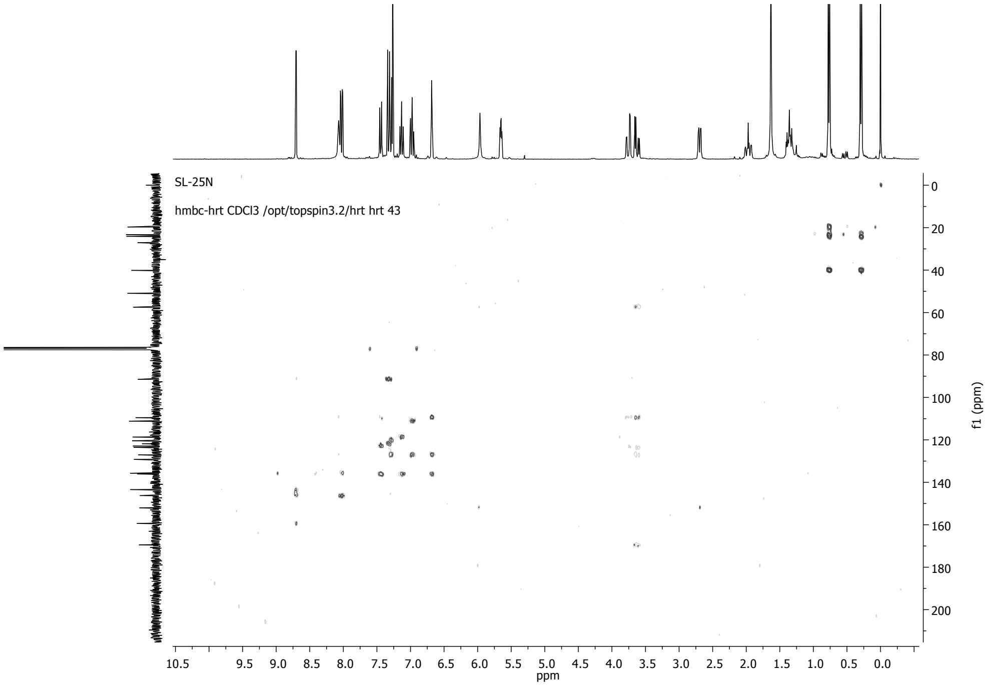

Fig. S27. HMBC spectrum of (1*S*,4*R*)-4-((1*H*-indol-3-yl)methyl)-8-iodo-1-isobutyl-1,2-dihydro-6*H*-pyrazino[2,1-*b*]quinazoline-3,6(4*H*)-dione (**30**) ($CDCl_3$, 300, MHz).

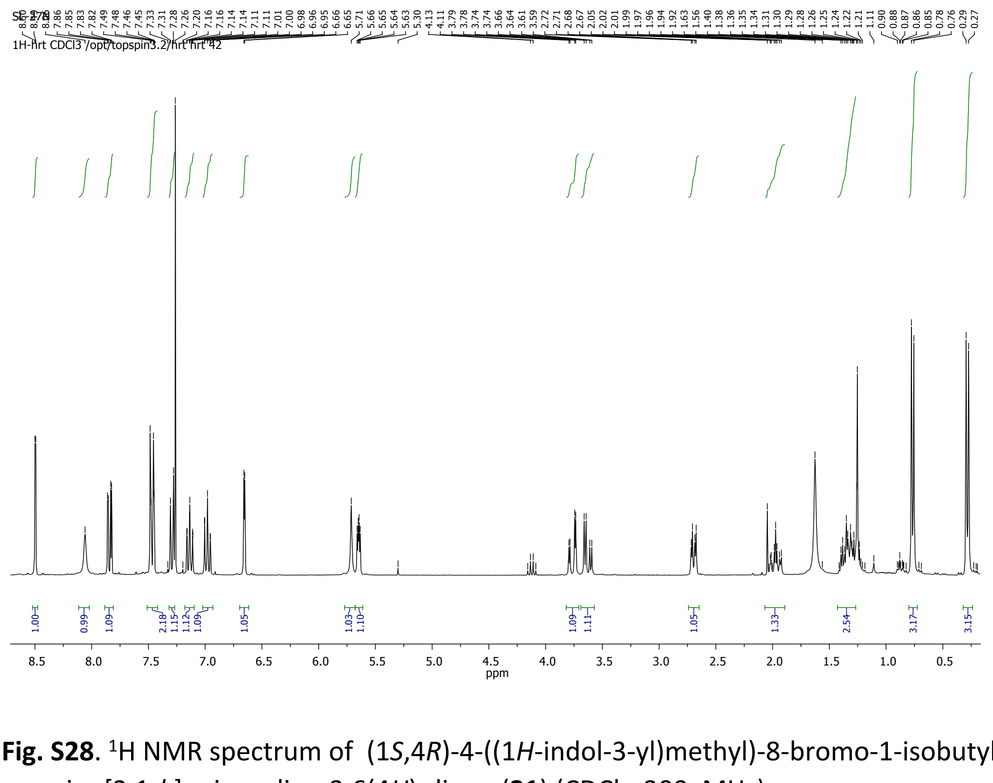

Fig. S28. ^{1}H NMR spectrum of (1*S*,4*R*)-4-((1*H*-indol-3-yl)methyl)-8-bromo-1-isobutyl-1,2-dihydro-6*H*-pyrazino[2,1-*b*]quinazoline-3,6(4*H*)-dione (**31**) ($CDCl_3$, 300, MHz).

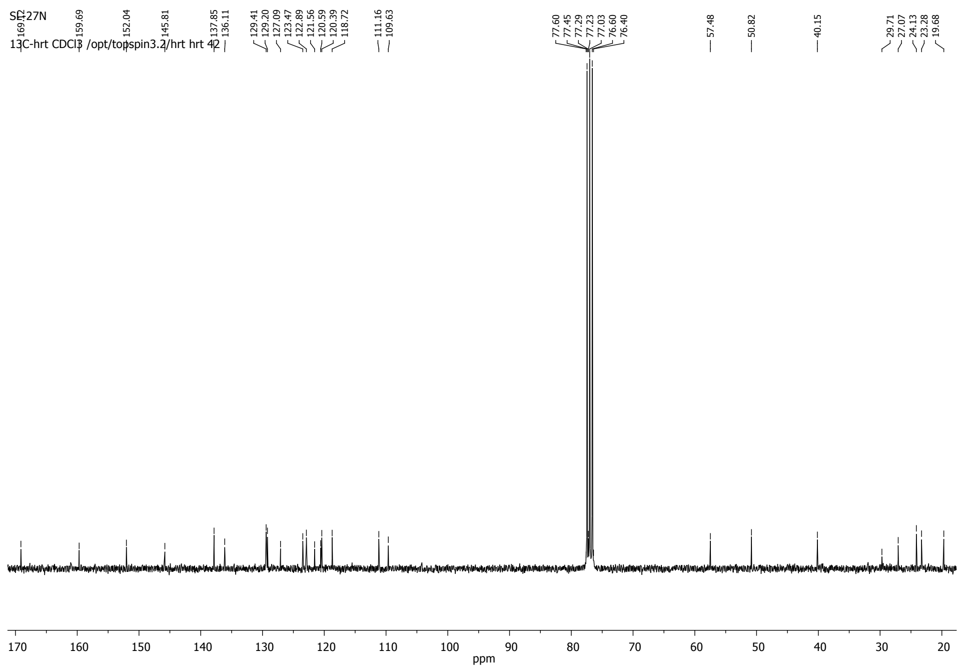

Fig. S29. ^{13}C NMR spectrum of (1*S*,4*R*)-4-((1*H*-indol-3-yl)methyl)-8-bromo-1-isobutyl-1,2-dihydro-6*H*-pyrazino[2,1-*b*]quinazoline-3,6(4*H*)-dione (**31**) ($CDCl_3$, 75, MHz).

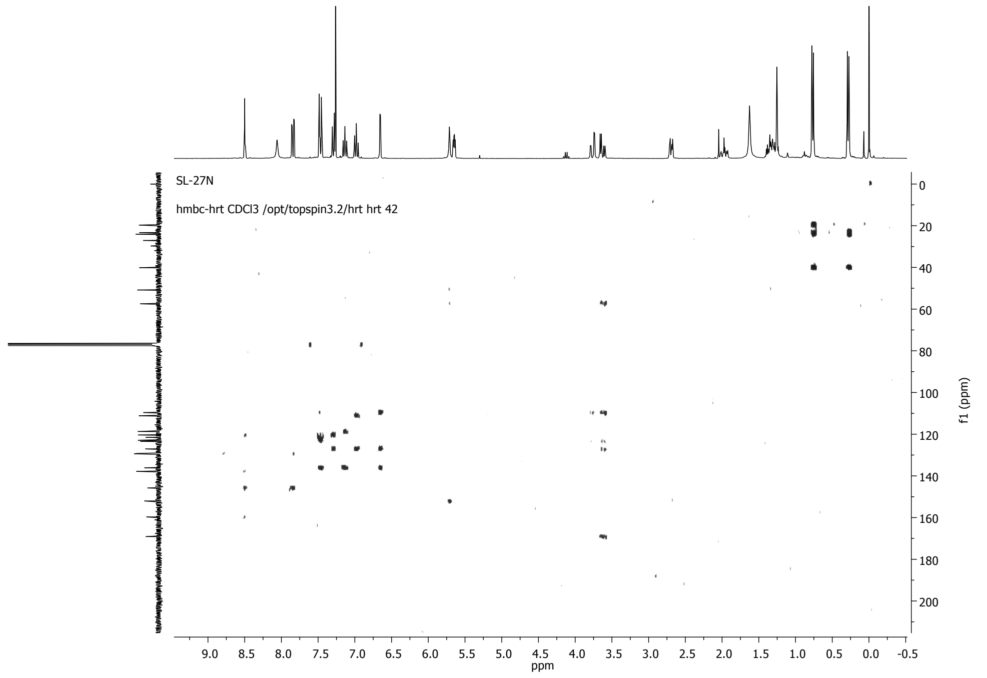

Fig. S30. ^{1}H NMR spectrum of (1*S*,4*R*)-4-((1*H*-indol-3-yl)methyl)-8-bromo-1-isobutyl-1,2-dihydro-6*H*-pyrazino[2,1-*b*]quinazoline-3,6(4*H*)-dione (**31**) (DMSO-d_6, 300, MHz).

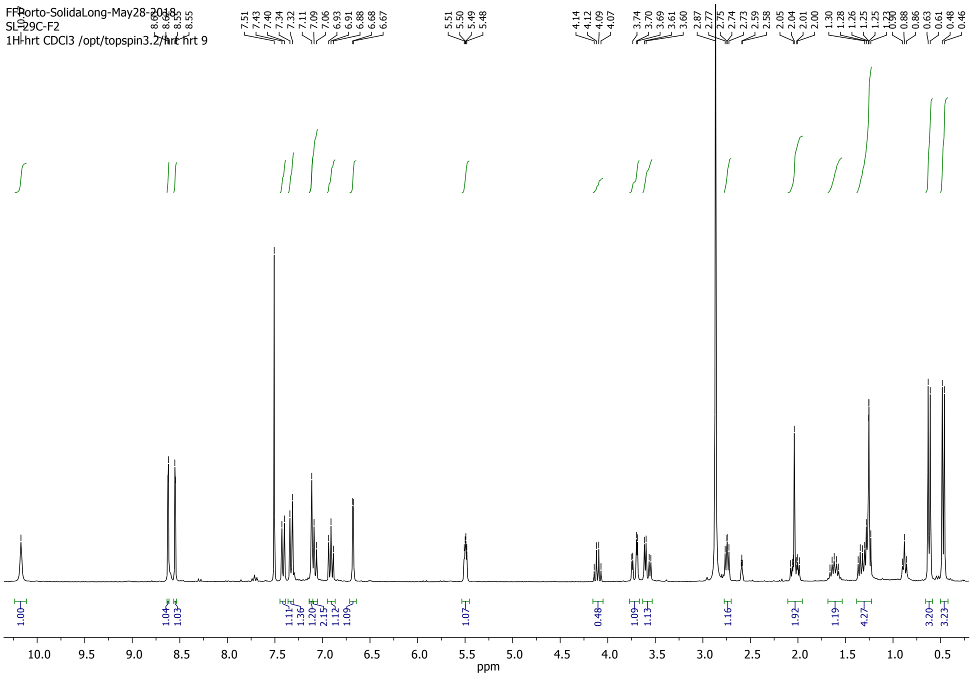

Fig. S31. ^{1}H NMR spectrum of (1*S*,4*R*)-4-((1*H*-indol-3-yl)methyl)-8,10-diiodo-1-isobutyl-1,2-dihydro-6*H*-pyrazino[2,1-*b*]quinazoline-3,6(4*H*)-dione (**32**) (DMSO-d_6, 300, MHz).

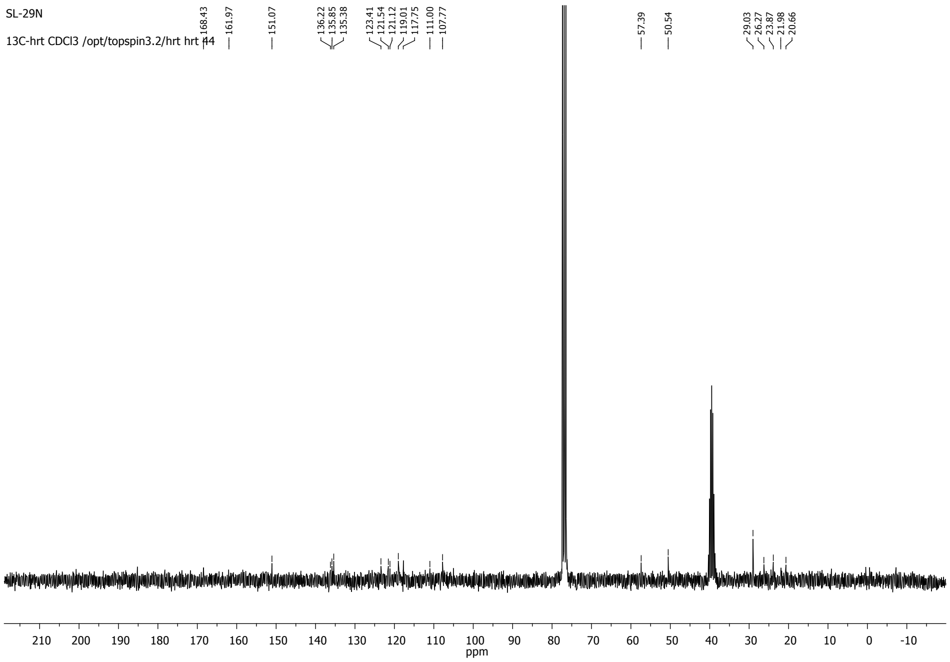

Fig. S32. ^{13}C NMR spectrum of (1*S*,4*R*)-4-((1*H*-indol-3-yl)methyl)-8,10-diiodo-1-isobutyl-1,2-dihydro-6*H*-pyrazino[2,1-*b*]quinazoline-3,6(4*H*)-dione (**32**) (DMSO-d_6, 75, MHz).

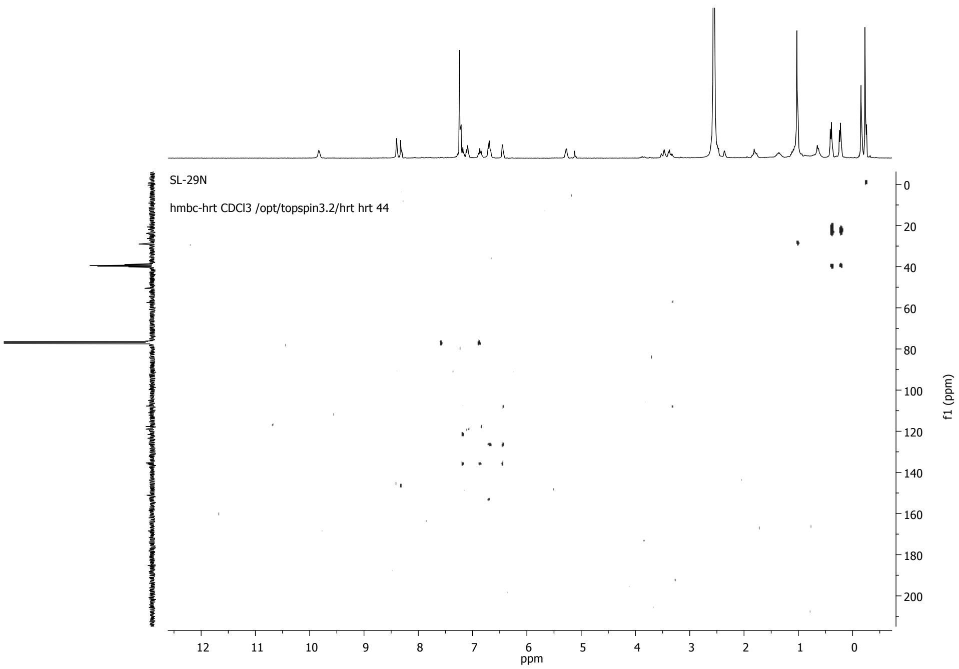

Fig. S33. HMBC spectrum of (1*S*,4*R*)-4-((1*H*-indol-3-yl)methyl)-8,10-diiodo-1-isobutyl-1,2-dihydro-6*H*-pyrazino[2,1-*b*]quinazoline-3,6(4*H*)-dione (**32**) (DMSO-d_6, 300, MHz).

2. Enantioselective liquid chromatography

2.1 Chiral analysis of compounds 22-32

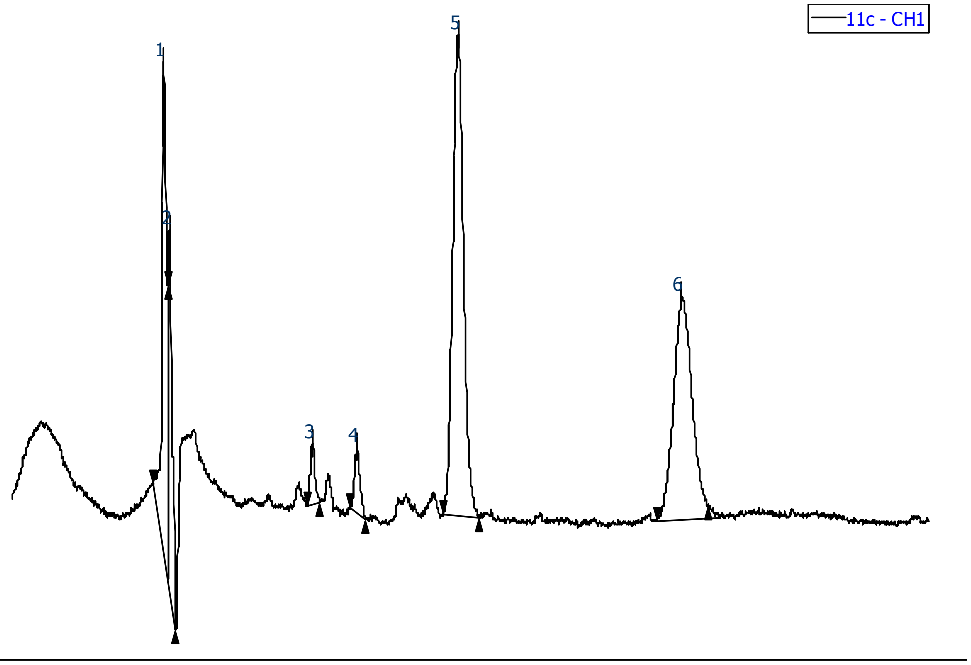

Fig. S34. Chiral analysis of (1*S*,4*R*)-4-((1*H*-indol-3-yl)methyl)-8-chloro-1-isopropyl-1,2-dihydro-6*H*-pyrazino[2,1-b]quinazoline-3,6(4*H*)-dione (**22**), Mobile phase: Hexane:MeOH, 90:10; flow rate: 0.5 mL/min.

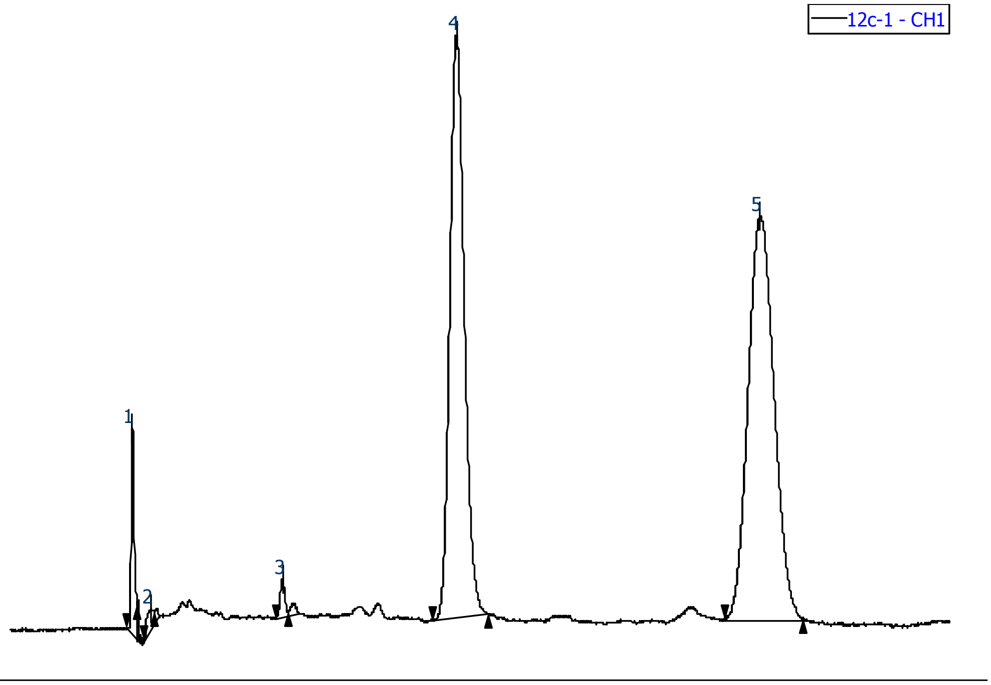

Fig. S35. Chiral analysis of (1*S*,4*R*)-4-((1*H*-indol-3-yl)methyl)-8-chloro-1-isobutyl-1,2-dihydro-6*H*-pyrazino[2,1-b]quinazoline-3,6(4*H*)-dione (**23**), Mobile phase: Hexane:MeOH, 90:10; flow rate: 0.5 mL/min.

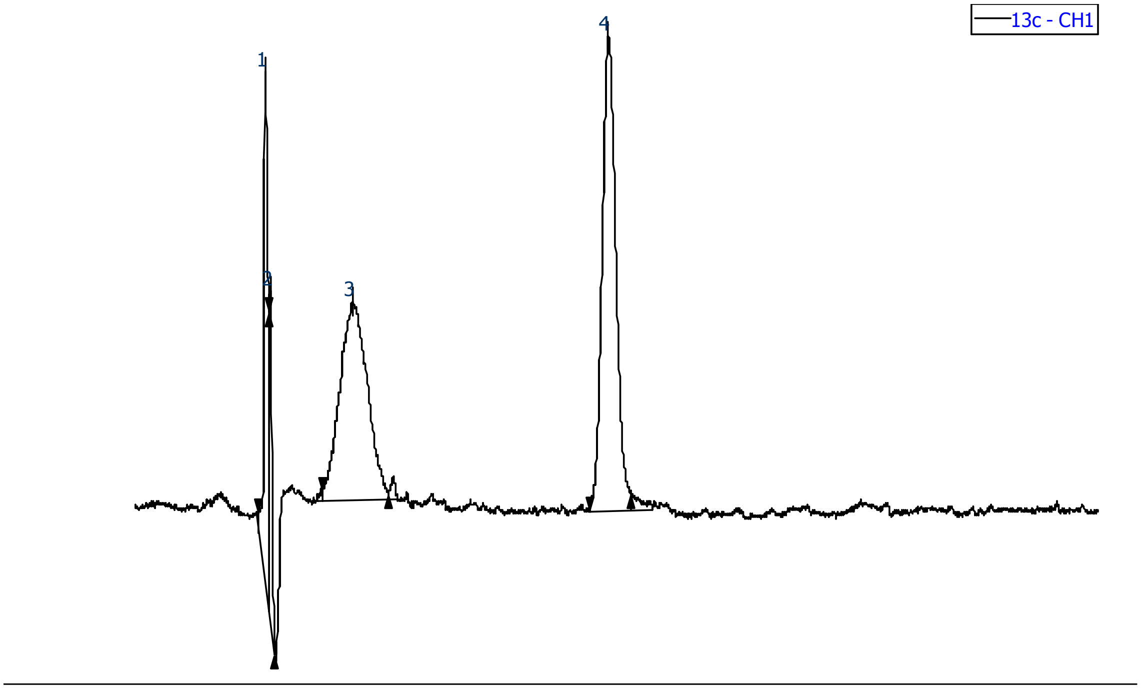

Fig. S36. Chiral analysis of (1*S*,4*R*)-4-((1*H*-indol-3-yl)methyl)-1-((*S*)-sec-butyl)-8-chloro-1,2-dihydro-6*H*-pyrazino[2,1-*b*]quinazoline-3,6(4*H*)-dione (**24**), Mobile phase: Hexane:MeOH, 90:10; flow rate: 0.5 mL/min.

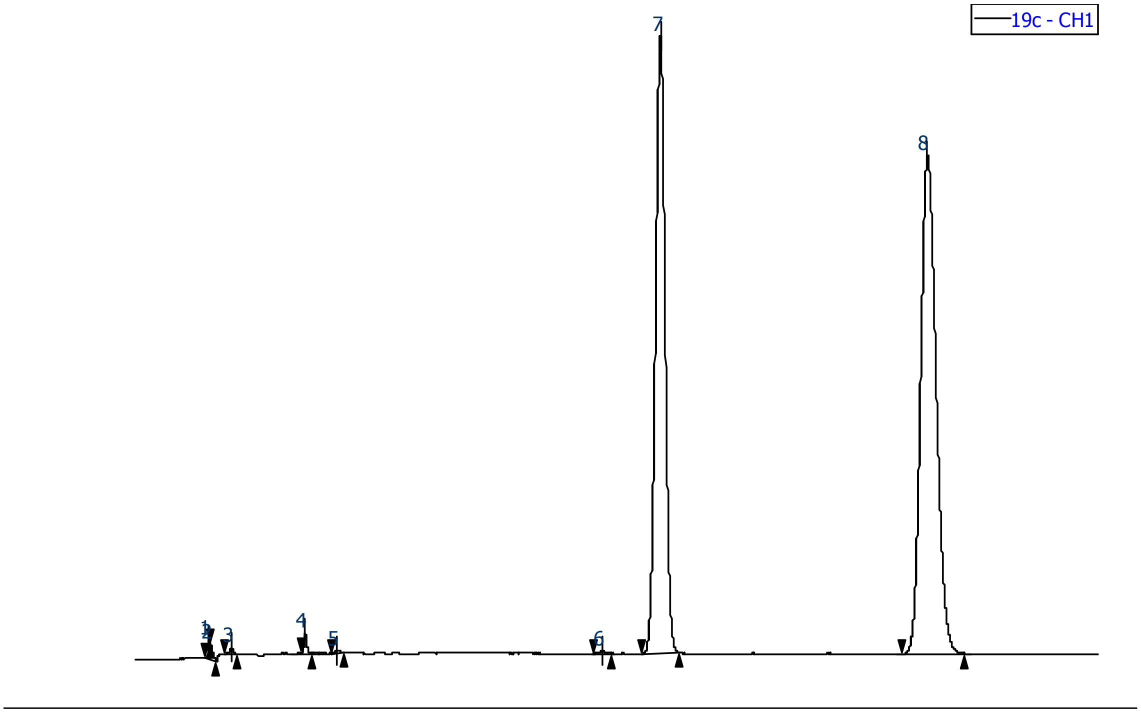

Fig. S37. Chiral analysis of (1*S*,4*R*)-4-((1*H*-indol-3-yl)methyl)-8,10-dichloro-1-isopropyl-1,2-dihydro-6*H*-pyrazino[2,1-*b*]quinazoline-3,6(4*H*)-dione (**25**), Solvent: Hexan:MeOH, 90:10; flow rate: 0.5 mL/min.

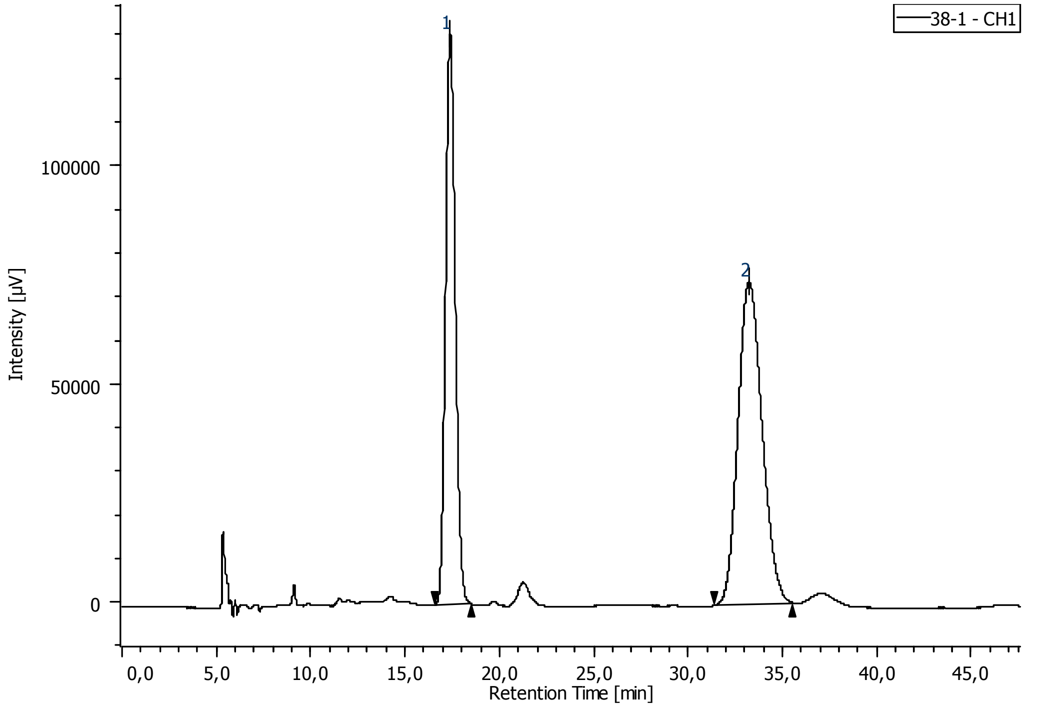

Fig. S38. Chiral analysis of (1*S*,4*R*)-4-((1*H*-indol-3-yl)methyl)-8,10-dichloro-1-isobutyl-1,2-dihydro-6*H*-pyrazino[2,1-*b*]quinazoline-3,6(4*H*)-dione (**26**), Mobile phase: Hexane:MeOH, 90:10; flow rate: 0.5 mL/min.

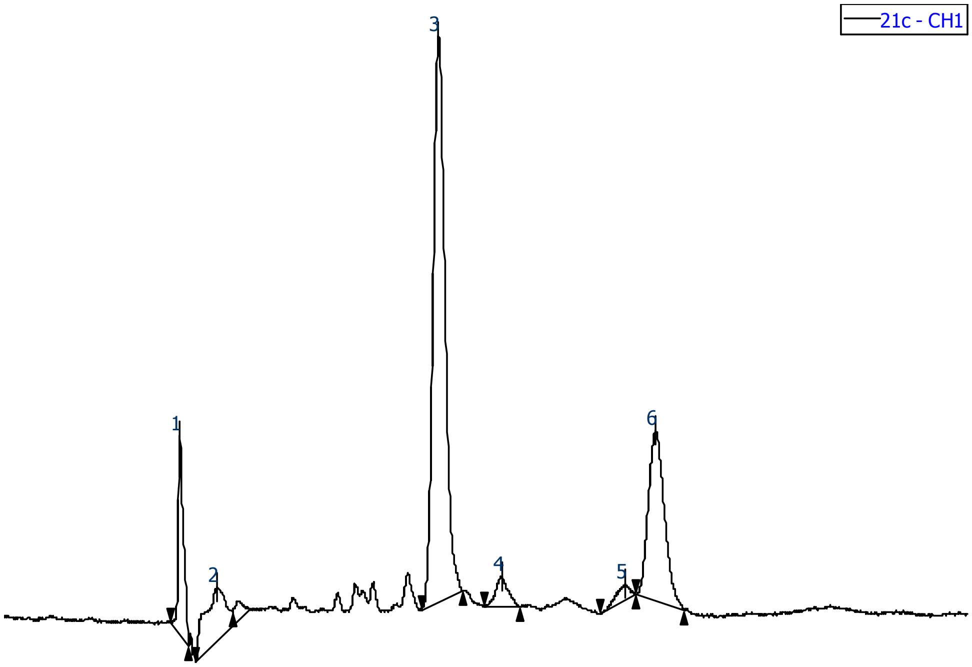

Fig. S39. Chiral analysis of (1*S*,4*R*)-4-((1*H*-indol-3-yl)methyl)-8,10-dichloro-1-((*S*)-sec-butly)-1,2-dihydro-6*H*-pyrazino[2,1-*b*]quinazoline-3,6(4*H*)-dione (**27**), Mobile phase: Hexane:MeOH, 90:10; flowrate: 0.5 mL/min.

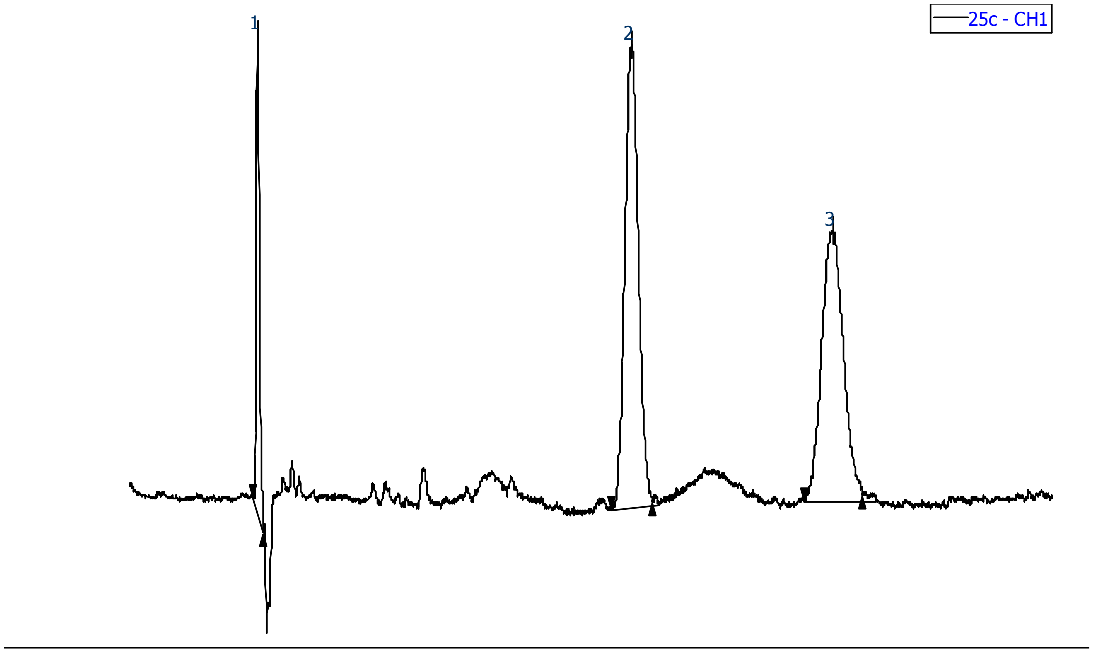

Fig. S40. Chiral analysis of (1*S*,4*R*)-4-((1*H*-indol-3-yl)methyl)-8-iodo-1-isopropyl-1,2-dihydro-6*H*-pyrazino[2,1-b]quinazoline-3,6(4*H*)-dione (**28**), Mobile phase: Hexane:MeOH, 90:10; flow rate: 0.5 mL/min.

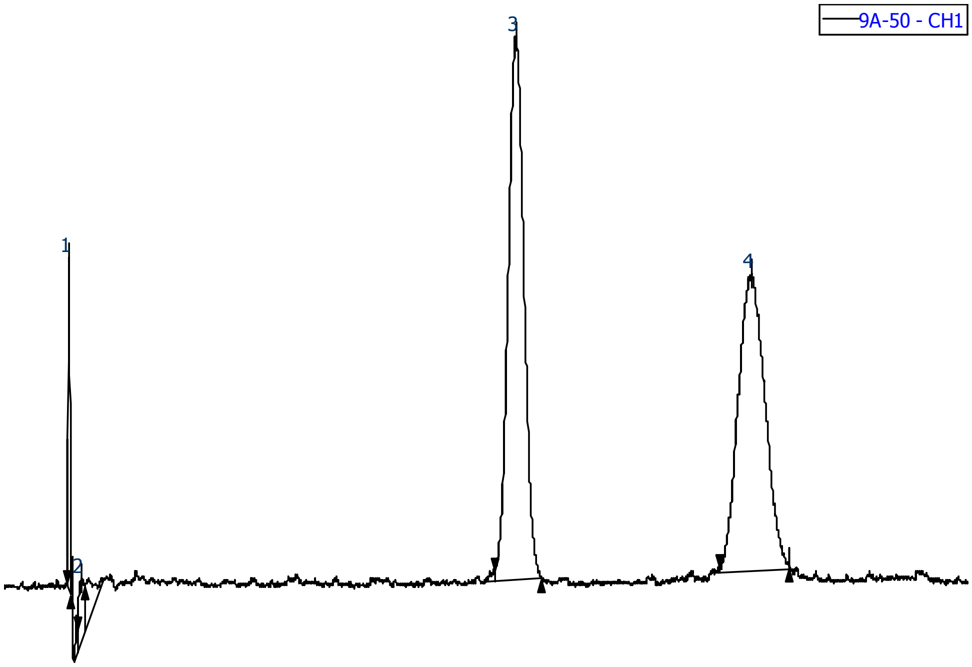

Fig. S41. Chiral analysis of (1*S*,4*R*)-4-((1*H*-indol-3-yl)methyl)-8-bromo-1-isopropyl-1,2-dihydro-6*H*-pyrazino[2,1-b]quinazoline-3,6(4*H*)-dione (**29**), Mobile phase: Hexane:MeOH, 90:10; flow rate: 0.5 mL/min.

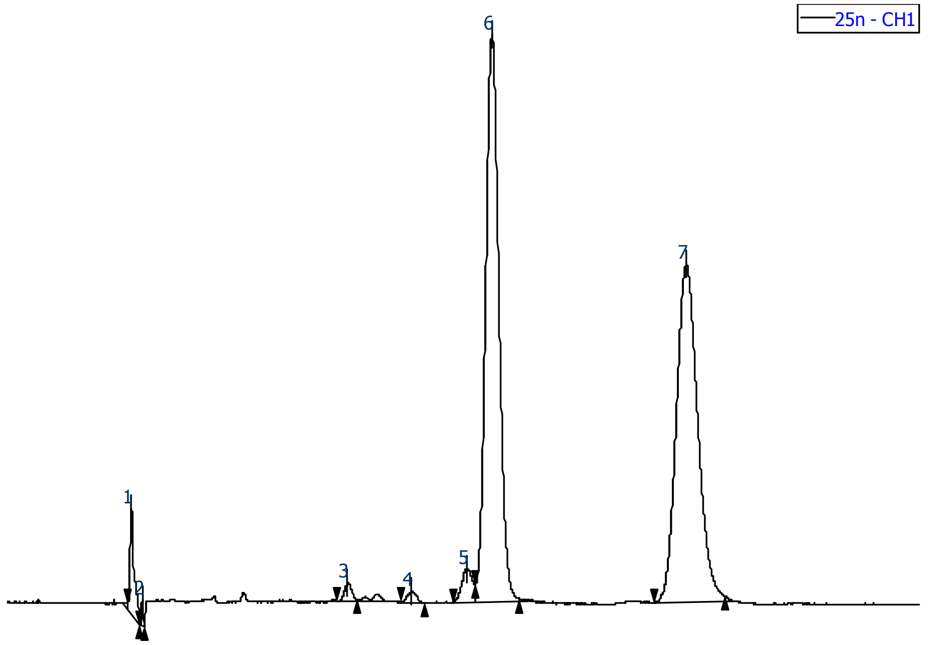

Fig. S42. Chiral analysis of (1*S*,4*R*)-4-((1*H*-indol-3-yl)methyl)-8-iodo-1-isobutyl-1,2-dihydro-6*H*-pyrazino[2,1-b]quinazoline-3,6(4*H*)-dione (**30**), Mobile phase: Hexane:MeOH, 90:10; flow rate: 0.5 mL/min.

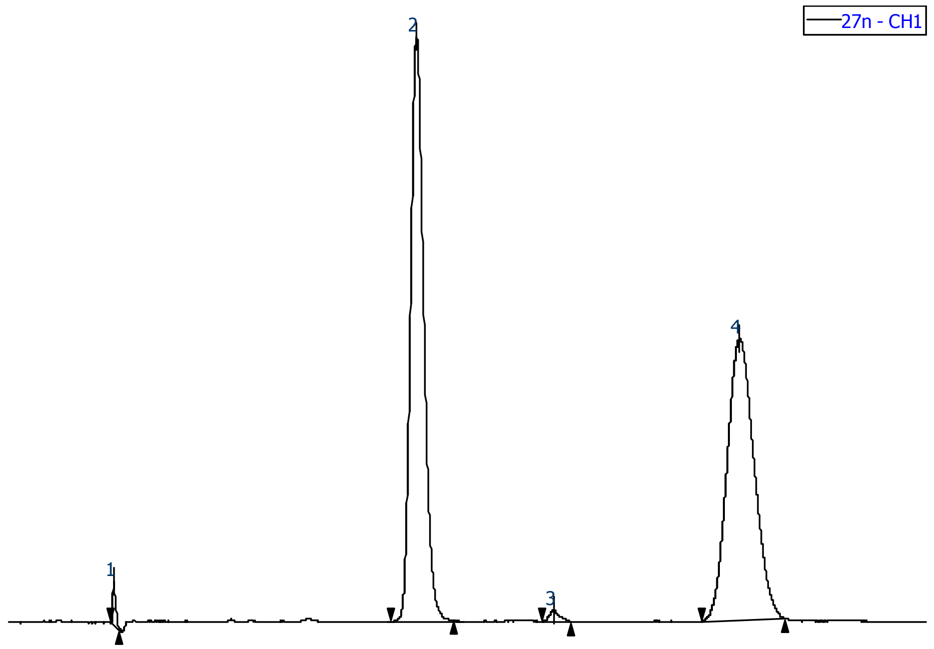

Fig. S43. Chiral analysis of (1*S*,4*R*)-4-((1*H*-indol-3-yl)methyl)-8-bromo-1-isobutyl-1,2-dihydro-6*H*-pyrazino[2,1-b]quinazoline-3,6(4*H*)-dione (**31**), Mobile phase: Hexane:MeOH, 90:10; flow rate: 0.5 mL/min.

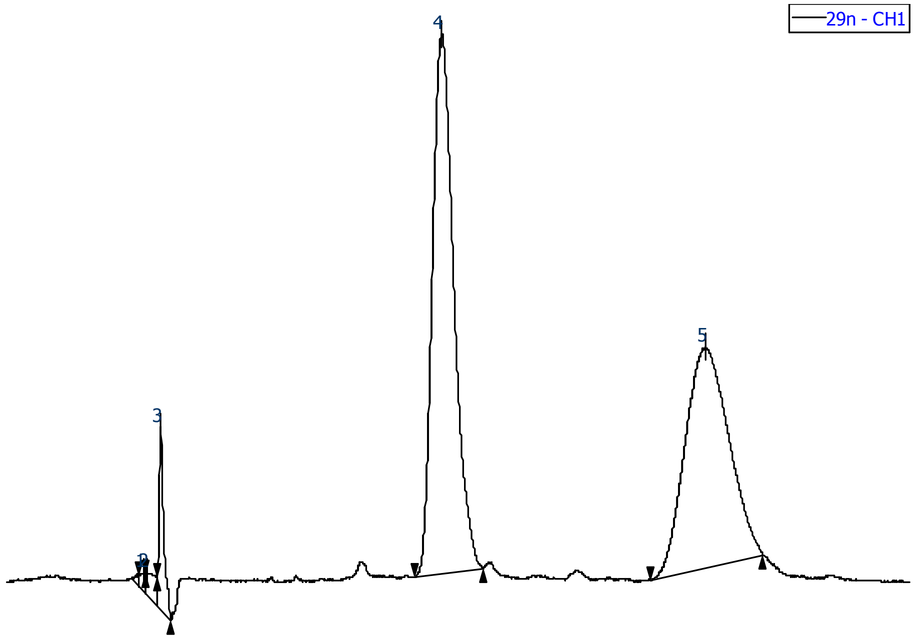

Fig. S44. Chiral analysis of (1*S*,4*R*)-4-((1*H*-indol-3-yl)methyl)-8,10-diiodo-1-isobutyl-1,2-dihydro-6*H*-pyrazino[2,1-*b*]quinazoline-3,6(4*H*)-dione (**32**), Mobile phase: Hexan:MeOH, 90:10; flowrate: 0.5 mL/min.

2.2 Peak purity analyzed on Reversed-Phase HPLC of compounds 22, 23, and 26

The peak purities of most promising compounds were analyzed using reversed-phase liquid chromatography separation employed FortisBIO C18 Column (250 x 4.6 mm, Part number: BIO315-050905), the mobile phase was MeCN/MeOH 50:50, running time: 30 min, minimum wavelength: 210 nm, maximum wavelength: 800 nm. The purity view properties: wavelength range: 225-800 nm, scan threshold: 5 mAU, peak coverage: 95%.

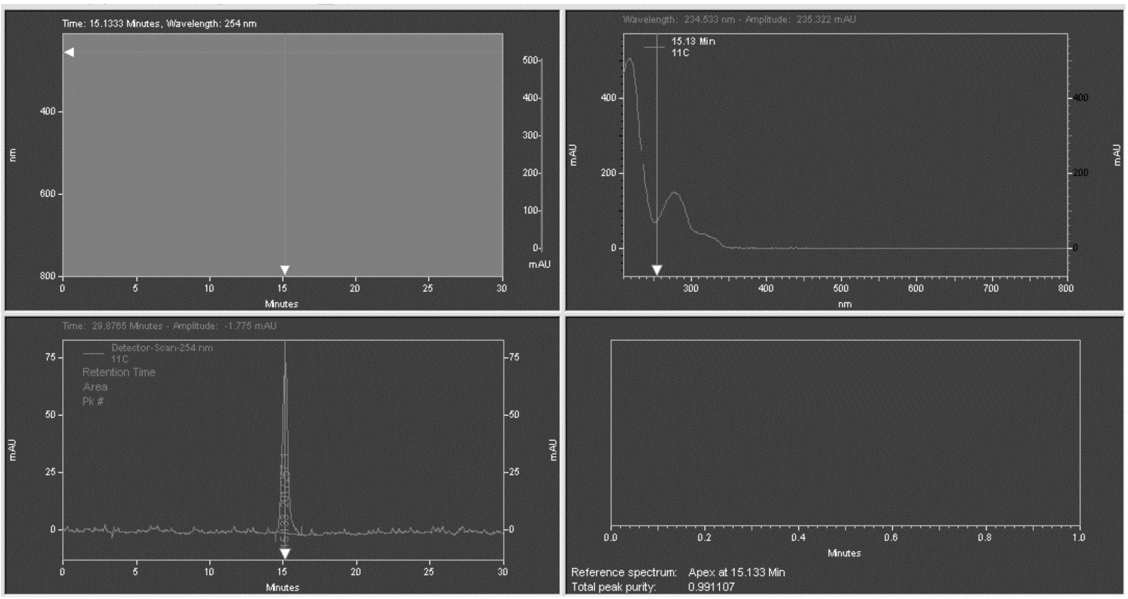

Fig. S45. Peak purity of (1*S*,4*R*)-4-((1*H*-indol-3-yl)methyl)-8-chloro-1-isopropyl-1,2-dihydro-6*H*-pyrazino[2,1-b]quinazoline-3,6(4*H*)-dione (**22**).

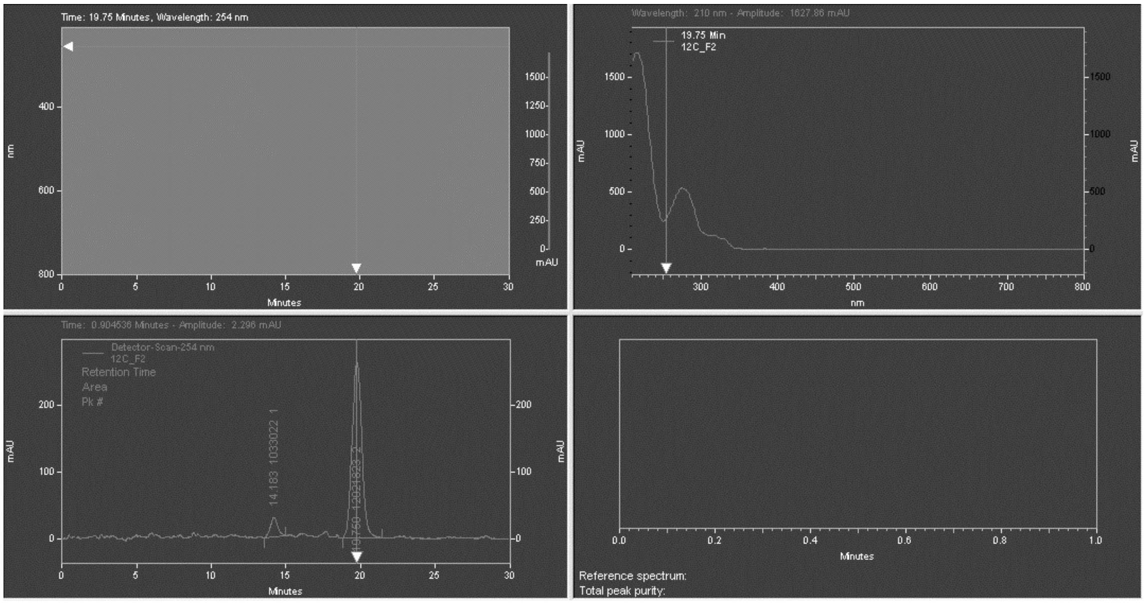

Fig. S46. Peak purity of (1*S*,4*R*)-4-((1*H*-indol-3-yl)methyl)-8-chloro-1-isobutyl-1,2-dihydro-6*H*-pyrazino[2,1-b]quinazoline-3,6(4*H*)-dione (**23**).

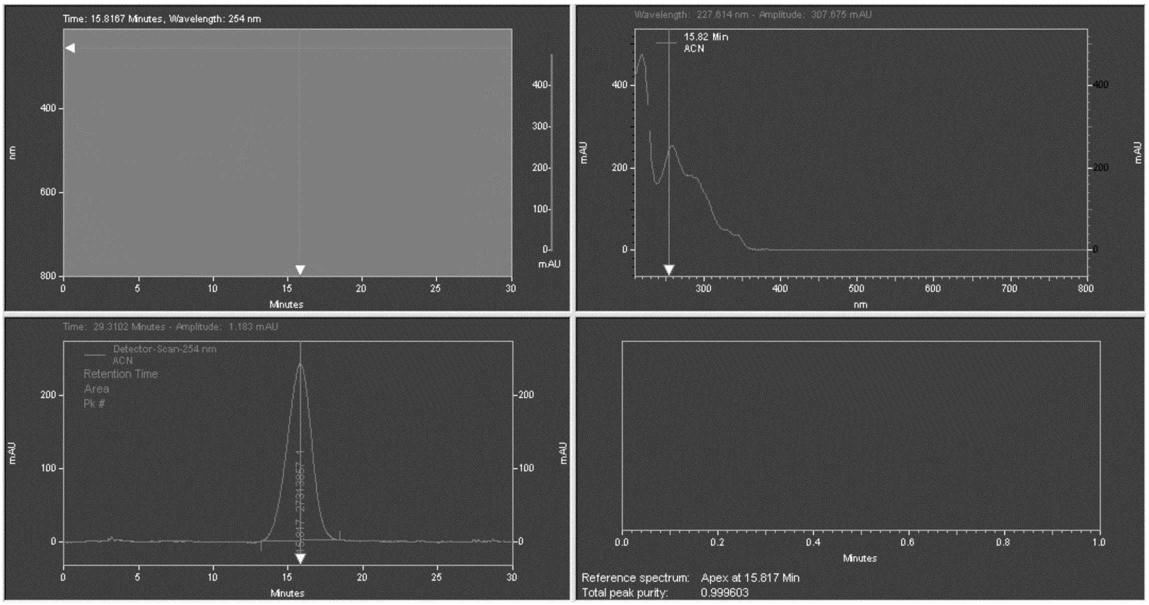

Fig. S47. Peak purity of (1*S*,4*R*)-4-((1*H*-indol-3-yl)methyl)-8,10-dichloro-1-isobutyl-1,2-dihydro-6*H*-pyrazino[2,1-*b*]quinazoline-3,6(4*H*)-dione (**26**).

2.3 Enantioselective liquid chromatography separation of compounds 22, 23, and 26

Enantioselective liquid chromatography. In order to evaluate enantioselectivity in vitro activities, such as antibacterial and antifungal, the most promising derivatives **22**, **23**, and **26**, were obtained in milligram scale by semipreparative enantioselective liquid chromatography, employing a tris-3,5-dimethylphenylcarbamate amylose column with multiple injection in a 200 µL loop. Initial conditions were investigated with analytical Pirkle columns in polar mode [Whelk-O1-S,S (250 × 4.6 mm) and UV-detection at 245 nm, mobile phase MeOH/MeCN 50:50, flow rate 1.0 mL/min] and polysaccharide based columns (amylose and cellulose) in multimodal conditions. The best analytical conditions [Amylose-1, (250 × 4.6 mm), mobile phase hexane:EtOH, 90:10, flow rate 0.5 mL/min] presented good separation ($\alpha > 1.2$) and resolution values ($Rs > 8$) for all compounds to allow the scale-up to the preparative mode. The semipreparative separation was optimized by adjusting the sample volume from the analytical method. The optimized mobile phase of analytical system (hexane:EtOH, 90:10) was transferred without any modification to semipreparative mode and 254 was chosen as minimum wavelength absorption. The column diameter was enlarged to a scale-up factor of 3. The flow rate was increase from 0.5 to 2 mL/min, and the retention times were between 15 to 50 min. The loading effect in semipreparative mode was examined by keeping the concentration of the feed solution at the maximum (1.5 mg/mL) and by varying the volume (100 to 200 µL). The mobile phase composition, chromatograms, and chromatographic parameters (Tables S1 and S2) at analytical and semipreparative scales.

Table S1. Separation performance on the amylose tris-3,5-dimethylphenylcarbamate phase for compounds **22**, **23**, and **26**.

Compounds structure	Analytical column [a]	Semipreparative column [b]
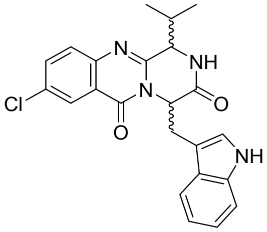 **22**	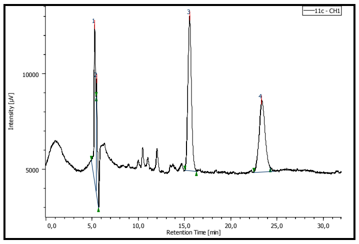 (k_1 = 1.95, α = 1.76 *Rs* = 8.43)	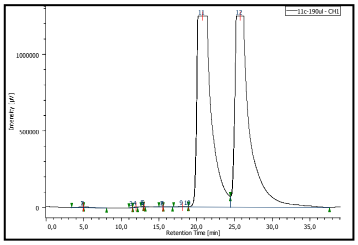
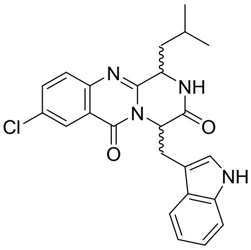 **23**	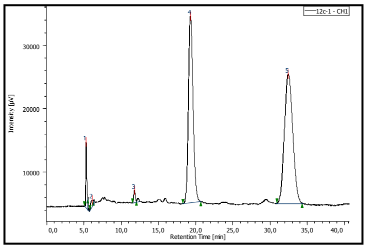 (k_1 = 2.64, α = 1.94 *Rs* = 8.15)	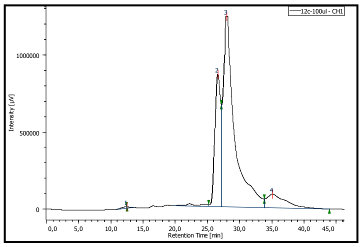
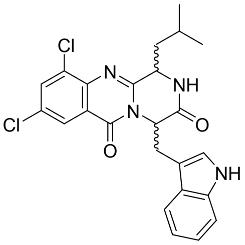 **26**	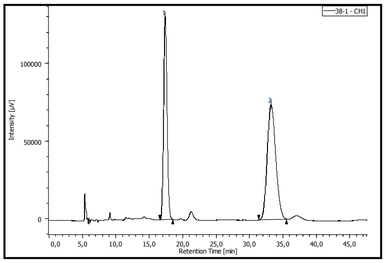 (k_1 = 5.60, α = 1.75 *Rs* = 11.69)	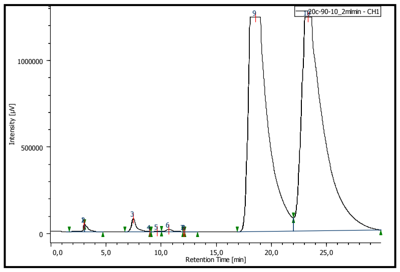

[a] Flow rate: 0.5 mL/min, loop 20 µL, detection: 254 nm, column: Lux® 5 µm Amylose-1, (250 × 4.6 mm), mobile phase hexane:EtOH, 90:10. k: retention factor, α: enantioselective selectivity, Rs: resolution index. [b] Flow rate: 2 mL/min, loop 200 µL, loading ca. 1.5 mg/mL in hexane:EtOH (50:50), detection 254 nm, column: amylose tris-3,5-dimethylphenylcarbamate coated with Nucleosil (200 mm × 7 mm); mobile phase hexane:EtOH, 90:10.

Table S2. Elution order, specific rotation, and enantiomeric excess (*e.r*) of the resolved compound **22**, **23**, and **26** enantiomers.

Enantiomer	Elution order	[α]D (c)[a]	*e.r* (%)[b]
**(-)-22**	First order	-0.06 (0.08)	>99:1
**(+)-22**	Second order	+0.04 (0.10)	>99:1
**(-)-23**	First order	-0.08 (0.05)	>99:1
**(+)-23**	Second order	+0.22 (0.12)	>99:1
**(-)-26**	First order	-0.16 (0.03)	97:3
**(+)-26**	Second order	+0.15 (0.03)	>99:1

[a] Specific rotation in MeOH with c = concentration in g/mL. [b] Enantiomeric ratio (e.r) determinated by enantioselective LC described in experimental conditions. Used as an expression of enantiomer purity, this ratio was normalized as a percent.

3. High resolution mass spectra

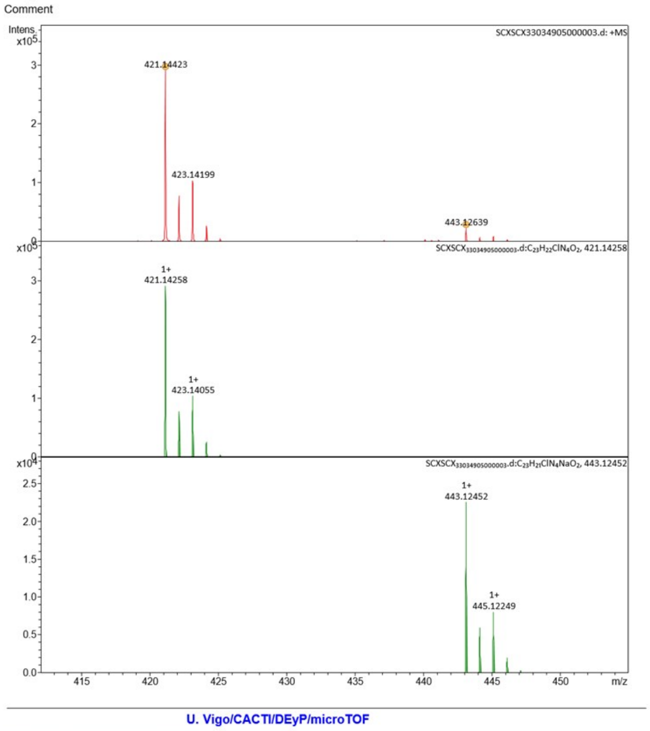

Fig. S48: Mass spectra of (1*S*,4*R*)-4-((1*H*-indol-3-yl)methyl)-8-chloro-1-isopropyl-1,2-dihydro-6*H*-pyrazino[2,1-b]quinazoline-3,6(4*H*)-dione (**22**). (20, 300v)

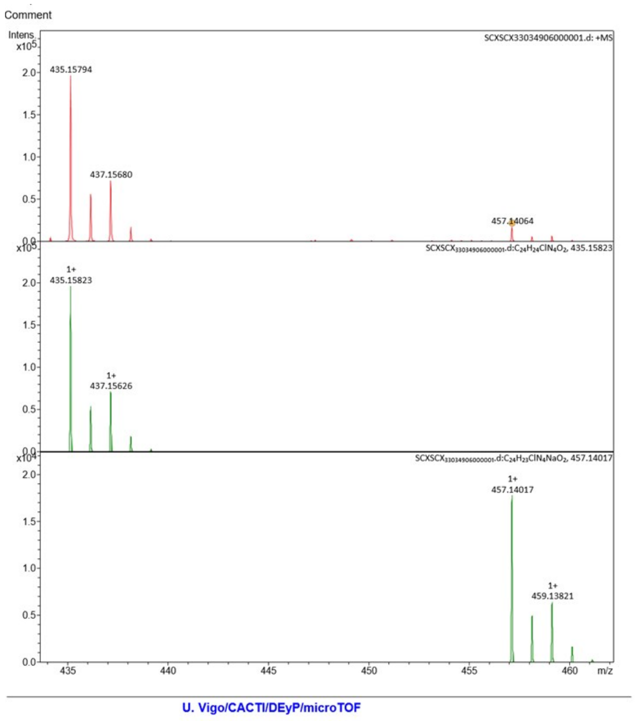

Fig. S49: Mass spectra of (1*S*,4*R*)-4-((1*H*-indol-3-yl)methyl)-8-chloro-1-isobutyl-1,2-dihydro-6*H*-pyrazino[2,1-b]quinazoline-3,6(4*H*)-dione (**23**). (20, 300v)

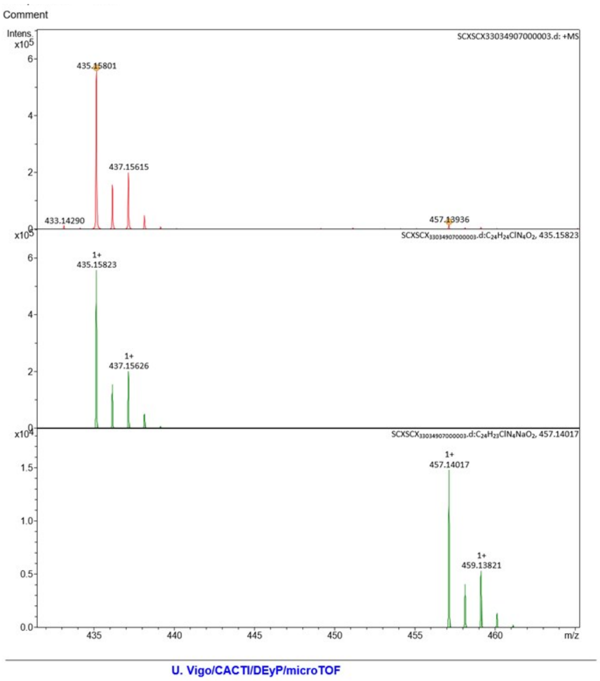

Fig. S50: Mass spectra of (1*S*,4*R*)-4-((1*H*-indol-3-yl)methyl)-1-((*S*)-sec-butyl)-8-chloro-1,2-dihydro-6*H*-pyrazino[2,1-*b*]quinazoline-3,6(4*H*)-dione (**24**). (20, 300v)

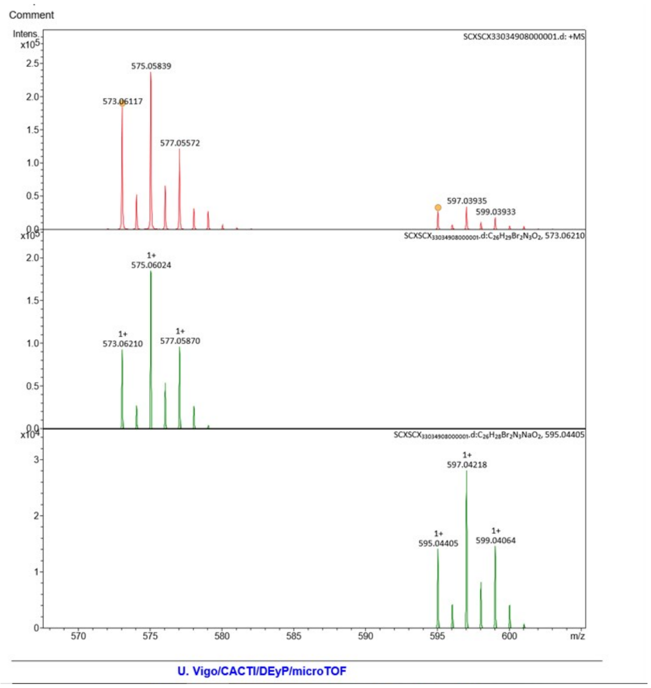

Fig. S51: Mass spectra of (1*S*,4*R*)-4-((1*H*-indol-3-yl)methyl)-8,10-dichloro-1-isopropyl-1,2-dihydro-6*H*-pyrazino[2,1-*b*]quinazoline-3,6(4*H*)-dione (**25**). (20, 300v)

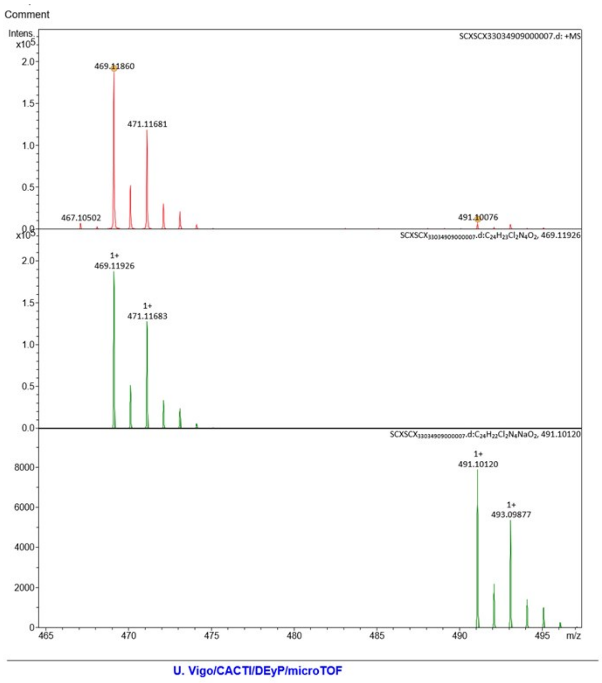

Fig. S52: Mass spectra of (1*S*,4*R*)-4-((1*H*-indol-3-yl)methyl)-8,10-dichloro-1-isobutyl-1,2-dihydro-6*H*-pyrazino[2,1-*b*]quinazoline-3,6(4*H*)-dione (**26**). (20, 300v)

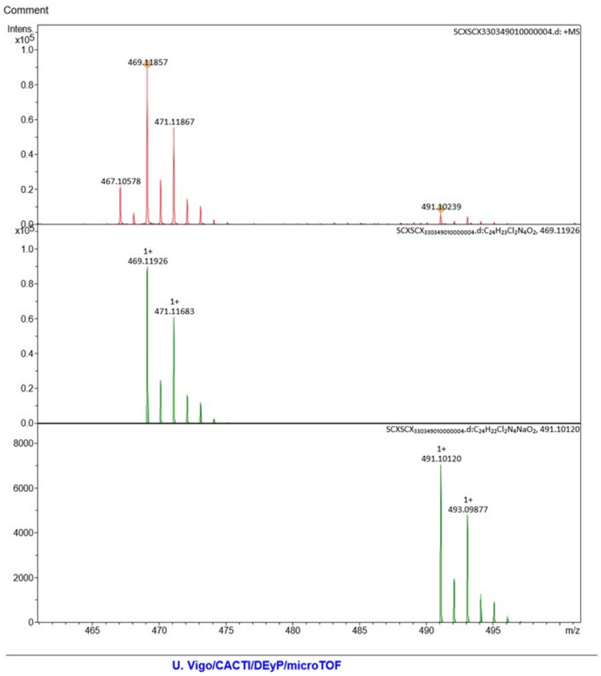

Fig.S53: Mass spectra of (1*S*,4*R*)-4-((1*H*-indol-3-yl)methyl)-1-((*S*)-sec-butyl)-8,10-dichloro-1,2-dihydro-6*H*-pyrazino[2,1-*b*]quinazoline-3,6(4*H*)-dione (**27**). (20, 300v)

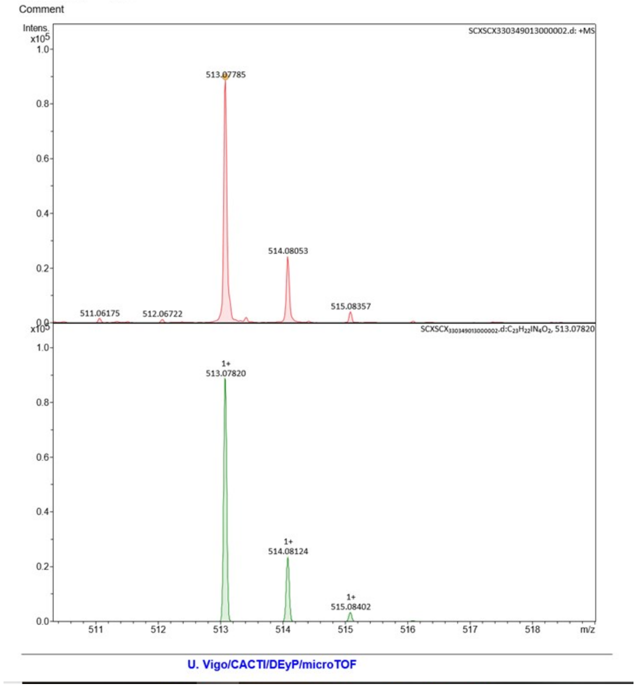

Fig. S54: Mass spectra of (1*S*,4*R*)-4-((1*H*-indol-3-yl)methyl)-8-iodo-1-isopropyl-1,2-dihydro-6*H*-pyrazino[2,1-*b*]quinazoline-3,6(4*H*)-dione (**28**). (20, 300v)

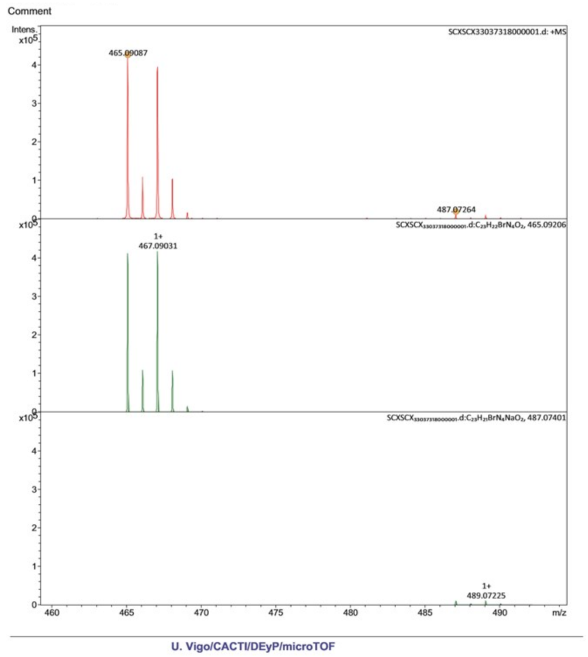

Fig. S55. Mass spectra of (1*S*,4*R*)-4-((1*H*-indol-3-yl)methyl)-8-bromo-1-isopropyl-1,2-dihydro-6*H*-pyrazino[2,1-*b*]quinazoline-3,6(4*H*)-dione (**29**). (20, 300v)

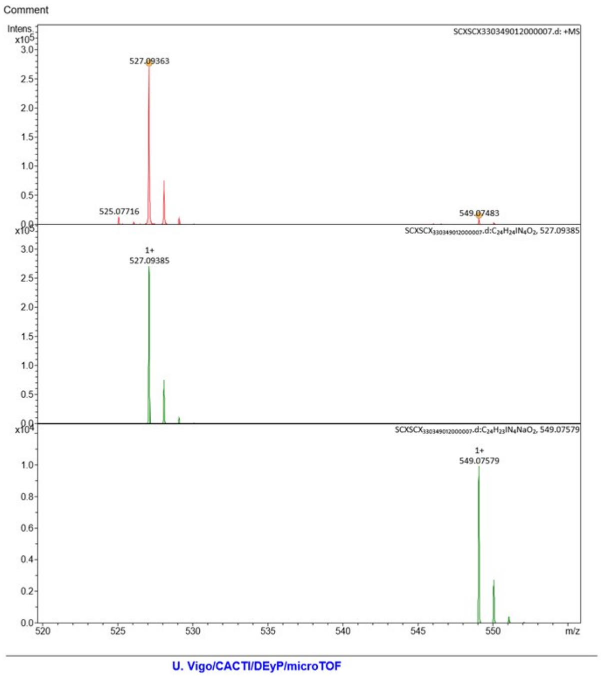

Fig. S56: Mass spectra of (1*S*,4*R*)-4-((1*H*-indol-3-yl)methyl)-8-iodo-1-isobutyl-1,2-dihydro-6*H*-pyrazino[2,1-*b*]quinazoline-3,6(4*H*)-dione (**30**). (20, 300v)

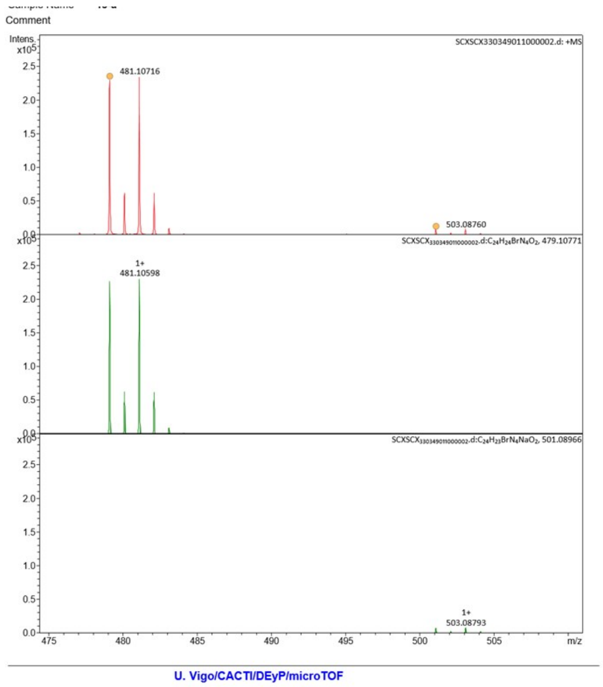

Fig. S57: Mass spectra of (1*S*,4*R*)-4-((1*H*-indol-3-yl)methyl)-8-bromo-1-isobutyl-1,2-dihydro-6*H*-pyrazino[2,1-*b*]quinazoline-3,6(4*H*)-dione (**31**). (20, 300v)

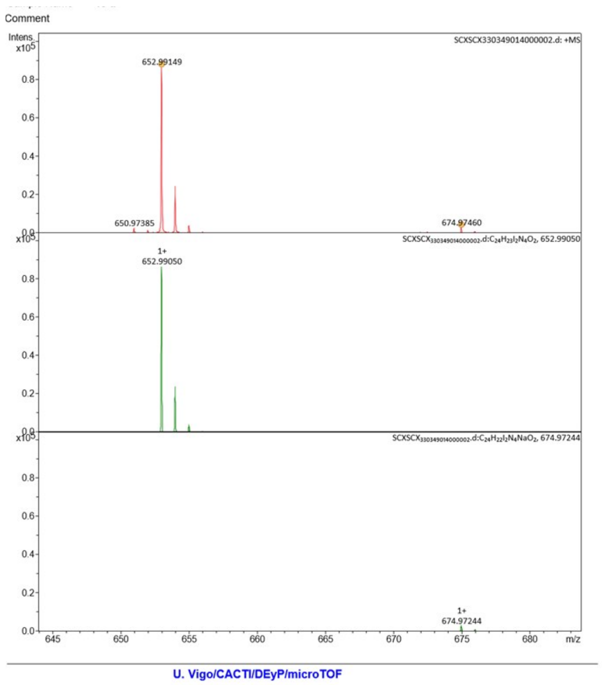

Fig. S58: Mass spectra of (1*S*,4*R*)-4-((1*H*-indol-3-yl)methyl)-8,10-diiodo-1-isobutyl-1,2-dihydro-6*H*-pyrazino[2,1-*b*]quinazoline-3,6(4*H*)-dione (**32**). (20, 300v)

4. Antimicrobial activity

Table S3. Antibacterial activity of quinazolinones **5**-**32** on Gram-positive and Gram-negative sensitive strains. MIC and MBC are expressed in µg/mL. Inhibition halos are expressed in mm.

	**Gram-positive**						**Gram-negative**					
	***S. aureus* ATCC 29213**			***E. faecalis* ATCC 29212**			***E. coli* ATCC 25922**			***P. aeruginosa* ATCC 27853**		
	Halo	MIC	MBC	Halo	MIC	MBC	Halo	MIC	MBC	Halo	MIC	MBC
**5**	0	>64	ND	0	>64	ND	0	>64	ND	0	>64	ND
**6**	0	>64	ND	0	>64	ND	0	>64	ND	0	>64	ND
**7**	0	>64	ND	0	>64	ND	0	>64	ND	0	>64	ND
**8**	0	>64	ND	0	>64	ND	0	>64	ND	0	>64	ND
**9**	0	>64	ND	0	>64	ND	0	>64	ND	0	>64	ND
**10**	0	>64	ND	0	>64	ND	0	>64	ND	0	>64	ND
**11**	0	>64	ND	0	>64	ND	0	>64	ND	0	>64	ND
**12**	0	>64	ND	0	>64	ND	0	>64	ND	0	>64	ND
**13**	0	>64	ND	0	>64	ND	0	>64	ND	0	>64	ND
**14**	0	>64	ND	0	>64	ND	0	>64	ND	0	>64	ND
**15**	0	>64	ND	0	>64	ND	0	>64	ND	0	>64	ND
**16**	0	>64	ND	0	>64	ND	0	>64	ND	0	>64	ND
**17**	0	>64	ND	0	>64	ND	0	>64	ND	0	>64	ND
**18**	0	>64	ND	0	>64	ND	0	>64	ND	0	>64	ND
**19**	0	>64	ND	0	>64	ND	0	>64	ND	0	>64	ND
**20**	0	>64	ND	0	>64	ND	0	>64	ND	0	>64	ND
**21**	0	>64	ND	**9***	>64	ND	0	>64	ND	0	>64	ND
**22**	**9**	**>32**	> 64	0	>64	>64	0	>64	ND	0	>64	ND
(-)-**22**	**9**	>64	ND	0	>64	ND	0	>64	ND	0	>64	ND
(+)-**22**	**9**	>64	ND	0	>64	ND	0	>64	ND	0	>64	ND
**23**	**9**	**>32**	> 64	0	**>32**	>64	0	>64	ND	0	>64	ND
(+)-**23**	**10**	>64	ND	0	>64	ND	0	>64	ND	0	>64	ND
**24**	**9**	**>16**	> 64	0	**>32**	>64	0	>64	ND	0	>64	ND
**25**	**11**	**>16**	> 64	**9.5***	>64	ND	**8***	>64	ND	0	>64	ND
**26**	**11**	**>4**	> 64	**11***	>64	ND	**8***	>64	ND	0	>64	ND
**(-)-26**	**11**	**>4**	>64	**9**	>64	ND	0	>64	ND	0	ND	ND
(+)-**26**	**11**	>64	ND	**8.5**	>64	ND	0	>64	ND	0	ND	ND
**27**	**10**	**>4**	> 64	**10***	>64	ND	**8***	>64	ND	0	>64	ND
**28**	**9***	> 64	ND	0	>64	ND	**8.5***	>64	ND	**8.5***	>64	ND
**29**	0	> 64	ND	0	>64	ND	0	>64	ND	**8.5***	>64	ND
**30**	**9.5**	**>16**	> 64	**9.5***	>64	ND	**8**	>64	ND	0	>64	ND
**31**	**9.5**	**>16**	> 64	**10***	>64	ND	**8**	>64	ND	0	>64	ND
**32**	**9**	> 64	ND	**11***	>64	ND	0	>64	ND	0	>64	ND

MIC, minimum inhibitory concentration; MBC, minimum bactericidal concentration; * halo of partial inhibition; ND, not determined

Table S4. Antibacterial activity of quinazolinones **5**-**32** on five different bacterial strains. MIC and MBC are expressed in µg/mL. Inhibition halos are expressed in mm.

	S. aureus **66/1 (MRSA)**			***S. aureus*** **40/61/24**			***E. faecalis*** **B3/101 (VRE)**			***E. coli*** **SA/2**			***E. faecalis*** **A5/102 (VRE)**		
	**Halo**	**MIC**	**MBC**	**Halo**	**MIC**	**MBC**	**Halo**	**MIC**	**MBC**	**Halo**	**MIC**	**MBC**	**Halo**	**MIC**	**MBC**
**5**	0	>64	ND	-	-	-	0	>64	ND	0	>64	ND	-	-	-
**6**	0	>64	ND	-	-	-	0	>64	ND	0	>64	ND	-	-	-
**7**	0	>64	ND	-	-	-	0	>64	ND	0	>64	ND	-	-	-
**8**	0	>64	ND	-	-	-	0	>64	ND	0	>64	ND	-	-	-
**9**	0	>64	ND	-	-	-	0	>64	ND	0	>64	ND	-	-	-
**10**	0	>64	ND	-	-	-	0	>64	ND	0	>64	ND	-	-	-
**11**	0	>64	ND	-	-	-	0	>64	ND	0	>64	ND	-	-	-
**12**	0	>64	ND	-	-	-	0	>64	ND	0	>64	ND	-	-	-
**13**	0	>64	ND	-	-	-	**7**	>64	ND	**7**	>64	ND	-	-	-
**14**	0	>64	ND	-	-	-	**8**	>64	ND	0	>64	ND	-	-	-
**15**	0	>64	ND	-	-	-	**8**	>64	ND	0	>64	ND	-	-	-
**16**	0	>64	ND	-	-	-	**8**	>64	ND	0	>64	ND	-	-	-
**17**	0	>64	ND	-	-	-	0	>64	ND	0	>64	ND	-	-	-
**18**	0	>64	ND	-	-	-	0	>64	ND	0	>64	ND	-	-	-
**19**	0	>64	ND	-	-	-	0	>64	ND	0	>64	ND	-	-	-
**20**	0	>64	ND	-	-	-	0	>64	ND	0	>64	ND	-	-	-
**21**	0	>64	ND	-	-	-	0	>64	ND	0	>64	ND	-	-	-
**22**	0	>64	ND	ND	64	>64	**9**	>64	ND	**7**	ND	ND	ND	>64	>64
(-)-**22**	**9**	ND	ND	-	-	-	0	ND	ND	0	ND	ND	-	-	-
(+)-**22**	**9**	ND	ND	-	-	-	0	ND	ND	0	ND	ND	-	-	-
**23**	0	>64	ND	ND	64	>64	**8**	>64	ND	**7**	ND	ND	ND	>64	>64
(+)-**23**	0	ND	ND	-	-	-	0	ND	ND	0	ND	ND	-	-	-
**24**	**9**	>64	ND	ND	64	>64	**8**	>64	ND	**7**	ND	ND	ND	>64	>64
**25**	**10**	>64	ND	ND	>64	ND	0	ND	ND	0	ND	ND	ND	ND	ND
**26**	**10**	**>8**	>64	ND	**>8**	>64	0	ND	ND	0	ND	ND	ND	ND	ND
**(-)-26**	0	**>4**	>64	ND	**>4**	>64	0	ND	ND	0	ND	ND	ND	ND	ND
(+)-**26**	0	ND	ND	-	-	-	0	ND	ND	0	ND	ND	-	-	-
**27**	**9.5**	**>8**	>64	ND	**>8**	>64	0	ND	ND	**9**	ND	ND	ND	ND	ND
**28**	**10**	ND	ND	-	-	-	0	ND	ND	0	ND	ND	-	-	-
**29**	0	ND	ND	-	-	-	0	ND	ND	0	ND	ND	-	-	-
**30**	**9***	>64	ND	ND	64	>64	0	ND	ND	0	ND	ND	ND	ND	ND
**31**	**10***	>64	ND	ND	>64	ND	0	ND	ND	**8.5**	ND	ND	ND	ND	ND
**32**	0	ND	ND	-	-	-	0	ND	ND	0	ND	ND	-	-	-

MIC, minimum inhibitory concentration; MBC, minimum bactericidal concentration; * halo of partial inhibition; ND, not determined

Table S5. Antifungal activity of quinazolines **5**-**32** against a panel of yeast and filamentous fungi. MIC and MFC are expressed in µg/mL.

	***C. albicans* ATCC 10231**		**A. *fumigatus* ATCC 46645**		***T. rubrum* FF5**		***M. canis* FF1**		***E. floccosum* FF9**	
	**MIC**	**MFC**	**MIC**	**MFC**	**MIC**	**MFC**	**MIC**	**MFC**	**MIC**	**MFC**
**5**	> 128	ND	> 128	ND	> 128	ND	**ND**	ND	ND	ND
**6**	> 128	ND	> 128	ND	> 128	ND	ND	ND	ND	ND
**7**	> 128	ND	> 128	ND	> 128	ND	ND	ND	ND	ND
**8**	> 128	ND	> 128	ND	> 128	ND	ND	ND	ND	ND
**9**	> 128	ND	> 128	ND	> 128	ND	ND	ND	ND	ND
**10**	> 128	ND	> 128	ND	> 128	ND	ND	ND	ND	ND
**11**	> 128	ND	> 128	ND	> 128	ND	ND	ND	ND	ND
**12**	> 128	ND	> 128	ND	> 128	ND	ND	ND	ND	ND
**13**	> 128	ND	> 128	ND	> 128	ND	ND	ND	ND	ND
**14**	> 128	ND	> 128	ND	> 128	ND	ND	ND	ND	ND
**15**	> 128	ND	> 128	ND	> 128	ND	ND	ND	ND	ND
**16**	> 128	ND	> 128	ND	> 128	ND	ND	ND	ND	ND
**17**	> 128	ND	> 128	ND	> 128	ND	ND	ND	ND	ND
**18**	> 128	ND	> 128	ND	> 128	ND	ND	ND	ND	ND
**19**	> 128	ND	> 128	ND	> 128	ND	ND	ND	ND	ND
**20**	> 128	ND	> 128	ND	> 128	ND	ND	ND	ND	ND
**21**	> 128	ND	> 128	ND	> 128	ND	ND	ND	ND	ND
**22**	> 128	ND	> 128	ND	> 128	ND	ND	ND	ND	ND
(-)-**22**	> 128	ND	> 128	ND	> 128	ND	ND	ND	ND	ND
(+)-**22**	> 128	ND	> 128	ND	> 128	ND	ND	ND	ND	ND
**23**	> 128	ND	> 128	ND	> 128	ND	ND	ND	ND	ND
(+)-**23**	> 128	ND	> 128	ND	> 128	ND	ND	ND	ND	ND
**24**	> 128	ND	> 128	ND	> 128	ND	ND	ND	ND	ND
**25**	> 128	ND	> 128	ND	> 128	ND	ND	ND	ND	ND
**26**	> 128	ND	> 128	ND	**128**	>128	>128	ND	>128	ND
**(-)-26**	>128	ND	>128	ND	>128	ND	ND	ND	ND	ND
(+)-**26**	>128	ND	>128	ND	>128	ND	ND	ND	ND	ND
**27**	> 128	ND	> 128	ND	> 128	ND	ND	ND	ND	ND
**28**	> 128	ND	> 128	ND	**128**	>128	>128	ND	>128	ND
**29**	> 128	ND	> 128	ND	**128**	>128	>128	ND	>128	ND
**30**	> 128	ND	> 128	ND	> 128	ND	ND	ND	ND	ND
**31**	> 128	ND	> 128	ND	> 128	ND	ND	ND	ND	ND
**32**	> 128	ND	> 128	ND	> 128	ND	ND	ND	ND	ND

MIC, minimum inhibitory concentration; MFC, minimum fungicidal concentration; ND, not determined

Table S6: Calculated molecular properties of compounds **5-32**.

Comp.	MW[a]	LOP[b]	TPSA (Å^2)[c]	nON[d]	nONNH[e]	nrotb[f]	LIP[g]	PK[h]									BiAS[i]	WatS[j]
								**GIab**	**BBB**	**P-gp**	**CYP1A2**	**CYP2C19**	**CYP2C9**	**CYP2D6**	**CYP3A4**	**Log Kp**		
**5**	386.45	3.1	79.78	3	2	3	Yes	High	No	Yes	No	Yes	Yes	Yes	Yes	-6.02 cm/s	0.55	Moderate
**6**	386.45	3.11	79.78	3	2	3	Yes	High	No	Yes	No	Yes	Yes	Yes	Yes	-6.02 cm/s	0.55	Moderate
**7**	386.45	3.1	79.78	3	2	3	Yes	High	No	Yes	No	Yes	Yes	Yes	Yes	-6.02 cm/s	0.55	Moderate
**8**	386.45	3.11	79.78	3	2	3	Yes	High	No	Yes	No	Yes	Yes	Yes	Yes	-6.02 cm/s	0.55	Moderate
**9**	400.47	3.38	79.78	3	2	4	Yes	High	No	Yes	No	Yes	Yes	Yes	Yes	-5.86 cm/s	0.55	Moderate
**10**	400.47	3.41	79.78	3	2	4	YEs	High	No	Yes	No	Yes	Yes	Yes	Yes	-5.86 cm/s	0.55	Moderate
**11**	400.47	3.42	79.78	3	2	4	Yes	High	No	Yes	No	Yes	Yes	Yes	Yes	-5.86 cm/s	0.55	Moderate
**12**	400.47	3.41	79.78	3	2	4	Yes	High	No	Yes	No	Yes	Yes	Yes	Yes	-5.86 cm/s	0.55	Moderate
**13**	400.47	3.43	79.78	3	2	4	Yes	High	No	Yes	No	Yes	Yes	Yes	Yes	-5.73 cm/s	0.55	Moderate
**14**	400.47	2.43	79.78	3	2	4	Yes	High	No	Yes	No	Yes	Yes	Yes	Yes	-5.73 cm/s	0.55	Moderate
**15**	418.51	3.07	105.08	3	2	5	Yes	High	No	Yes	No	Yes	Yes	Yes	Yes	-6.45 cm/s	0.55	Moderate
**16**	418.51	3.1	105.08	3	2	5	Yes	High	No	Yes	No	Yes	Yes	Yes	Yes	-6.45 cm/s	0.55	Moderate
**17**	540.61	4.85	89.01	4	2	7	Yes*	High	No	No	Yes	No	Yes	No	Yes	-5.48 cm/s	0.55	Insoluble
**18**	540.61	4.83	89.01	4	2	7	Yes*	High	No	No	Yes	No	Yes	No	Yes	-5.48 cm/s	0.55	Insoluble
**19**	450.49	3.23	100.01	4	3	4	Yes	High	No	Yes	No	Yes	Yes	Yes	No	-6.22 cm/s	0.55	Moderate
**20**	450.49	3.27	100.01	4	3	4	Yes	High	No	Yes	No	Yes	Yes	Yes	No	-6.22 cm/s	0.55	Moderate
**21**	358.39	2.55	79.78	3	2	2	Yes	High	No	Yes	No	Yes	Yes	Yes	No	-6.42 cm/s	0.55	Moderate
**22**	420.89	3.63	79.78	3	2	3	Yes	High	No	Yes	No	Yes	Yes	No	Yes	-5.79 cm/s	0.55	Moderate
**23**	434.92	3.95	79.78	3	2	4	Yes	High	No	Yes	No	Yes	Yes	No	Yes	-5.62 cm/s	0.55	Poor
**24**	434.92	3.99	79.78	3	2	4	Yes	High	No	Yes	No	Yes	Yes	No	Yes	-5.49 cm/s	0.55	Poor

Comp.	MW[a]	LOP[b]	TPSA (Å^2)[c]	nON[d]	nONNH[e]	nrotb[f]	LIP[g]	PK[h]									BiAS[i]	WatS[j]
**25**	434.92	4.12	79.78	3	2	3	Yes	High	No	Yes	No	Yes	Yes	No	Yes	-5.56 cm/s	0.55	Poor
**26**	469.36	4.44	79.78	3	2	4	Yes	High	No	Yes	No	Yes	Yes	No	Yes	-5.39 cm/s	0.55	Poor
**27**	469.36	4.5	79.78	3	2	4	Yes	High	No	Yes	No	Yes	Yes	No	Yes	-5.26 cm/s	0.55	Poor
**28**	512.34	3.75	79.78	3	2	3	Yes	High	No	Yes	No	Yes	Yes	No	Yes	-6.33 cm/s	0.55	Poor
**29**	465.34	3.72	79.78	3	2	3	Yes	High	No	Yes	No	Yes	Yes	No	Yes	-6.01 cm/s	0.55	Poor
**30**	526.37	4.06	79.78	3	2	4	Yes	High	No	Yes	No	Yes	Yes	No	Yes	-6.17 cm/s	0.55	Poor
**31**	479.37	4.04	79.78	3	2	4	Yes	High	No	Yes	No	Yes	Yes	No	Yes	-5.85 cm/s	0.55	Poor
**32**	652.27	4.68	79.78	3	2	4	No	High	No	Yes	No	Yes	Yes	No	No	-6.47 cm/s	0.55	Poor

[a]MW =Molecular weight (g/mol)
[b] LOP = octanol-water partition coefficient (consensus log $P_{o/w}$)
[c] TPSA = Topological polar surface area (Å^2)
[d] nON = number of hydrogen bond acceptors
[e] nONNH = number of hydrogen bond donors
[f] nrotb = number of rotatable bonds
[g] LIP = Lipinski (* yes with one violation)
[f] PK = Pharmacokinetics (GIab = GI absorption, BBB = BBB permeant, P-gp = P-go substrate, CYP = CYP inhibitor, Long Kp (skin permeataion)
[I] BiAS = Bioavailability score
[j] WatS = Water solubility (SILICOS-IT)
*All calculation were obtained from Swiss Institute of Bioinformatic (SwissADME): available at http://www.swissadme.ch/index.php (accessed 6th march 2020)
